# The Sherpa hypothesis: Phenotype-Preserving Disordered Proteins stabilize the phenotypes of neurons and oligodendrocytes

**DOI:** 10.1038/s41540-023-00291-8

**Published:** 2023-07-11

**Authors:** Vic Norris, Judit Oláh, Sergey N. Krylov, Vladimir N. Uversky, Judit Ovádi

**Affiliations:** 1 Laboratory of Microbiology Signals and Microenvironment, University of Rouen, 76821 Mont Saint Aignan, France; 2 Institute of Enzymology, Research Centre for Natural Sciences, Budapest, H-1117 Hungary; 3 Centre for Research on Biomolecular Interactions, York University, Toronto, ON M3J1P3 Canada; 4 Department of Molecular Medicine, Morsani College of Medicine, University of South Florida, Tampa, FL 33612 USA

**Keywords:** Systems biology, Neuroscience

## Abstract

Intrinsically disordered proteins (IDPs), which can interact with many partner proteins, are central to many physiological functions and to various pathologies that include neurodegeneration. Here, we introduce the *Sherpa* hypothesis, according to which a subset of stable IDPs that we term Phenotype-Preserving Disordered Proteins (PPDP) play a central role in protecting cell phenotypes from perturbations. To illustrate and test this hypothesis, we computer-simulate some salient features of how cells evolve and differentiate in the presence of either a single PPDP or two incompatible PPDPs. We relate this virtual experiment to the pathological interactions between two PPDPs, α-synuclein and Tubulin Polymerization Promoting Protein/p25, in neurodegenerative disorders. Finally, we discuss the implications of the *Sherpa* hypothesis for aptamer-based therapies of such disorders.

## Introduction

Intrinsically disordered proteins (IDPs) transiently adopt many different conformations that range from the compact to the extended forms unhindered by energetic constraints^1^. IDPs are common, with the eukaryotic proteome estimated to contain around ∼20% disordered proteins^2,3^. Disordered regulatory domains can constitute switches as their structures can be changed significantly by post-translational modifications^4^. IDPs and proteins that contain intrinsically disordered regions, which for convenience we class here as IDPs, are implicated in liquid-liquid phase separation and the formation of intracellular biomolecular condensates^5,6^. IDPs are frequently involved in cell signalling networks because they can bind to many partner proteins with both high specificity and low affinity (hence transiently)^1,7,8^; these disorder-based interactions can take several forms^1,9,10^. Some IDPs display a tendency to self-associate and to form stable aggregates^11,12^.

IDPs are frequently implicated in various maladies, such as cancer and neurodegenerative diseases^13^. For example, mutations in the disordered protein p53 have been identified in colon, lung, breast, and brain cancers^14^. Protein aggregation in the brain is central to neurodegenerative diseases, with α-synuclein (SYN) and Tubulin Polymerization Promoting Protein/p25 (TPPP) aggregation being characteristic of Parkinson’s disease and multiple system atrophy^15–17^, and β-amyloid and tau aggregation being characteristic of Alzheimer’s disease^18,19^. Aggregation of these unfolded/misfolded proteins generally leads to the formation of characteristic proteinaceous deposits in neurodegenerative diseases^20^.

Despite the well-established importance of many IDPs for pathogenesis, the normal function of these proteins is not entirely clear. Indeed, understanding their physiological functions might allow the development of therapies without side-effects. It has been argued that one advantage of disorder may be that it makes an economical use of resources because the interface between an IDP and a partner protein requires fewer residues from the IDP than a similar interface between ordered proteins^21^. It is also believed that the flexibility of IDPs enables them to perform functions that are complementary to those of ordered proteins^22^. The structural heterogeneity and conformational plasticity of IDPs constitute the foundation of the structure-function continuum model, in which a protein exists as a dynamic conformational ensemble containing multiple proteoforms characterized by a broad spectrum of structural features and possessing various functional potentials^23–25^, with SYN being considered as one of the illustrative examples of this protein structure-function continuum model^26^.

Fully understanding the function of a protein may well require understanding the living system within which this protein operates. One approach to understanding living systems is to model them as networks of connections between elements or nodes. In a pioneering investigation of such networks, Kauffman highlighted a fundamental problem that confronts cells containing many interacting constituents, namely, how such cells manage to obtain the reproducible phenotypes needed for natural selection to be effective when a ‘hyper-astronomical’ number of combinations of these constituents is apparently available^27^. The solution we favour is to consider the cell as a set of elements or macromolecules, out of which only a few – rather than a number limited only by the total number of constituents – are selected to determine the cell’s behaviour or phenotype at any given time^28,29^. This subset of macromolecules, which determines the phenotype, is selected on the basis that: i) the subset must be coherent (i.e., the macromolecules in this subset must work together and not against one another); ii) the behaviour at one time must be coherent with the behaviour at the previous time (i.e., any behaviour-determining subset of macromolecules must continue the work of the previous subset).

Here, we propose a hypothesis for the function of stable IDPs inspired by Himalayan mountain guides or Sherpas. A Sherpa does more than helping to carry the material needed for an expedition; he can act as an indispensable guide for those climbing a particular mountain, choosing the route and keeping the climbers on it despite perturbations. Climbing different mountains requires different Sherpas. In our hypothesis, one class of the IDPs, Phenotype-Preserving Disordered Proteins (PPDPs), limits the phenotypes available to cells so they can differentiate. The PPDP achieves this restriction of phenotype space by its interactions with its partner proteins and by its own stability, which allows it to act as a kind of memory. To illustrate and validate the hypothesis, we introduce the equivalent of an PPDP into a version of a learning program, *Coco*, which simulates how a cell evolves into a differentiated state via a competition between selection for a state that is internally coherent (i.e., that the constituents work together) and selection for a historical coherence (i.e., that the relationship between successive states is meaningful). We find that even in this very simple, easy-to-analyse, simulation of cell evolution, a PPDP-like element can have a selectable function that protects the phenotype. We also find biological evidence in the neurodegenerative pathways of SYN and TPPP consistent with the Sherpa hypothesis and, finally, we mention the implications for therapy.

## Results

### The Sherpa hypothesis

**Fig. 1:**
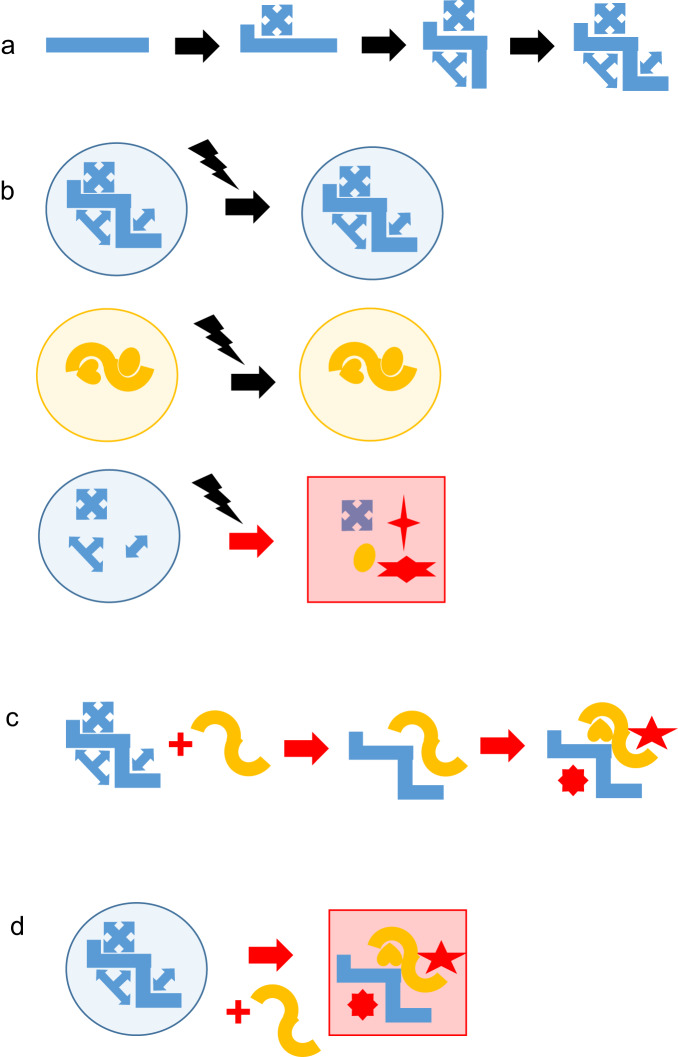
The Sherpa hypothesis. A PPDP plays its physiological role when it interacts with its physiological partners to activate or inactivate them and to co-locate them via phenotype-determining interactions. **a** In physiological conditions, the structure of a PPDP changes progressively (initially a blue rectangle and finally a blue zigzag shape) as its partner proteins (blue rectilinear shapes) are associated with it. **b** Top panel, the PPDP maintains the differentiated state of the cell despite a perturbation (black lightning); middle panel, a different PPDP (thick, dark yellow, curved line) with different partner proteins (dark yellow curved shapes) maintains a different differentiated state despite a perturbation; bottom panel, in the absence of a PPDP, the differentiated state (circular cell) is lost (square red cell corresponding to the absence of blue partner proteins and presence of red stars and of an inappropriate, dark yellow, partner). **c** If two different PPDP bind to one another, the appropriate partner proteins (blue and dark yellow) no longer bind whilst other, inappropriate, proteins (red) may bind. **d** In pathological conditions, the introduction of a second PPDP (dark yellow) causes a loss of the phenotype (red square). Black arrow, physiological path; red arrow, pathological path; red stars, proteins that should not be present in either of the physiological conditions.

We propose that a PPDP can play a central role in the determination of phenotypes by guiding the cell through successive patterns or trajectories of gene and protein activity and by helping maintain these patterns despite perturbations (Fig. 1). The phenotype-stabilising actions of the PPDPs are achieved because these ‘chameleon’ proteins undergo a succession of conformational changes via their binding to different partners (not necessarily in a particular order) as determined by the intracellular conditions. In this scenario, the first partner to bind the PPDP reduces the conformational space available to the PPDP so that only a subset of the original conformations become available (and perhaps some new conformations that were not in the original space), then the second partner binds and reduces that space still further. This sequence of conformational changes may sometimes be accompanied by a sequence of post-translational modifications to the PPDP and its partners that determines the functioning of the ensemble of these proteins. The result is a phenotype-determining, multi-partner assembly. The binding of a different first partner would send the PPDP off into a different region of conformational space where the PPDP’s function would be altered. If the cell were subjected to a major perturbation, the PPDP would help maintain the cell in its differentiated state or trajectory because it binds, brings together, and can protect (or inactivate) its partners, which are necessary for the differentiation. In the Sherpa hypothesis, different PPDPs can be associated with different, incompatible, differentiated states; the downside of this is that the introduction of a second PPDP into a cell could result in a serious perturbation of its phenotype; if this second PPDP were then removed, the first PPDP would then help restore the phenotype.

### The *Coco* program: Learning vs. phenotype

**Fig. 2:**
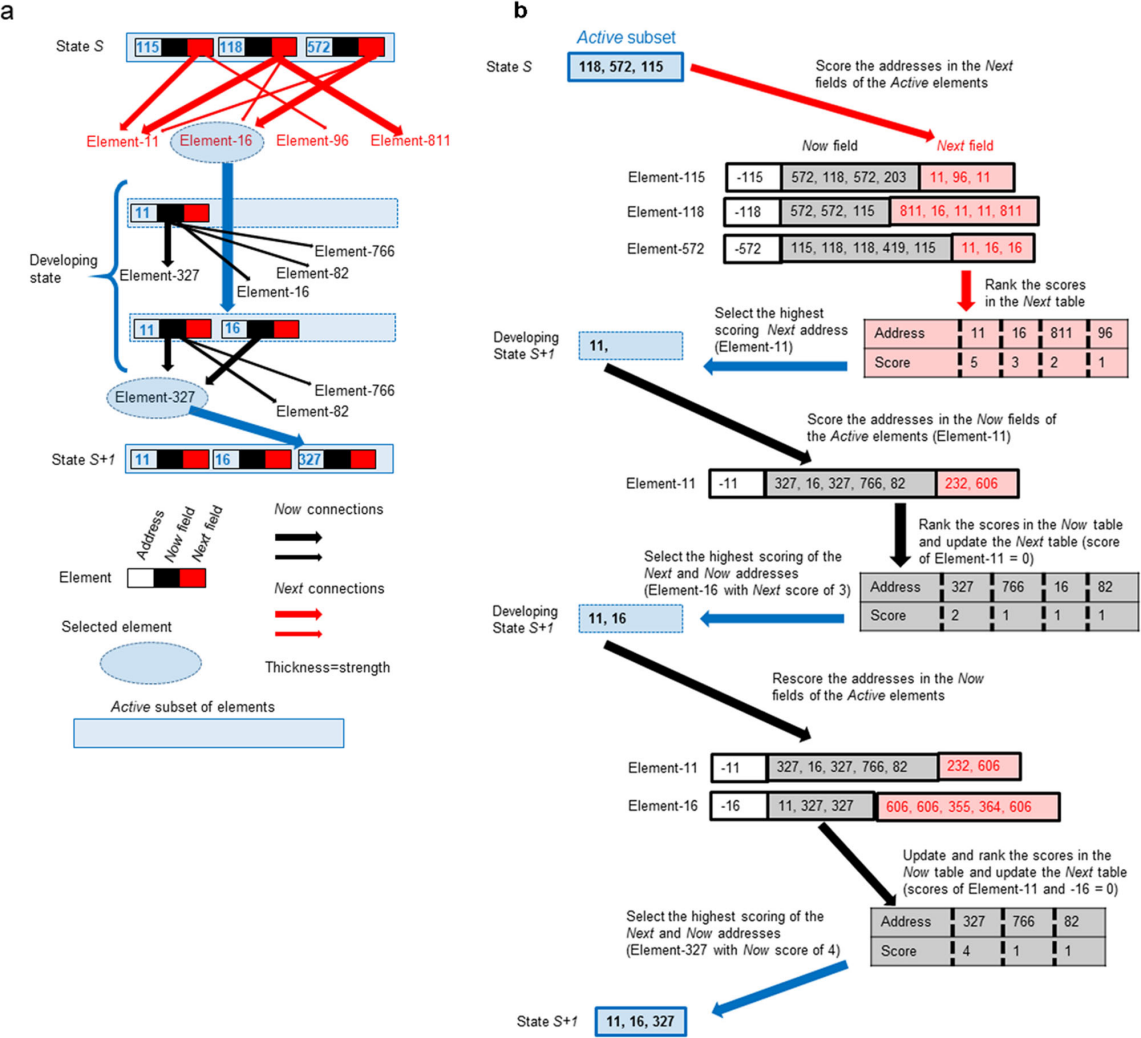
The principle of the program. **a** The phenotypic state of the cell is represented by the contents of the *Active* subset, which here contains only three elements (for simplicity). The composition of the *Active* subset is determined by a competition between the *Now* and *Next* connectivities. Initially, the *Next* connectivity (red arrows), which represents the coherence of phenotypic states over time, is used to select the first element (e.g., macromolecule) for membership of the developing state of the *Active* subset. Then, the *Now* connectivity (black arrows), which represents the coherence of the phenotypic state at a particular time, is also used to select the elements. The composition of the *Active* subset after learning (alias evolution by natural selection) is a solution to the problem of satisfying the two types of coherence. **b** Details of how consecutive states of the *Active* subset are calculated. Note that the sizes of the *Now* and *Next* fields varies. The *Now* table of element addresses and their scores is grey and those of the *Next* table are pink.

To investigate the possible roles of proteins that are stable and that can interact with many partners in preserving the phenotype, we model the role of the interactions of a subset of macromolecules in determining the phenotype of the cell at any given time. In this modelling, we use a previously published, machine-learning program, *Coco*^28,29^, that simulates, at a very simple level, how a cell evolves into a differentiated state (Fig. 2). The idea here is to show that, during a learning task mimicking natural selection, conditions can exist that select a PPDP-like element. This program can have thousands of elements that can mimic macromolecules such as proteins; each element can be either *active* or *inactive* in determining the behaviour of the system in a time-step; an element is *active* if it is a member of the current state of the *Active* subset of elements (and inactive if it is not). Although *Coco* can learn with a state of the *Active* subset containing from three to over a hundred elements (not shown), for convenience, in this version, the state of the *Active* subset contains only six elements selected from a total set of a thousand possible elements.

The composition of a state of the *Active* subset in terms of elements changes at each time step. Membership of a state of the *Active* subset is made based on connectivity; each element has two fields, a *Now* field and a *Next* field, that contain the addresses of other elements; the presence of these addresses in an element’s fields correspond to the connections that this element makes with other elements. The strength of the *Next* connections that Element-X makes to Element-Y is calculated by counting the number of times the address of Element-Y occurs in the *Next* field of Element-X (and similarly for its *Now* field). The membership of the *Active* subset is calculated for each time step by ranking the strengths of the connections of the *Next* fields of the elements in the present *Active* subset. This entails summing the number of times the address of a particular element occurs in the *Next* fields of all the elements in the present *Active* subset, then ranking these sums, then selecting the element with the highest *Next* rank and comparing it with the element with the highest *Now* rank. This latter element is chosen via the connections in the *Now* fields of those elements already selected as members of the new developing *Active* subset. Note that the same address can occur in both the *Next* and *Now* fields, but this is kept separate in the ranking. To put it differently, the score of the element at the top of the *Next* ranking is compared with the score of the element at the top of the *Now* ranking and the element with the higher score is then elected for membership of the *Active* subset. This coherence competition between the *Next* and *Now* rankings results in the system going through a sequence of states of the *Active* subset. By appropriately rewarding and punishing the contents of the states of the *Active* subsets (i.e., by changing the addresses in the fields of the *Active* elements), the system can learn to respond to its environment in an evolutionary process that leads to a sequence of states of *Active* subsets equivalent to differentiation.

### Simulation: A PPDP constrains phenotype space

**Fig. 3:**
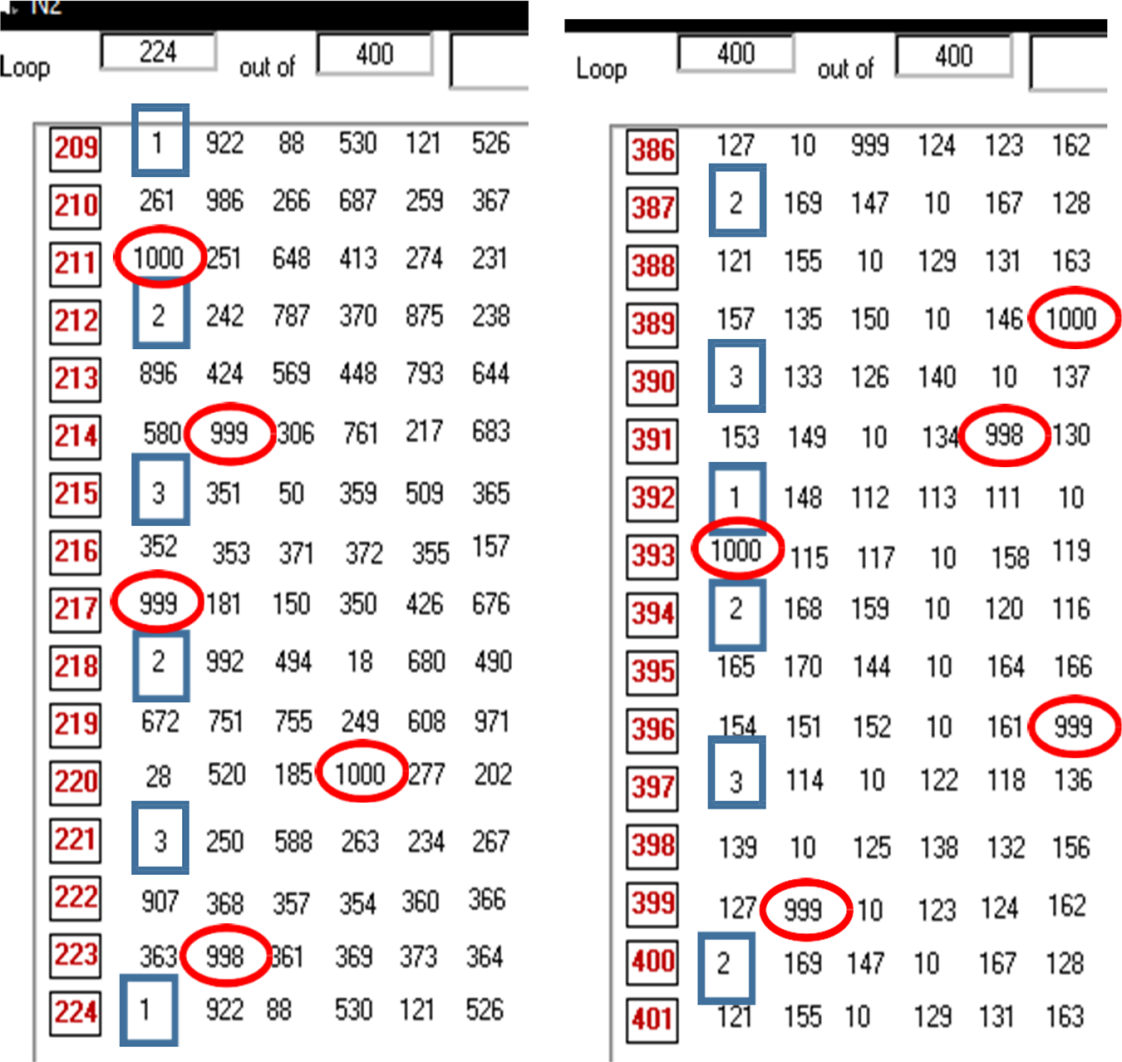
Pattern of learning altered by a stable, highly connected PPDP-like element. Successive states of the *Active* subset, each containing six elements, after learning. Each line of six elements corresponds to the state of the phenotype at a particular time; successive lines correspond to the trajectory of the phenotypes in the order of the line numbers; the inputs from the environment are in blue rectangles whilst the responses of the system are in red circles. Left panel: Learning without a PPDP-like element results in the selection of addresses of elements from the entire range of the thousand elements in the program. Right panel: Learning with the PPDP-like Element-**10** results in the selection of addresses of elements exclusively from Elements-**111** to **169**, which are just those in the range contained in the *Now* and *Next* fields of Element-**10**. Input elements are in blue squares and outputs in red ovals. The column of red numbers are the states of the *Active* subset.

The acquisition of a subset of phenotypes or phenotype trajectory by a cell via natural selection during its evolution was simulated by *Coco*. This program learns to select its constituent elements (alias ‘macromolecules’) to be in the subset of elements that determines the system’s behaviour in successive time-steps; this selection is based on the connections between elements; these connections result from reward and punishment strategies that strengthen or weaken these connections (cf. Methods). *Coco* was given the task of learning to respond correctly to the input sequence of (1, 2, 3, 2, 3) from the environment with an output sequence of (1000, 999, 999, 1000, 998) to that environment; in these input-output sequences, *Coco* has to learn to respond to the input of Element-1 with an output of Element-1000, this is followed by an input of Element-2 and a learnt output of Element-999 etc. In the absence of a PPDP-like element, the *active* elements (i.e., those with their addresses in the *Active* subset) were drawn from the entire set of 1000 elements (Fig. 3).

However, in the presence of a PPDP-like element (Element-10), all the *active* elements were drawn from the elements connected to the PPDP-like element, that is, the addresses of all these elements were in the *Now* and *Next* fields of Element-10 (fields that only contain addresses 111 to 170) and no other elements were *active* (Fig. 3). This shows how the presence of a stable, highly connected element in *Coco* can steer its evolution so its states involve only a very limited subset of the elements available. One implication of this result is that the presence of a stable, potentially highly connected, PPDP in an evolving cell or protocell limits the range of regulatory molecules responsible for its phenotype. A related implication is that a set of different, incompatible PPDPs with different connections could favour the selection of different phenotypes (or more exactly phenotype trajectories) during evolution; this is because this situation would help avoid confusion due to the same molecules being involved in incompatible phenotypes. In other words, there appears to be a good reason why PPDPs should have been selected during evolution.

### A PPDP helps preserve a phenotype

Once a PPDP has been selected during the evolution, we propose that it is maintained because it helps preserve the phenotype (or trajectory of phenotypes). Such preservation would entail resisting perturbations that could otherwise result in the loss of the phenotype. To illustrate this, we first explored the effects of a major perturbation on *Coco* once it had learnt the standard sequence (corresponding to a trajectory of phenotypes). This perturbation took two forms. Firstly, we reasoned that the number of factors determining the phenotype is important and that it varies with the environment; indeed, in the case of an exponentially growing bacterial population, a change in the environment results in major changes in the number of factors^30^. We therefore simulated this number as the size of the state of the *Active* subset, which we doubled by going abruptly from six elements to twelve. Secondly, in the next time step where there would otherwise be an input of 1 in the *Active* subset, we overwrote this state by creating an entirely new state in which we force into it the addresses of inactive elements. These elements could be considered as corresponding to the regulatory factors appropriate for a different phenotype.

In the absence of a PPDP-like element, the perturbation resulted in a complete loss of the learnt states (Supplementary Fig. 1a). Hardly any of the elements that were present in these states were present after the perturbation; Elements-22 and −122 are exceptions in occurring both before and after the perturbation. In contrast, in the presence of the PPDP-like Element-10, the learnt states are preserved (Supplementary Fig. 1b); for example, all the elements in one of the *Active* states shown (153, 114, 1000, 145, 10 and 119) can be found after the perturbation (Supplementary Fig. 1b). This illustrates how an IDP element can protect learning within the context of *Coco*. The implication of this result is that, for a cell experiencing a major perturbation, a PPDP could protect its phenotype or phenotype trajectory and maintain its differentiated state.

In the Sherpa hypothesis, we propose that the downside of a PPDP being important for the maintenance of the phenotype is that the presence of a second PPDP could be disruptive. Indeed, if this second PPDP were important for a different phenotype incompatible with the first phenotype, there could be a total loss of meaningful phenotypes, which would be characterised by a maladaptive, incoherent physiology and behaviour. To illustrate this proposal, we used a version of *Coco* in which a network of a second PPDP element and its connected elements were created from *inactive* elements; this new, second PPDP network mimicked the structure of the first PPDP network. Firstly, we allowed *Coco* to learn in the presence of the PPDP Element-10. Secondly, we introduced a second PPDP element, Element-312, along with some of the elements to which it was connected. From then on, both PPDP elements are present, and the learnt sequence of states is lost (Supplementary Fig. 2). The implication of this result is that if a second PPDP is introduced into a cell in a differentiated state and if the original PPDP and the second PPDP have incompatible sets of partner proteins, the cell may indeed lose its differentiated state.

After the second PPDP element had disrupted the learnt sequence, the probability of this second PPDP element to be reselected for inclusion in the *Active* subset via its *Next* connectivity score was removed by setting this score to zero. This did not necessarily prevent the selection of the second PPDP element and its network because the *Now* connectivity scores of the second PPDP element’s network were not affected. We therefore also forced the selection of one of the environmental inputs (Element-1); this was done only once in time step 1500. The presence of input Element-1 and of the PPDP1 Element-10 together with the absence of the PPDP2 element (Element-640 in Supplementary Fig. 3) led to the recovery of the Elements-118, −127, −137 and −144 (blue arrow) and the restoration of the learnt states, therefore, the removal of the second PPDP can restore the previous phenotype trajectory.

### Model system: TPPP and SYN

**Fig. 4:**
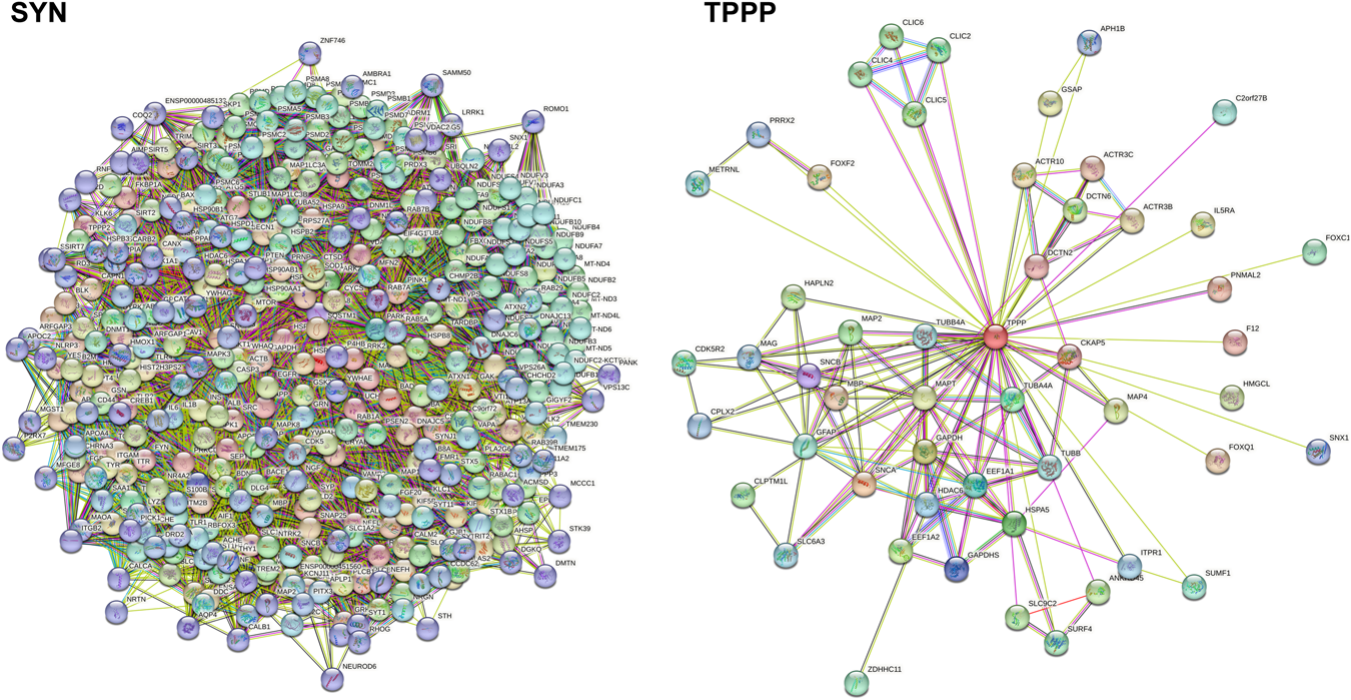
Interaction networks of SYN and TPPP. Left panel: SYN-centred protein-protein interaction network generated by the online database resource Search Tool for the Retrieval of Interacting Genes (STRING)^73^ using confidence of 0.45 for the minimum required interaction score (Table S1). This network contains 401 nodes connected by 8,400 edges. The average node degree of the network is 41.9, and its average clustering coefficient (see Methods) is 0.605. Right panel: TPPP-centred protein-protein interaction network generated by STRING^73^ using confidence of 0.45 for the minimum required interaction score (Table S2). This network contains 52 nodes connected by 147 edges. The average node degree of the network is 5.65, and its average clustering coefficient is 0.826. SYN is denoted by SNCA in the STRING.

TPPP and SYN belong to the IDPs displaying multiple physiological functions and pathological dysfunctions. SYN is expressed endogenously in neurons and is involved in neurological processes such as neurotransmitter release and synaptic plasticity; nevertheless, its precise role in these processes is still unknown^31,32^. TPPP is a key player in the differentiation of the dividing progenitor oligodendrocytes (OLGs) by stabilizing the microtubule network, which provides differentiated cells for the myelination in the central nervous system^33–35^. TPPP inhibits the tubulin deacetylase enzymes histone deacetylase 6 (HDAC6) and sirtuin-2 (SIRT2)^36^ and regulates the microtubule network by its bundling and acetylation-promoting activities. The disordered SYN and TPPP proteins display multifunctional characteristics due to their multiple interactions (Fig. 4 and Supplementary Fig. 4).

**Fig. 5:**
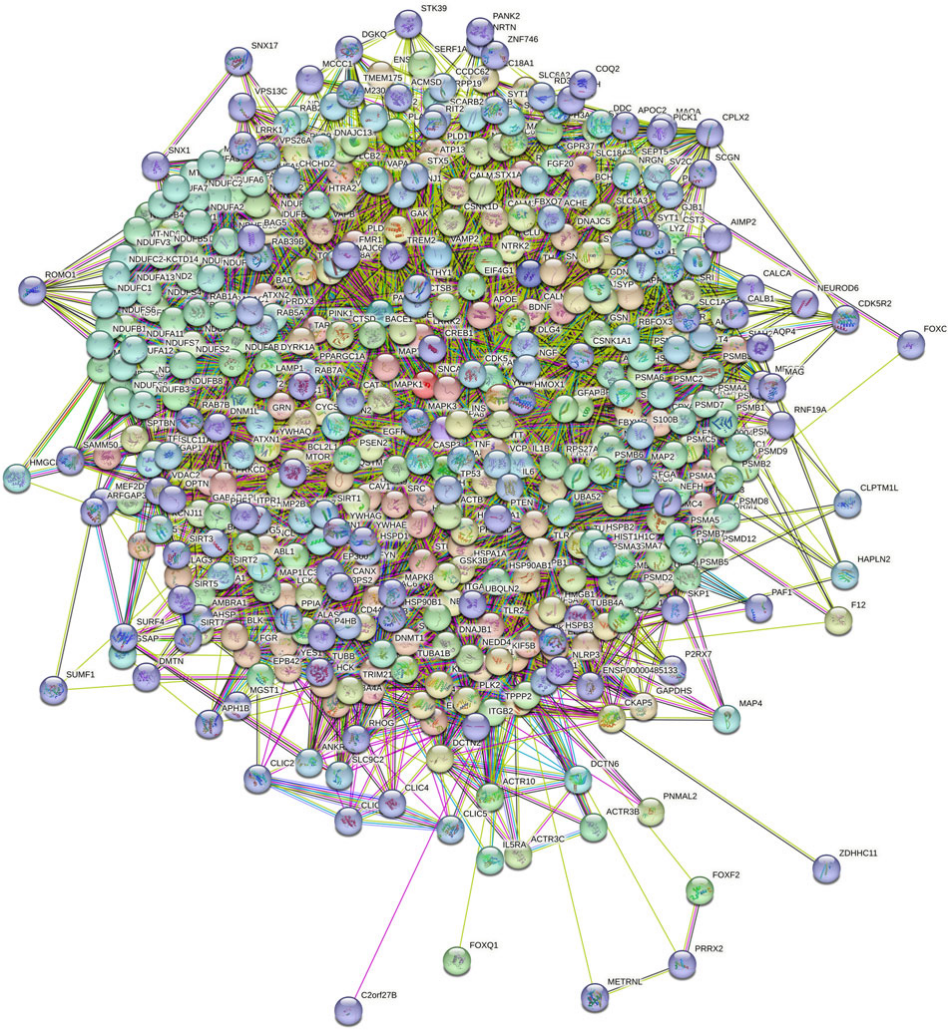
Joint SYN-TPPP protein-protein interaction network. Joint SYN-TPPP-centred protein-protein interaction network generated by STRING^73^ using confidence of 0.45 for the minimum required interaction score (Table S3). This network contains 442 nodes connected by 8866 edges. The average node degree of the network is 40.1, and its average clustering coefficient is 0.5796.

The SYN- and TPPP-centred STRING networks were generated using protein search by name option of STRING using the corresponding UniProt IDs (P37840 and O94811 for SYN and TPPP, respectively) (Fig. 4), whereas the SYN-TPPP network was generated using the Multiple Proteins search mode (Fig. 5). In all cases, the confidence of 0.45 was used for the minimum required interaction score (Excel files with the detailed information are presented in the Supplementary materials as Supplementary Tables 1–3). Figure 4 illustrates the interactions of SYN as well as those of TPPP in both physiological and pathological conditions; however, most of the data refer to pathological circumstances such as the association between SYN and TPPP. The TPPP-derived aggregation of SYN can disrupt the phenotype of the SYN and TPPP networks in both neurons and OLGs. Although the TPPP-centred, protein-protein interaction (PPI) network includes SYN, and although the SYN-centred, PPI network includes TPPP, there are just 9 common proteins in these two networks: glyceraldehyde-3-phosphate dehydrogenase (GAPDH), glial fibrillary acidic protein (GFAP), 78 kDa glucose-regulated protein (HSPA5), HDAC6, microtubule-associated protein tau (MAPT), microtubule-associated protein 2 (MAP2), myelin basic protein (MBP), beta-synuclein (SNCB), and solute carrier family 6 (neurotransmitter transporter, dopamine) member 3 (SLC6A3).

It might be argued that when the PPI networks of two PPDPs are identical (which is not the case for the SYN and TPPP networks) the simultaneous presence of the two PPDPs does not perturb the phenotype whilst when the PPIs are very different such simultaneous presence *does* perturb the phenotype. Simulation of the simultaneous presence of two PPDP-like elements with different networks, such as SYN and TPPP, does indeed result in a loss of the learnt state (Supplementary Fig. 2). The implication of this is that since the PPI networks generated for SYN and TPPP have little overlap, the probability that the introduction of TPPP and the TPPP network into a neuron will disrupt the phenotype of the SYN network (and vice versa for an oligodendrocyte) is greater than the probability that the two networks will reinforce one another’s phenotypes.

As mentioned above, partner proteins specific to SYN include lipoprotein-associated, phospholipase A2, vesicle-associated membrane protein 2 (VAMP2) and synaptosomal-associated protein 25 (SNAP25) (in the SNARE complex) and the dopamine transporter^37–39^, whilst partner proteins specific to TPPP include LIM kinase 1^36,40^. In the Sherpa hypothesis, these proteins could be considered as candidates represented by Elements-111 to −170, the addresses of which are in the fields of the PPDP-element. Partners common to SYN and TPPP include tubulin/microtubule, HDAC6/SIRT2, GAPDH and DJ-1^36,41–44^. Since these proteins may undergo post-translational modifications, it is conceivable that differences in modification would determine differences in their roles in the SYN and TPPP networks.

**Fig. 6:**
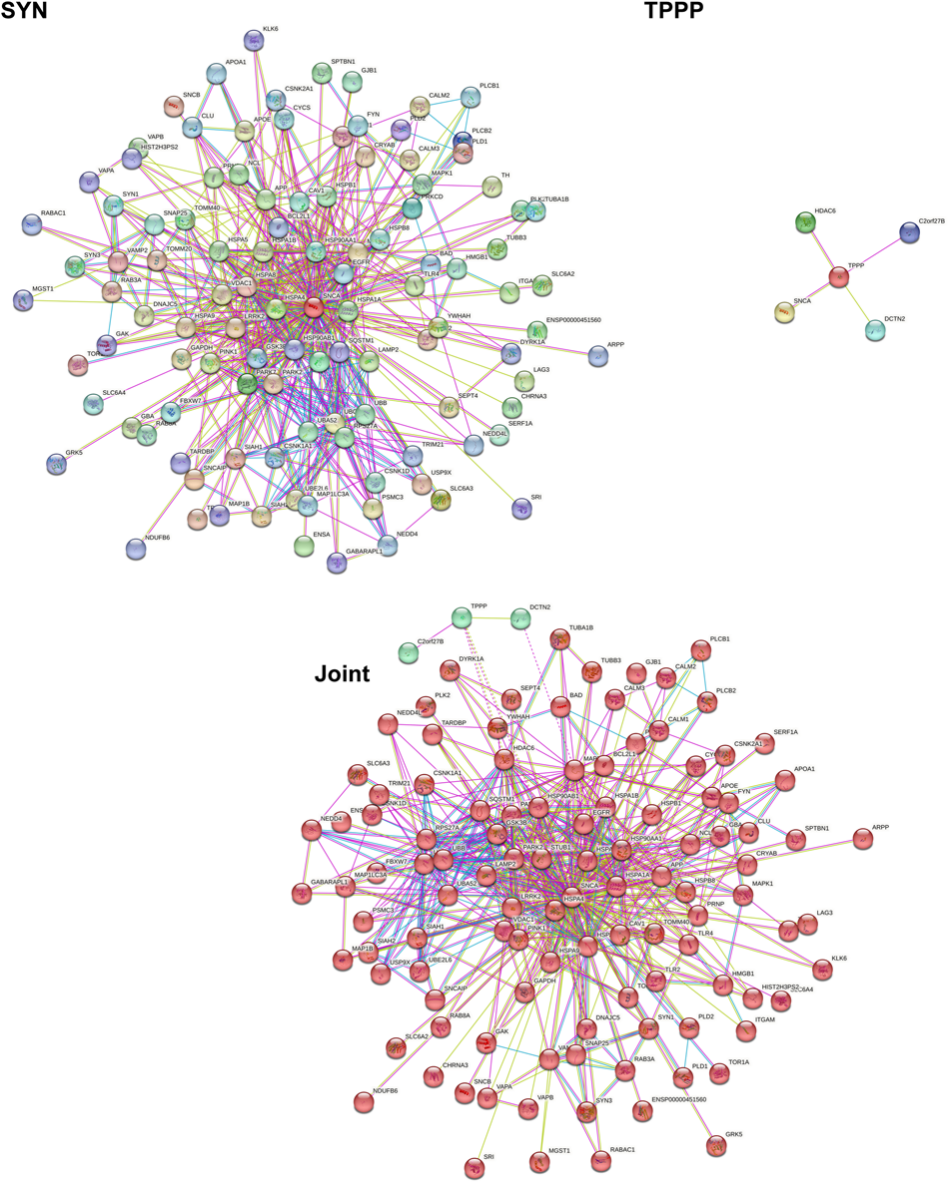
STRING-generated physical subnetworks (the edges indicate that the proteins are part of a physical complex) for SYN, TPPP and SYN-TPPP. Statistical parameters for these three physical subnetworks: SYN: 110 nodes, 522 edges, average node degree 9.49, average local clustering coefficient 0.707; TPPP: 5 nodes, 5 edges, average node degree 1.6, average local clustering coefficient 0.8; SYN-TPPP: 113 nodes, 548 edges, average node degree 9.7, average local clustering coefficient 0.689. In the physical subnetwork for SYN-TPPP, nodes are coloured based on the clustering using the Markov Cluster Algorithm (MCL) option of STRING^73^. The network was clustered to the MCL inflation parameter of 2. “Red” cluster includes 110 nodes that correspond to the proteins physically interacting with SYN, whereas three members of the “green” cluster corresponds to the members of the TPPP physical complex.

In order to obtain additional information pertaining to the actual physical interactions of SYN, TPPP, and SYN-TPPP with their partners (Fig. 6), a network type “physical subnetwork” was selected from the STRING basic settings in all three networks (SYN-, TPPP-, and SYN-TPPP-centred). The joint network of these two PPDPs (SYN and TPPP) show two clusters with clear separation of a very large SYN-centred cluster from the small TPPP-based cluster containing just three proteins. It is important to remember that this analysis does not permit differentiation between the “date” (where interactions between different partners take place at different locations and times) and “party” (where most of the interactions occur simultaneously) type of protein-protein interactions. Analysis of the PPI network shown in Fig. 6 indicated that in addition to binding to SYN, TPPP also interacts with another member of the SYN-based physical subnetwork, HDAC6. Furthermore, dynactin subunit 2 (DCTN2) from the TPPP physical subnetwork interacts with MAPT that also belongs to the SYN-based physical subnetwork. Note that both HDAC6 and MAPT are highly connected hubs, interacting with 23 and 31 proteins, respectively. Finally, according to the Human Protein Atlas^45^, the physical subnetwork of SYN in neurons alone contains 78 nodes (as against 110 in all cells); these nodes include both proteins expressed in all cells (such as Heat shock protein family A member 1 A (HSPA1A) and GAPDH) and proteins enriched in neurons (such as SNAP25, Synapsin-1 (SYN1) and VAMP2). Similarly, the physical network of TPPP in OLGs alone contains 3 nodes (as against 5 nodes in all cells), namely, TPPP, HDAC6 and DCTN2.

## Discussion

The basic idea of the Sherpa hypothesis is that the IDPs corresponding to PPDPs can contribute to the preservation of the cellular phenotype by counteracting perturbations and influencing evolutionary processes. Potential perturbations include the presence or absence of proteins and ligands that interact with the PPDP, along with post-translational modifications and mutations; these perturbations can affect the properties of the PPDP, such as its association with itself and its partners, and hence the preservation of the phenotype. For example, the presence of SYN perturbs the physiological association of TPPP with the microtubule network as does N- and C-terminal truncation of TPPP;^46^ in the case of SYN, post-translational modifications and mutations of SYN can affect its aggregation^47,48^ whilst certain mutations (A53T, A53E, and G51D) can result in an aggressive pathological phenotype^49^.

A significant fraction of proteins do not have a well-defined 3D structure, and these IDPs with their extensive interaction potency display multiple physiological and pathological functions; they often serve as hub proteins^50^. IDPs such as SYN and TPPP characteristically display a remarkable conformational flexibility and structural plasticity. These chameleon proteins are considered to be unstable and are targets of proteolytic degradation, at least, in their nascent forms. However, they readily develop multiple interactions with ligands and macromolecules that can prevent their proteolytic degradation^51^. Their associations with their partner proteins, which may have special functions, are predominantly determined by the intracellular milieu (which itself depends on the cell type).

Molecular chaperones can buffer the destabilizing mutations in individual proteins by providing robustness during proteome evolution. According to Pechmann and Frydman^52^, the IDPs, including the hub proteins (which interact with multiple partners in networks), exhibit more non-conservative substitutions at the expense of enhanced chaperone assistance, thereby highlighting an intricate interplay of molecular chaperones and protein disorder in the course of the network evolution. Mutations were ranked according to their predicted effect on protein stability, and those that were highly destabilizing in more than 20% of their occurrences were considered non-conservative. This interplay balances the possible cost of mutations on protein folding/stability and the benefit of new interactions/functions^52^. In fact, there are molecular chaperones that specialize in folding unfolded/misfolded proteins in order to counteract destabilization and so prevent the disruption of multiple cellular processes. While unique biological functions of proteins often require unique 3D-structures, the IDPs and proteins that contain both IDRs and ordered regions are also functional, being able to engage in biological activities and perform seemingly impossible tasks such as the phenotype stabilization proposed in the Sherpa hypothesis.

The idea that one function of the IDPs, or at least of the PPDPs, is to preserve the phenotype via their stabilizing properties is the key element of the Sherpa hypothesis as illustrated in Fig. 1. The importance of the unfolded/misfolded proteins with non-conservative mutations in the evolution of protein networks has been demonstrated at the proteome level^52^. Therefore, the opportunity for the preservation of the phenotype of the cells by PPDPs is reasonable, especially if they are associated with partners at the very moment of their expression. The ongoing complexation of these proteins by the PPDPs is modelled in the *Coco* program by the many interactions possible for the PPDP elements and by the continued presence of the PPDPs (via the assumption of a zero Downtime i.e., complete stability) for the PPDP elements themselves. The results show that, in this sort of learning program, the associations of PPDPs with their specific partners could constrain the space of new phenotypes explored during evolution. It should be noted that the Sherpa hypothesis and its simulation by *Coco* are not at the level of molecular mechanisms where compartmentalisation, phase separation, mutations, specific concentrations, post-translational modifications etc. should be taken into account individually.

Different basins of the energy landscapes correspond to different phenotypes such as neurons and OLGs or their progenitor and differentiated forms, which depend on the presence of TPPP and/or SYN^33,53–55^. The “depth” and number of the basins determine the outcome of perturbations. If the local minimum is too deep, the phenotype is stable and no transformation occurs into other phenotypes in the course of the evolution; however, at a modest local minimum, the stability can be reduced. The local minima characteristic of neurons and differentiated OLGs may, of course, differ. One can argue that the depth of basins for these phenotypes is strongly determined by the expression of – or the transformation by – the two PPDP proteins, that is, by their intracellular concentrations. In particular, it is reasonable to suppose that the initial concentration of a PPDP is an important factor in these basins (i.e., in the differentiation of a cell and in the maintenance of its differentiated state). In physiological conditions, where the total concentrations of SYN and TPPP in the brain and cerebrospinal fluid are similar^56,57^, the TPPP in neurons and the SYN in OLGs are below the detection level whilst, in pathological conditions, SYN and TPPP are co-enriched and co-colocalized in both neurons and OLGs. In other words, the differentiated state is stable if the second PPDP is undetectable.

**Fig. 7:**
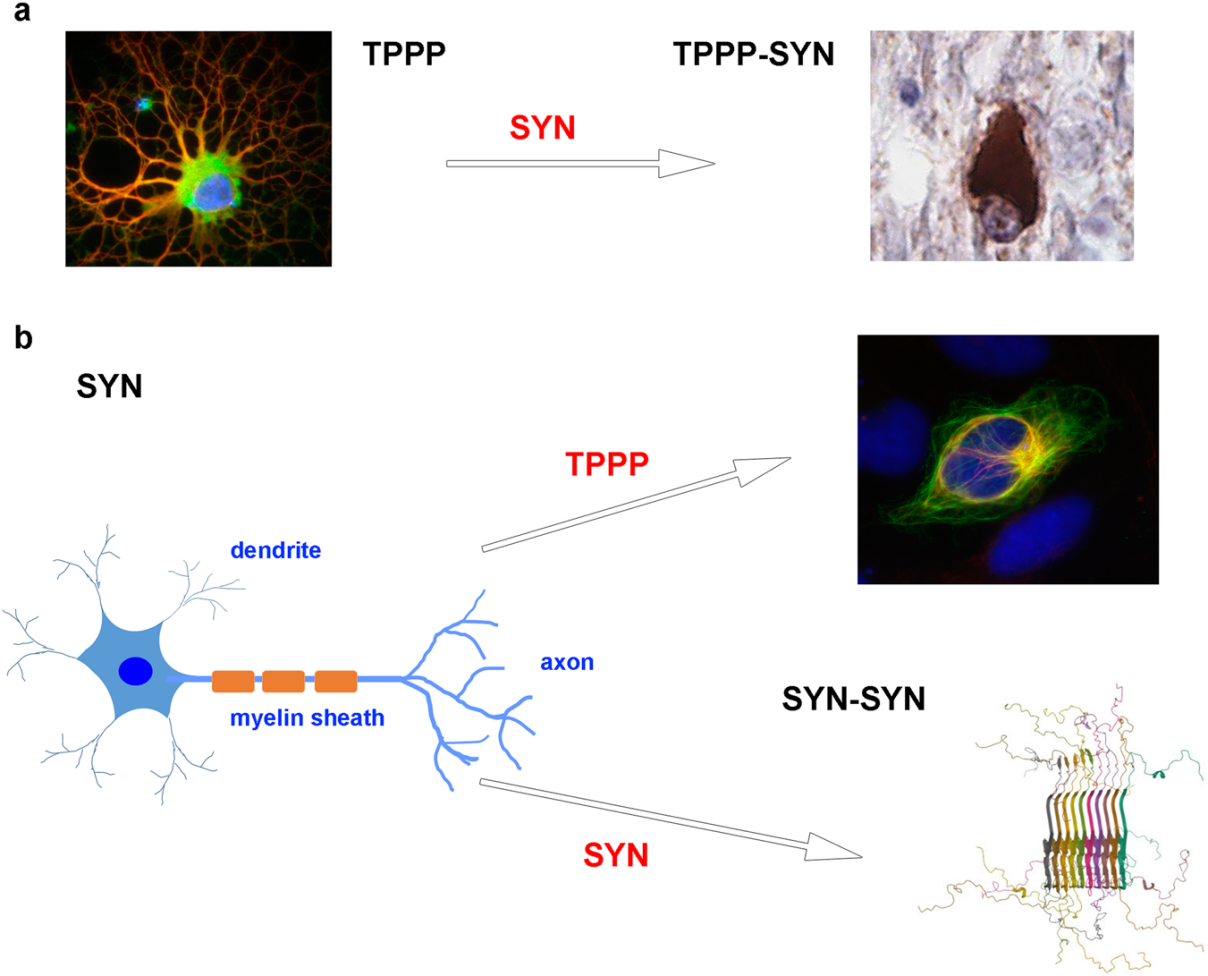
A phenotypic alteration caused by IDPs leads to Parkinsonism. **a** TPPP-derived differentiated OLG proceeds to aggregation by SYN leading to the formation of intracellular inclusion found in multiple system atrophy^33,74^. **b** TPPP and/or SYN-induced aggregation of SYN in neuron; SYN-TPPP assembly-promoted Lewy body characteristic of Parkinson’s disease^75^. Atomic-resolution structure of alpha-synuclein fibrils (PDB ID: 2N0A)^76^.

A model system has been established with SYN and TPPP, which are expressed endogenously in neurons and OLGs, respectively, in normal human brain (Fig. 7)^33,53–55^. The *Coco* program simulates certain related experimental results, namely, the promotion of phenotype alterations due to the hetero-association of the disordered TPPP with another disordered protein, SYN, which occur because of the transmission of SYN into OLGs or of TPPP into neuronal cells^17,58–62^. In addition, the pathological overexpression of SYN could be considered as acting as a second PPDP resulting in SYN assembly with phenotypic alteration as illustrated by the *Coco* program. Within the aggregates that ultimately result from SYN/TPPP interactions, the partner proteins lose their functions^63^, a situation that corresponds to the perturbations predicted by the Sherpa hypothesis and to the results of our simulation. These perturbations lead to neurodegenerative disorders or other diseases^17,47,64–67^.

In the case of these PPDPs, the therapeutic approaches opened up by the Sherpa hypothesis include those that limit perturbations, which would help maintain the functioning of the PPDP and its partners. Another approach is the construction/selection of novel, highly specific, synthetic or designed PPDP helpers (that could be activators or inhibitors) out of a set of oligonucleotide or peptide aptamers; each individual aptamer would target the PPDP or, possibly, the partners of the PPDP. Such aptamers could be linked to one another to form a single construct for the modulation of both the homologous and the heterologous associations of PPDPs within the cells. These constructs would activate or inactivate the physiological and pathological proteins as appropriate, which might well suffice to avoid the pathological consequences of the association of SYN-SYN and SYN-TPPP; consequently, such synthetic PPDP helpers could act potentially as therapeutic agents. Encouragingly, recent studies have revealed that both oligonucleotide- and peptide-based aptamers, modulators of protein-protein interactions, can effectively counteract the pathological aggregations of these PPDPs^46,68–72^.

Finally, the associations of a PPDP with its partner proteins may help other types of differentiated cells resist perturbations. In the case of TPPP, its stabilization of the microtubule network is central to differentiation; this stabilization is disrupted by the pathological presence of SYN. The hypothesis may therefore even prove helpful in understanding and treating diseases like Parkinson’s disease and multiple system atrophy in which the interactions between SYN and TPPP are implicated^15–17,36,62^.

## Methods

### *Coco* program

In this version of *Coco*, a thousand elements are connected initially with one another in a pattern that has some structure. In essence, connections were made at random between the 36 elements in a group of elements (e.g., between elements with addresses 50 to 86) for each of nine groups (with addresses 150 to 186, 250 to 286, 350 to 386 etc.). Three of the thousand elements are chosen to be input elements (with addresses 1, 2 and 3), and three are chosen to be output elements (998, 999 and 1000). *Coco* learns to respond to repeated cycles of an input sequence of (1, 2, 3, 2, 3) with an output sequence of (1000, 999, 999, 1000, 998). Most elements have a *Now* and a *Next* field that can vary in size from containing the address of just one element to containing the addresses of up to 8 different elements per field (N.B., the address of a single element can occur many times in a field). The size of an element’s fields increases if this element is part of a successful response (i.e., is part of the state of an *Active* subset that is rewarded) and decreases if it is part of an unsuccessful one (i.e., is part of a state that is punished). The contents of these fields also change with learning, such that the number of times an element’s address is present in the fields of another element increases if the elements are both part of a rewarded state or sequence of states of the *Active* subset and, reciprocally, decreases in the case of punishment. In the rewarding and punishing process, an input (which corresponds to an address of elements-1 or −2 or −3) being inserted into a state of the *Active* subset) must be followed by an output (corresponding to 998, 999 or 1000) appearing in the same of in one of the following two states of the *Active* subset. If an output does not appear, *Coco* forces a randomly chosen address of an output into the last of the three states of the *Active* subset, thereby allowing an appropriate rewarding or punishing. Downtime is an important characteristic of the elements in *Coco*. It corresponds to the time needed for the resynthesis of a regulatory molecule following the degradation of this molecule when its function is no longer needed. Once an element has been part of a state of the *Active* subset, it cannot be part of a new state until there have been 12 more states of the *Active* subset.

To model a stable, highly connected PPDP like SYN and TPPP in *Coco*, we choose the element with address 10 to represent the PPDP; in other words, 10 identifies the element. We give Element-10 a Downtime of zero and a high strength via its *Next* connections so that it can be present in consecutive states of the *Active* subset (corresponding to a stable protein). We also give Element-10 large *Now* and *Next* fields of fixed sizes that can each contain 60 addresses (corresponding to the many proteins with which an IDP might interact). These addresses run from 111 to 140 in Element-10’s *Now* field and from 141 to 170 in its *Next* field; each of these addresses occurs twice. It is important to note that the addresses in Element-10’s fields are fixed at the start of the program and cannot be changed – in other words, they are exempt from the rewarding and punishing routines.

To model the effects of a perturbation, we first let *Coco* learn and, when this learning is stable, disable the rewarding and punishing routines along with those responsible for forced inputs from the environment (1, 2 and 3) and for forced outputs. *Coco* then continues with an unchanging cycle of states of the *Active* subset, which we take to represent a cell that has undergone natural selection then remaining in a differentiated state or phenotype (more accurately, a cycle of phenotypes) despite the absence of selective pressure. At this stage, we subject *Coco* to a major perturbation. Firstly, in the time step 999, we double the size of the *Active* subset so that from then on a state of the *Active* subset contains 12 addresses. Secondly, in the next time step where there would otherwise be an input of 1, we create a new state of the *Active* subset; the addresses in this new state are those of elements that do not belong to those that are in the learnt sequence (i.e., they are inactive elements).

To model the effects of the introduction of a second IDP, we use a slightly different version of *Coco*. After the program has learnt, we create a second IDP using the inactive elements. The pattern of connections between the elements that are *active* in one of the last twenty-four states of the *Active* subset is recorded (which more than covers an entire learnt sequence of states). This pattern is then mimicked using only those elements that never have addresses in the *Active* subset (they therefore have Downtimes of zero); one of these elements has a similar pattern of connections to the first IDP (Element-10) and is designated as the second IDP. This IDP is given the same properties as the first IDP with a Downtime of zero and a *Next* connectivity score that ensures it is always in a state of the *Active* subset. The second IDP is introduced into a state of the *Active* subset along with some of the elements to which this second IDP is connected. *Coco* then continues as before.

Finally, we use the modified version of *Coco* to investigate whether the removal of the second IDP by putting its *Next* connectivity score to zero can lead to the restoration of the original, learnt sequence of the states of the *Active* subset.

Availability of computer code and algorithm: The code used to generate some of the results and an overview of the programs are given in Supplementary Information.

### Analysis of interactability of SYN and TPPP

Interactability of human SYN (UniProt ID: P37840) and TPPP (UniProt ID: O94811) was analysed by the online database resource Search Tool for the Retrieval of Interacting Genes (STRING)^73^. STRING generates a network of predicted associations based on predicted and experimentally-validated information on the interaction partners of a protein of interest^73^. In the corresponding network, the nodes correspond to proteins, whereas the edges show predicted or known functional associations. Seven types of evidence are used to build the corresponding network, and are indicated by the differently coloured lines: a green line represents neighbourhood evidence; a red line – the presence of fusion evidence; a purple line – experimental evidence; a blue line – co-occurrence evidence; a light blue line – database evidence; a yellow line – text mining evidence; and a black line – co-expression evidence^73^.

In this study, STRING was used in two different modes: to create PPI networks centred on human SYN or TPPP and to produce a joint SYN-TPPP PPI network. Individual protein-centred PPI networks and a joint SYN-TPPP-centred network were generated by STRING (https://string-db.org/) using a custom value of 500 first-shell interactions and custom confidence level of 0.45. The resulting PPI networks were further analysed using STRING-embedded routines in order to retrieve the network-related statistics, such as: the number of nodes (proteins); the number of edges (interactions); average node degree (average number of interactions per protein); average local clustering coefficient (which defines how close the neighbours are to being a complete clique – if a local clustering coefficient is equal to 1, then every neighbour connected to a given node N_i_ is also connected to every other node within the neighbourhood, and if it is equal to 0, then no node that is connected to a given node N_i_ connects to any other node that is connected to N_i_); expected number of edges (which is a number of interactions among the proteins in a random set of proteins of similar size); and a PPI enrichment p-value (which is a reflection of the fact that query proteins in the analysed PPI network have more interactions among themselves than what would be expected for a random set of proteins of similar size, drawn from the genome. It was pointed out that such an enrichment indicates that the proteins are at least partially biologically connected, as a group). We also looked for the actual physical interactions of SYN, TPPP, and SYN-TPPP with their partners. To this end, a network type “physical subnetwork” was selected from the STRING basic settings in all three networks (SYN-, TPPP-, and SYN-TPPP-centred). Furthermore, we clustered the physical SYN-TPPP subnetwork using the Markov Cluster Algorithm (MCL) option of STRING, with the network being clustered to the MCL inflation parameter of 2.

### Reporting summary

Further information on research design is available in the Nature Research Reporting Summary linked to this article.

## Supplementary information

Supplementary Information

Reporting summary

Supplementary Table 1

Supplementary Table 2

Supplementary Table 3

## Data Availability

All data generated or analysed during this study are included in this published article (and its Supplementary Information files).

## References

[1] Wright PE, Dyson HJ (2015). Intrinsically disordered proteins in cellular signalling and regulation. Nat. Rev..

[2] Oldfield CJ (2005). Comparing and combining predictors of mostly disordered proteins. Biochemistry.

[3] Peng Y (2019). A Metastable Contact and Structural Disorder in the Estrogen Receptor Transactivation Domain. Structure.

[4] Suryadinata R, Sadowski M, Sarcevic B (2010). Control of cell cycle progression by phosphorylation of cyclin-dependent kinase (CDK) substrates. Biosci. Rep..

[5] Uversky VN, Kuznetsova IM, Turoverov KK, Zaslavsky B (2015). Intrinsically disordered proteins as crucial constituents of cellular aqueous two phase systems and coacervates. FEBS Lett..

[6] Banani SF, Lee HO, Hyman AA, Rosen MK (2017). Biomolecular condensates: organizers of cellular biochemistry. Nat. Rev..

[7] Dunker AK, Cortese MS, Romero P, Iakoucheva LM, Uversky VN (2005). Flexible nets. The roles of intrinsic disorder in protein interaction networks. FEBS J..

[8] Uversky VN, Oldfield CJ, Dunker AK (2005). Showing your ID: intrinsic disorder as an ID for recognition, regulation and cell signaling. J. Mol. Recognit..

[9] Sharma R, Raduly Z, Miskei M, Fuxreiter M (2015). Fuzzy complexes: Specific binding without complete folding. FEBS Lett..

[10] Uversky VN (2011). Multitude of binding modes attainable by intrinsically disordered proteins: a portrait gallery of disorder-based complexes. Chem. Soc. Rev..

[11] Uversky VN (2008). Amyloidogenesis of natively unfolded proteins. Curr. Alzheimer Res..

[12] Uversky VN (2010). Targeting intrinsically disordered proteins in neurodegenerative and protein dysfunction diseases: another illustration of the D(2) concept. Expert Rev. Proteom..

[13] Uversky VN, Oldfield CJ, Dunker AK (2008). Intrinsically disordered proteins in human diseases: introducing the D2 concept. Annu. Rev. Biophys..

[14] Hollstein M, Sidransky D, Vogelstein B, Harris CC (1991). p53 mutations in human cancers. Science (New York, N.Y.).

[15] Spillantini MG (1997). Alpha-synuclein in Lewy bodies. Nature.

[16] Baba M (1998). Aggregation of alpha-synuclein in Lewy bodies of sporadic Parkinson’s disease and dementia with Lewy bodies. Am. J. Pathol..

[17] Kovacs GG (2004). Natively unfolded tubulin polymerization promoting protein TPPP/p25 is a common marker of alpha-synucleinopathies. Neurobiol. Dis..

[18] Irvine GB, El-Agnaf OM, Shankar GM, Walsh DM (2008). Protein aggregation in the brain: the molecular basis for Alzheimer’s and Parkinson’s diseases. Mol. Med. (Cambridge, Mass).

[19] Trejo-Lopez JA, Yachnis AT, Prokop S (2022). Neuropathology of Alzheimer’s Disease. Neurotherapeutics.

[20] Mochizuki H, Choong CJ, Masliah E (2018). A refined concept: alpha-synuclein dysregulation disease. Neurochem. Int..

[21] Liu Z, Huang Y (2014). Advantages of proteins being disordered. Protein Sci..

[22] Beveridge R, Calabrese AN (2021). Structural Proteomics Methods to Interrogate the Conformations and Dynamics of Intrinsically Disordered Proteins. Front. Chem..

[23] Uversky VN (2016). Dancing Protein Clouds: The Strange Biology and Chaotic Physics of Intrinsically Disordered Proteins. J. Biol. Chem..

[24] Uversky VN (2016). p53 Proteoforms and Intrinsic Disorder: An Illustration of the Protein Structure-Function Continuum Concept. Int. J. Mol. Sci..

[25] Uversky VN (2019). Protein intrinsic disorder and structure-function continuum. Prog. Mol. Biol. Transl. Sci..

[26] Uversky VN (2017). Looking at the recent advances in understanding alpha-synuclein and its aggregation through the proteoform prism. F1000Research.

[27] Kauffman, S. *At home in the Universe, the search for the laws of complexity*. (Penguin, London); (1996).

[28] Norris V, Engel M, Demarty M (2012). Modelling biological systems with competitive coherence. Adv. Artif. Neural Syst..

[29] Norris V (2021). Competitive Coherence Generates Qualia in Bacteria and Other Living Systems. Biology.

[30] Vohradsky J, Ramsden JJ (2001). Genome resource utilization during prokaryotic development. FASEB J..

[31] Cheng F, Vivacqua G, Yu S (2011). The role of alpha-synuclein in neurotransmission and synaptic plasticity. J. Chem. Neuroanat..

[32] Longhena F, Faustini G, Spillantini MG, Bellucci A (2019). Living in Promiscuity: The Multiple Partners of Alpha-Synuclein at the Synapse in Physiology and Pathology. Int. J. Mol. Sci..

[33] Lehotzky A (2010). Tubulin polymerization-promoting protein (TPPP/p25) is critical for oligodendrocyte differentiation. Glia.

[34] Fu MM (2019). The Golgi Outpost Protein TPPP Nucleates Microtubules and Is Critical for Myelination. Cell.

[35] Hisahara S (2021). SIRT1 decelerates morphological processing of oligodendrocyte cell lines and regulates the expression of cytoskeleton-related oligodendrocyte proteins. Biochem Biophys. Res Commun..

[36] Olah J (2020). Microtubule-Associated Proteins with Regulatory Functions by Day and Pathological Potency at Night. Cells.

[37] Sun J (2019). Functional cooperation of alpha-synuclein and VAMP2 in synaptic vesicle recycling. Proc. Natl Acad. Sci. USA.

[38] Gao V, Briano JA, Komer LE, Burre J (2023). Functional and Pathological Effects of alpha-Synuclein on Synaptic SNARE Complexes. J. Mol. Biol..

[39] Pingale TD, Gupta GL (2021). Novel therapeutic approaches for Parkinson’s disease by targeting brain cholesterol homeostasis. J. Pharm. Pharmacol..

[40] Acevedo K (2007). The phosphorylation of p25/TPPP by LIM kinase 1 inhibits its ability to assemble microtubules. Exp. cell Res..

[41] Cartelli D (2016). alpha-Synuclein is a Novel Microtubule Dynamase. Sci. Rep..

[42] de Oliveira RM (2017). The mechanism of sirtuin 2-mediated exacerbation of alpha-synuclein toxicity in models of Parkinson disease. PLoS Biol..

[43] Kumar R (2019). Partially oxidized DJ-1 inhibits alpha-synuclein nucleation and remodels mature alpha-synuclein fibrils in vitro. Commun. Biol..

[44] Olah J, Lehotzky A, Szenasi T, Ovadi J (2021). Anti-Aggregative Effect of the Antioxidant DJ-1 on the TPPP/p25-Derived Pathological Associations of Alpha-Synuclein. Cells.

[45] The Human Protein Atlas. (2023). https://www.proteinatlas.org/.

[46] Tokesi N (2014). Identification of motives mediating alternative functions of the neomorphic moonlighting TPPP/p25. Biochimica et. biophysica acta.

[47] Kalia LV, Lang AE (2015). Parkinson’s disease. Lancet (Lond., Engl.).

[48] Schmid AW, Fauvet B, Moniatte M, Lashuel HA (2013). Alpha-synuclein post-translational modifications as potential biomarkers for Parkinson disease and other synucleinopathies. Mol. Cell Proteom..

[49] Whittaker HT, Qui Y, Bettencourt C, Houlden H (2017). Multiple system atrophy: genetic risks and alpha-synuclein mutations. F1000Research.

[50] Hu G, Wu Z, Uversky VN, Kurgan L (2017). Functional Analysis of Human Hub Proteins and Their Interactors Involved in the Intrinsic Disorder-Enriched Interactions. Int. J. Mol. Sci..

[51] Lehotzky A (2021). Co-Transmission of Alpha-Synuclein and TPPP/p25 Inhibits Their Proteolytic Degradation in Human Cell Models. Front. Mol. Biosci..

[52] Pechmann S, Frydman J (2014). Interplay between chaperones and protein disorder promotes the evolution of protein networks. PLoS Comput. Biol..

[53] Maroteaux L, Campanelli JT, Scheller RH (1988). Synuclein: a neuron-specific protein localized to the nucleus and presynaptic nerve terminal. J. Neurosci..

[54] Takahashi M (1993). A brain-specific protein p25 is localized and associated with oligodendrocytes, neuropil, and fiber-like structures of the CA3 hippocampal region in the rat brain. J. Neurochem..

[55] Bates CA, Zheng W (2014). Brain disposition of alpha-Synuclein: roles of brain barrier systems and implications for Parkinson’s disease. Fluids barriers CNS.

[56] Vincze O (2011). A new myelin protein, TPPP/p25, reduced in demyelinated lesions is enriched in cerebrospinal fluid of multiple sclerosis. Biochem Biophys. Res Commun..

[57] Parnetti L (2019). Parkinson’s and Lewy body dementia CSF biomarkers. Clin. Chim. acta; Int. J. Clin. Chem..

[58] Wakabayashi K, Yoshimoto M, Tsuji S, Takahashi H (1998). Alpha-synuclein immunoreactivity in glial cytoplasmic inclusions in multiple system atrophy. Neurosci. Lett..

[59] Ota K (2014). Relocation of p25alpha/tubulin polymerization promoting protein from the nucleus to the perinuclear cytoplasm in the oligodendroglia of sporadic and COQ2 mutant multiple system atrophy. Acta neuropathologica Commun..

[60] Mavroeidi P (2019). Endogenous oligodendroglial alpha-synuclein and TPPP/p25alpha orchestrate alpha-synuclein pathology in experimental multiple system atrophy models. Acta neuropathologica.

[61] Nishimura Y (2022). Early and extensive alterations of glial connexins, distal oligodendrogliopathy type demyelination, and nodal/paranodal pathology are characteristic of multiple system atrophy. Brain Pathol. (Zur., Switz.).

[62] Surguchov A, Surguchev A (2022). Synucleins: New Data on Misfolding, Aggregation and Role in Diseases. Biomedicines.

[63] Wakabayashi K (2013). The Lewy body in Parkinson’s disease and related neurodegenerative disorders. Mol. Neurobiol..

[64] Preusser M, Lehotzky A, Budka H, Ovadi J, Kovacs GG (2007). TPPP/p25 in brain tumours: expression in non-neoplastic oligodendrocytes but not in oligodendroglioma cells. Acta neuropathologica.

[65] Feng DD, Cai W, Chen X (2015). The associations between Parkinson’s disease and cancer: the plot thickens. Transl. neurodegeneration.

[66] Olah J, Bertrand P, Ovadi J (2017). Role of the microtubule-associated TPPP/p25 in Parkinson’s and related diseases and its therapeutic potential. Expert Rev. Proteom..

[67] Surguchev AA, Emamzadeh FN, Surguchov A (2019). Cell Responses to Extracellular alpha-Synuclein. Molecules (Basel, Switz.).

[68] Szunyogh S, Olah J, Szenasi T, Szabo A, Ovadi J (2015). Targeting the interface of the pathological complex of alpha-synuclein and TPPP/p25. Biochimica et. biophysica acta.

[69] Szenasi T (2017). Challenging drug target for Parkinson’s disease: Pathological complex of the chameleon TPPP/p25 and alpha-synuclein proteins. Biochim Biophys. Acta Mol. Basis Dis..

[70] Zheng Y (2018). Novel DNA Aptamers for Parkinson’s Disease Treatment Inhibit alpha-Synuclein Aggregation and Facilitate its Degradation. Mol. Ther..

[71] Ren X (2019). Exosomal DNA Aptamer Targeting alpha-Synuclein Aggregates Reduced Neuropathological Deficits in a Mouse Parkinson’s Disease Model. Mol. Ther..

[72] Khan I, Preeti K, Fernandes V, Khatri DK, Singh SB (2021). Role of MicroRNAs, Aptamers in Neuroinflammation and Neurodegenerative Disorders. Cell. Mol. Neurobiol..

[73] Szklarczyk D (2023). The STRING database in 2023: protein-protein association networks and functional enrichment analyses for any sequenced genome of interest. Nucleic acids Res..

[74] Orosz F (2004). TPPP/p25: from unfolded protein to misfolding disease: prediction and experiments. Biol. Cell.

[75] Tokesi N (2010). TPPP/p25 promotes tubulin acetylation by inhibiting histone deacetylase 6. J. Biol. Chem..

[76] Tuttle MD (2016). Solid-state NMR structure of a pathogenic fibril of full-length human alpha-synuclein. Nat. Struct. Mol. Biol..

