# Supplementary material for: The Sherpa hypothesis: Phenotype-Preserving Disordered Proteins stabilize the phenotypes of neurons and oligodendrocytes
Source: NPJ Syst Biol Appl. 2023 Jul 11;9:31. doi: 10.1038/s41540-023-00291-8 (PMC10336114; doi:10.1038/s41540-023-00291-8)
Supplement: Supplementary file 1 — Supplementary Information [file 41540_2023_291_MOESM1_ESM.pdf]

The Sherpa hypothesis: Phenotype-Protecting Disordered Proteins stabilize the phenotypes of neurons and oligodendrocytes

Vic Norris,[1]* Judit Oláh,[2] Sergey N. Krylov,[3] Vladimir N. Uversky,[4] and Judit Ovádi[2]

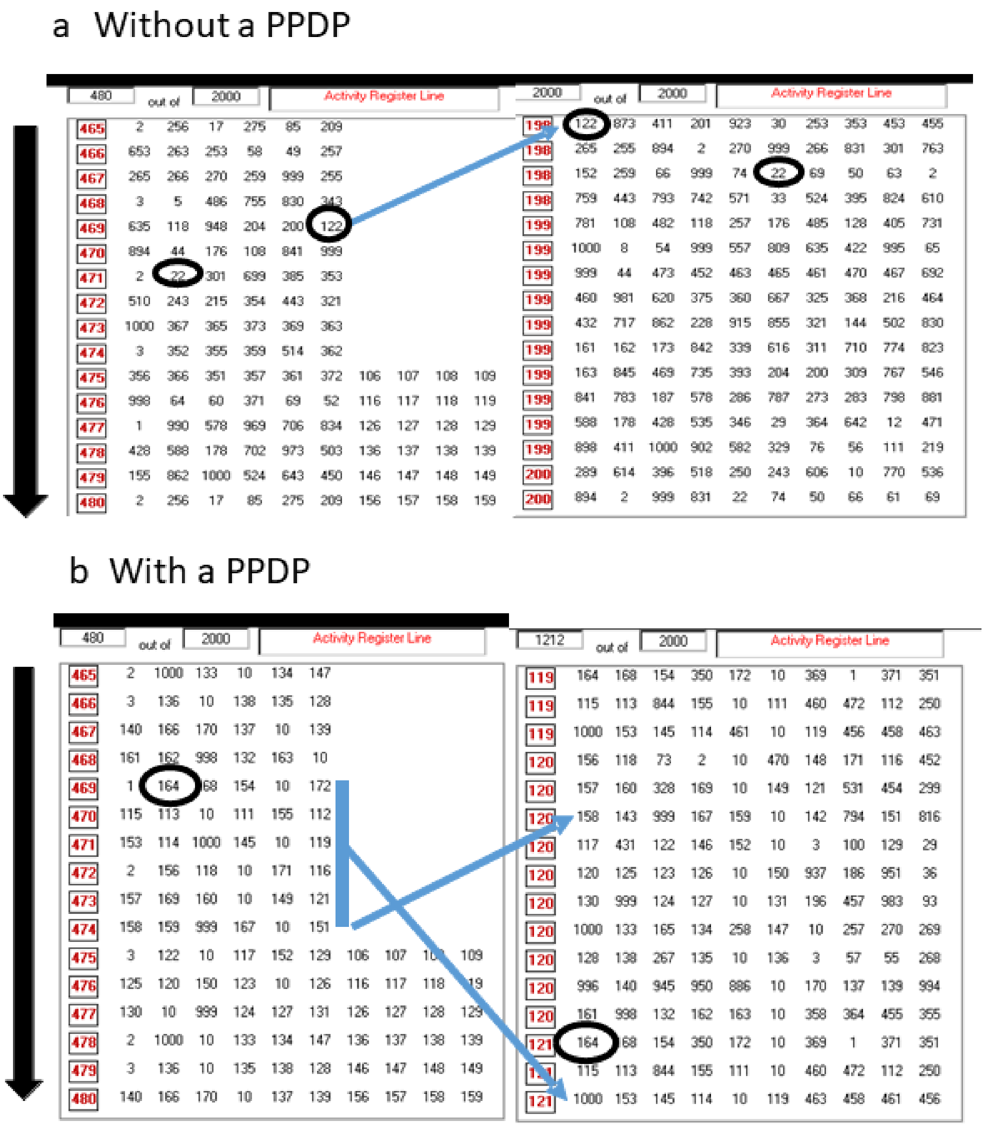

Supplementary Figure 1. A PPDP can protect the phenotype from perturbation.

The learnt sequence of states in the *Active* subset is shown in the left part of the figures and the perturbed state in the right part. a: In the absence of a PPDP element, very few of the *active* elements in the learnt state are preserved; two of their addresses are shown. b: In the presence of a PPDP element (Element-10) the learnt sequence is preserved as indicated for a few states with the blue line and the black ovals. The black arrow represents time. The data were obtained from the CocoIDP825NatComm program.

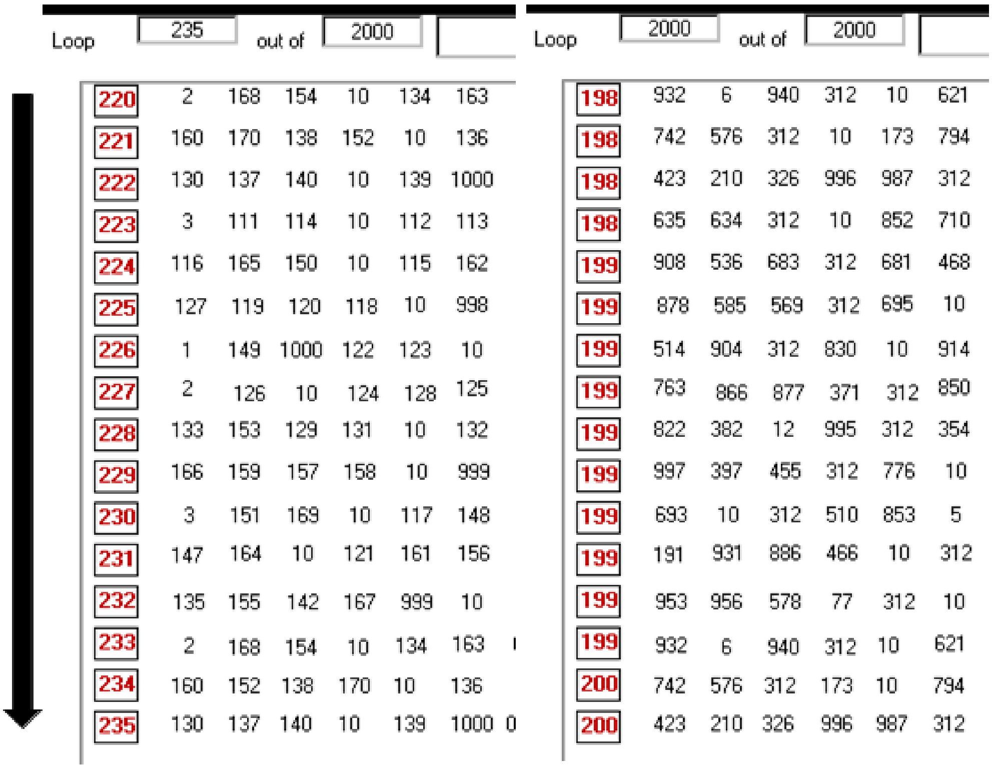

Supplementary Figure 2. Introduction of a second PPDP causes the phenotype to be lost.

The learnt sequence of *Active* states contains only the PPDP Element-10 and the contents of its fields (left panel). This sequence is disrupted by the presence of a second PPDP element (Element-312) (right panel). The black arrow represents time.

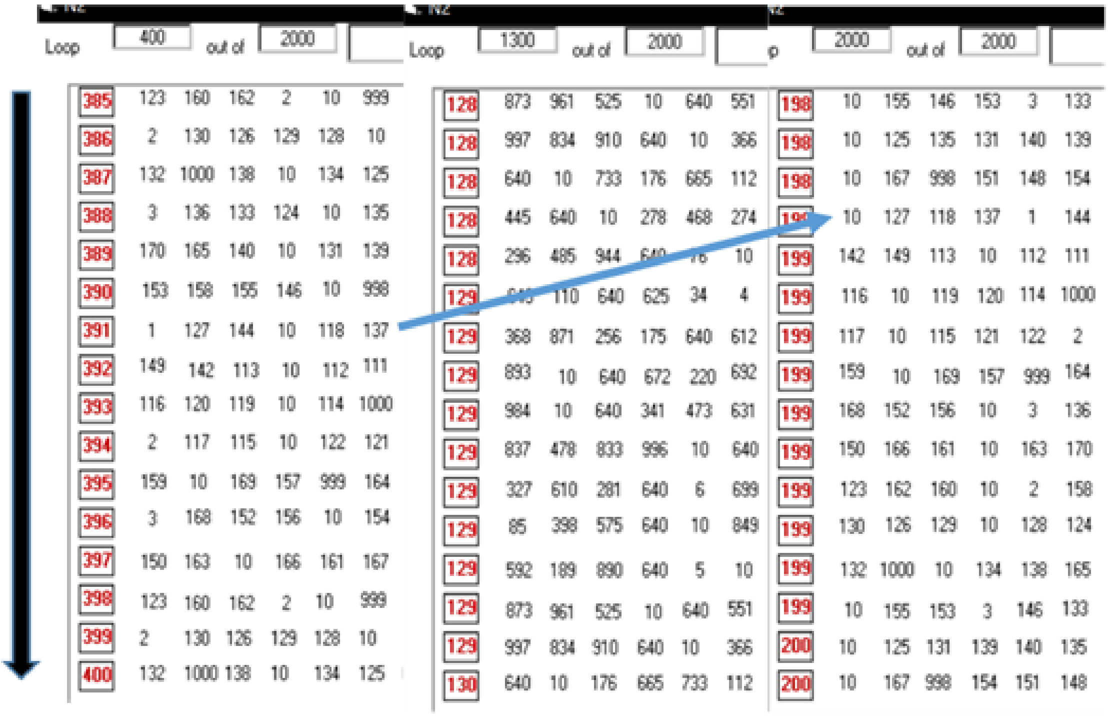

Supplementary Figure 3. Elimination of the second PPDP can allow recovery.

The learnt sequence of *Active* states (left panel) is lost in the presence of a second PPDP element (Element-640) (centre panel); when the second PPDP element is removed and when the order of the learnt sequence is restored by the forced presence of an input, the learnt sequence is largely recovered (right panel). The blue arrow shows the restoration of one of the states in the learnt sequence. The black arrow represents time. The data were obtained from the CocoIDP827NatComm program.

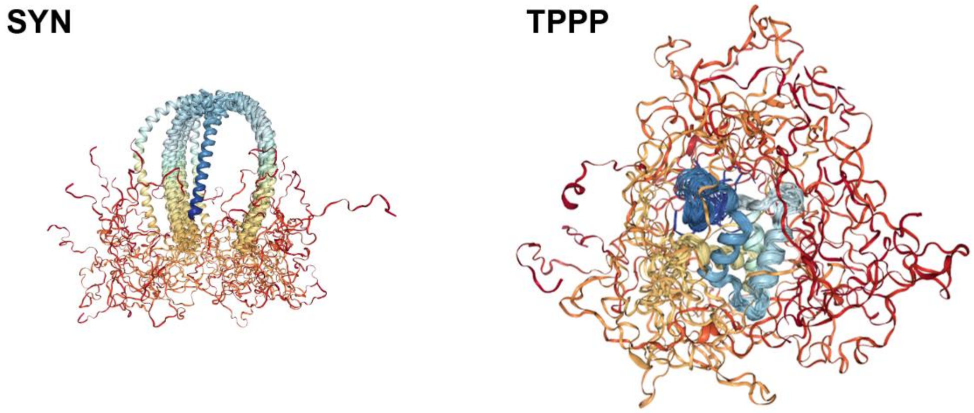

Supplementary Figure 4. Functional intrinsic disorder of SYN and TPPP.

Left panel. NMR solution structure of human SYN bound to a micelle of the detergent sodium lauroyl sarcosinate (SLAS) (PDB ID: 2KKW) [1]. Right panel. A. NMR solution structure of human homologue TPPP (TPPP3/p20) (PDB ID: 2JRF) (https://www.rcsb.org/structure/2JRF), a homologue of TPPP.

REFERENCE

1 Rao, J. N., Jao, C. C., Hegde, B. G., Langen, R. & Ulmer, T. S. A combinatorial NMR and EPR approach for evaluating the structural ensemble of partially folded proteins. *Journal of the American Chemical Society* **132**, 8657-8668, doi:10.1021/ja100646t (2010).

Program overview CocoIDP825NatComm

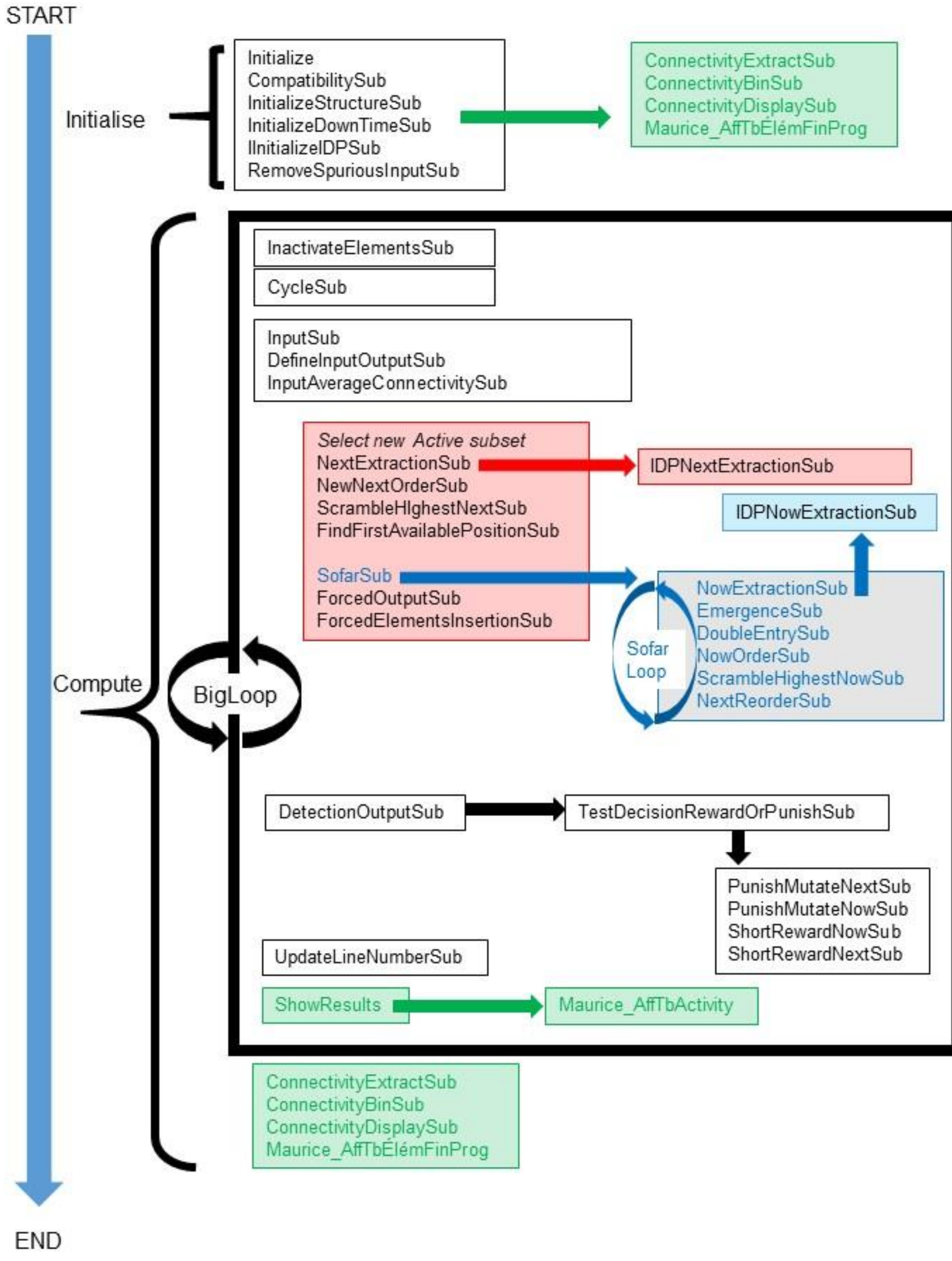

Initialise subroutines

Initialize first calls on CompatibilitySub. It then randomly fills the fields of all elements with addresses of other elements (but avoids putting output addresses into the fields of inputs). It makes 1/10 of these connections negative (which reduces the probability that the two elements will be selected together). It calls on InitializeStructureSub, InitializeDownTimeSub and (if UseIDP = 1) InitializeIDPSub. It calls on RemoveSpuriousInputSub, clears the Activity register, sets the LineNumber and NewLineNumber. It loads the Activity register with random elements (but excluding inputs). It sets up inputs and various counters. Finally, it shows some elements and their contents with Maurice_AffTbÉlémFinProg (ConnectivityExtractSub, ConnectivityBinSub, ConnectivityDisplaySub are not used here).

CompatibilitySub uses a CompatibilityTable(element1, element2) to allow altering the Now and Next scores obtained for each element so as to take into account that some elements should be selected together and others (like inputs) should not. Here this table is set so that EmergenceSub will give compatibility between all elements except for inputs with inputs.

InitializeStructureSub first calls on ConnectivityExtractSub, ConnectivityBinSub, ConnectivityDisplaySub, Maurice_AffTbÉlémFinProg. Then starting at element-51, it takes elements-51 and writes the randomly chosen addresses of elements-50 to -74 into each position in the Now field of element-51; it then repeats this for element-52 and so on until element-86. With a step size of 100, it repeats this sequence starting with element-151 and writing into its field addresses of elements-150 to -174 etc.

InitializeDowntimeSub gives every element a Downtime of 12 because DowntimeNumberRange is set to 0

InitializeIDPSub gives the IDP element-10 Now and Next fields of max size (1 to 60 for Now and 61 to 120 for Next)' these fields are filled with addresses 111, 111, 112, 112, 113, 113 …170, 170 (each address is added twice) so the Now range is 111 to 140 and the Next range is 141 to 170 Then 1/10 of all the elements (excluding inputs and outputs) have the IDP address written once into their Now and Next fields

RemoveSpuriousInputSub prevents input addresses being generated in the Active subset that are not coming from the environment. It goes through the fields and removes input addresses. The effect of leaving allowing 'false' inputs to be generated may be worth exploring.

Maurice_AffTbÉlémFinProg is called on by several subroutines to show the first 35 elements and the contents of their fields.

Compute subroutines

InactivateElementsSub reduces the Downtime of each element by 1; then it gives the elements in the Active subset the maximum Downtime of 12. The outputs and the IDP are given Downtimes of 0

CycleSub If CyclePermission=1 (which is NOT the case here), Coco can insert the address of a new element (the CycleElement) every four lines of the Activity Register' this address is increased by 1 every time it is inserted so, in principle, it allows all the elements to be selected.

InputSub uses a counter, InputTotal, to choose the input by calling DefineInputOutputSub and then on InputAverageConnectivitySub

DefineInputOutputSub defines the 5 conditions selected by InputTotal: InputA(1), InputA(2), and In-

putA(3) give inputs of 1, 2 and 3, respectively, if they are set to 1. OutputA(1), OutputA(2) and OutputA(3) which, if set to 1, require outputs of Enumber-0 (i.e., 1000), Enumber-1, and Enumber-2, respectively as outputs.

InputAverageConnectivitySub gives InputNowScore() and InputNextScore() high scores so that they are forced into the new line of the Activity Register. However, after LoopsTillStorm-200 (i.e., after Loop 800), the inputs are no longer forced into the Activity Register because their scores are set to zero here.

NextExtractionSub extracts how often an address has been referred to in the Next field of the elements in the Activity Register; this gives the score for NextScoreRegister(element address). Outputs are not scored. Signs are taken into account in increasing or decreasing the score. If UseIDP=1 and the element in the Activity Register is the IDP, the routine calls on IDPNextExtractionSub.

IDPNextExtractionSub extracts how often an address is present in the Next field of the IDP element; this gives a score to be added to the NextScoreRegister(element address). Signs are taken into account in increasing or decreasing the score. It gives the IDP a NextScoreRegister(IDP) score of IDPSparing (set to 5).

NewNextOrderSub puts EmergentNextScoreRegister() = NextScoreRegister() (because the EmergenceSub is largely unused) and then it crudely ranks the scores in the NextScoreRegister() as HighestNext(top,1) = address and HighestNext(top,2) = its score, then HighestNext(second to top,1) = its address and HighestNext(second to top,2) = its score, etc.

ScrambleHighestNextSub prevents an artefact which could occur with supposedly independent runs of the program in which the same Nexts could be selected each run despite there being other Nexts with the same score (this is due to NewNextOrderSub puts the lowest addresses on top (e.g. if element-25 and element-37 have the same score, element-25 would always be selected). This routine therefore scrambles the order of elements with the same score.

FindFirstAvailablePositionSub looks at the new line of the Activity Register and, if a position is unfilled, it fills it with the HighestNext. It then updates the HighestNexts (to avoid reselecting the same element), and sets Sofar to point to the last filled position in the new line.

Compute: SofarSub

SofarSub First, it ensures that a second input cannot be loaded. It then calls on NowExtractionSub (which obtains the Now scores of the elements in the new line of the Activity Register). It could – but does not here – introduce noise. It calls on EmergenceSub (which converts the NowScoreRegister() scores into EmergentNowScoreRegister() scores), on DoubleEntrySub (which prevents an element being selected twice) and on NowOrderSub (which uses the EmergentNowScore() scores to order them into the HighestNow scores). It uses ScrambleHighestNowSub to prevent an artefact. It then selects the element from the top of the HighestNext or the top of the HighestNow with the higher score to be the next member of the Activity Register. It then updates the Sofar pointer and continues looping until the Activity Register is full. Note that the Anumber is increased by Compute from 6 to 12 at BigLoop = 999 (TimeToStorm-1 where TimeToStorm=1000)

NowExtractionSub First, it takes all the elements that have been selected so far in the new line of the Activity Register and extracts the addresses in their Now fields and scores them in the NowScoreRegister(element address). Outputs are not scored. Signs are taken into account in increasing or decreasing the score. If UseIDP=1 and the element in the Activity Register is the IDP, the routine calls on

IDPNowExtractionSub. If an element is already in the Activity Register, it is given a NowScoreRegister() of 0.

IDPNowExtractionSub extracts how often an address has been referred to in the Now field of the IDP element; this gives a score to be added to the NowScoreRegister(element address). Signs are taken into account in increasing or decreasing the score.

EmergenceSub multiplies the NextScoreRegister() and the NowScoreRegister() by a factor to give the EmergentNextScoreRegister() and the EmergentNowScoreRegister(). This factor is determined via CompatibilitySub. Here, it equals 1 except for the case of inputs with inputs in the NowScoreRegister(input, input) where it equals 0. Note that Compute puts EmergentNextScoreRegister() = NextScoreRegister() (to allow the option of using EmergenceSub)

DoubleEntrySub takes all the elements so far present in the Activity Register and puts all their scores to zero.

NowOrderSub puts EmergentNowScoreRegister() = NowScoreRegister() (because the EmergenceSub is largely unused) and then it crudely ranks the scores in the NowScoreRegister() as HighestNow(top,1) = address and HighestNow(top,2) = its score, then HighestNow(second to top,1) = its address and HighestNow(second to top,2) = its score, etc.

ScrambleHighestNowSub prevents an artefact which could occur with supposedly independent runs of the program in which the same Nows could be selected each run despite there being other Nows with the same score (this is due to NowOrderSub putting the lowest addresses on top (e.g. if element-25 and element-37 have the same score, element-25 would always be selected). This routine therefore scrambles the order of elements with the same score.

NextReorderSub finds the element that has just been selected as the HighestNow in the new as yet unfinished line of the Activity Register and then re-orders the HighestNexts from that entry onwards (thereby preventing an element selected via the NowScores from being re-selected via the NextScores)

Compute continued

ForcedOutputSub generates an output at random that is forced into the Activity Register

ForcedElementInsertionSub is called on once when it is the time for a storm. It finds elements to be inserted that are NOT in the learnt sequence in the Activity Register (i.e., have a StormDownTime>0) N.B., elements that have recently been in the Activity Register have a StormDownTime=1. A completely new line is created to represent a major perturbation

DetectionOutputSub counts how many outputs there are in the new line of the Activity Register. It records a failure if there is more than one output. If there is no output at all and if OutputLacking > ForcedOutputProbability then it signals that OutputNeeded = 1 (which will call up ForcedOutputSub). Unless there is no output and no output is needed, it calls TestDecisionRewardOrPunishSub

TestDecisionRewardOrPunishSub DefineInputOutputSub has defined the desired outputs as OutputA(1), OutputA(2) and OutputA(3) as = 1 if we want Enumber, Enumber-1 and Enumber-2, respectively, as outputs. If the program has advanced to the number of loops > LoopsTillStorm, there is neither rewarding nor punishing. To punish, a random line within an input-output sequence is chosen as start and then PunishMutateNextSub and PunishMutateNowSub are called on to act on this and subsequent lines several times according to RepeatRewardPunish (here 5). To reward, all the lines between the input and the output are chosen (plus the previous output line if this was good). Then ShortRewardNextSub and ShortRewardNowSub are called on.

ShortRewardNowSub takes each address in the line of the Activity Register as Element1 and then selects at random Element2; it then writes the address of Element2 into the Now field of Element1 (unless Element1=Element2 or Element1=IDP element). With the present max size of the fields, a bias limit is not used. It avoids overwriting the connections of a previously successful element because it detects whether this element has a field of maximum size. It lengthens the Now field.

ShortRewardNextSub acts like ShortRewardNowSub but takes Element2 from the next line of the Activity Register to the line containing Element1. It lengthens the Next field

PunishMutateNextSub takes each element with an address in the line of the Activity Register and writes a random address into its Next field which it also shortens. IDP1 and IDP2 are exempt.

PunishMutateNowSub takes each element with an address in the line of the Activity Register and writes a random address into its Now field which it also shortens. IDP1 and IDP2 are exempt.

UpdateLineNumberSub increases the number of the line of the Activity Register by 1.

ShowResults displays the highest scoring Now and Next addresses of the elements, the success and fail graph, and calls on Maurice_AffTbActivity

Maurice_AffTbActivity Shows the 16 most recent lines of the Activity Register.

Code for CocoIDP825

```
%
'This trimmed-down program is based on CocoInsertionIDP825 is based on IDP CocoPerturbationIDP805 ... is based on 'Coco75 and Coco ANN.'                    %
'                                                                                   %
'                    26 December 2022                                    %
'Task: 1-1000, 2-999, 3-999, 2-1000, 3-998 ...
%
'%%%%%%%%%%%%%%%%%%%%%%%%%%%%%%%%%%%%%%%%%%%%%%%%%%%%%%%%%%%%%%%%%%%%%%%%%%%%%%%%%%%%%%%%%%%%%%%%%%%%%%
%%%
'Make sure that the program has learnt before it reaches LoopsTillStorem=1000
'before continuing (else the results will be meaningless)
'If it has not learnt, simply start again.

Option Explicit      ' VB avertira quand un nom de variable créé dans le code ne figure pas dans les déclarations ci-dessous
Option Base 1        ' Affecte la valeur 1 au plus bas niveau d'indice d'un tableau. La seule autre valeur possible est 0

'CHOICES:
'Emergence and compatibility do not operate at present but would do if the values in these subroutines were changed from 1
Const Test = 0 'put to 0 to avoid preloading a winning pattern; REMEMBER to alter testcompatibilitysub in Initialize
Const MetaLoop = 1 'Allows the program to run for a long time if greater than one
Const NowNextWeighting = 1 'Changes the relative importance of the Now and Next connections when filling the Activity Register
'Const CyclePermission = 1 '=0 for no cycling and = 1 for cycling - now a variable depending on success/failure
Const CycleLength = 1000 'typically set to 4 to cause cycling every 4 lines of Activity Register
Const ForcedOutputProbability = 1 'OutputLacking goes up by 1 for each line where there is no output (it is reset by detection of output)
'if the counter, OutputLacking, > ForcedOutputProbability Then OutputNeeded
Const NoiseLevel = 0 'for noise to cause insertion of a random element into the ActivityRegister,
        'NoiseLevel has to be greater than R50 which is random between 1 and 20 (hence NoiseLevel has to be greater than 0
Const MutationThreshold = 90 'mutation threshold is only usedin the PunishMutate routines, range is from 1 to 100,
          'high means few mutations are inserted during punishing MutationNow Int((100 * Rnd) + 1) < MutationThreshold then no punishing
Const ActivatePunish = 1 'If ActivatePunish = 0 then the punish routines are disabled
Const ActivateReward = 1 'If ActivateReward = 0 then the reward routines are disabled
Const KeepInputSameForThisNumber = 1 'Typically 1 for immediate cycling of inputs
Const NowTooBiasedLimit = 10 'typically = NowNextRange / 2
Const NextTooBiasedLimit = 10
Const RemoveInputByCoco = 0 'if there is a real input, a second artefactual one is removed
Const Enumber = 1000              ' Ces 4 paramètres étant cités dans des Dim
Const AnumberMax = 100              ' must be much less than Enumber
Const KNextMin = 9
Const KNowMin = 8
Const KNextMax = 16
Const KNowMax = 1
Const NowNextRange = 16 'divided by two gives the midpoint in Initialize. Also should equal (KNextMax - KNowMax)+1
```

```
Const IDPNowNextRange = 120
Const IDPSparing = 5 'must be less than IDPNowNextRange/2
Const DowntimeNumberRange = 0      ' Plus the constant in InitializeDowntimeSub
Const EndOfActivityRegister = 25000 'REMOVE and restore to 150
Const RepeatRewardPunish = 5
Const InputRange = 4
Const OutputRange = 3
Const InputTotalMax = 6
Const OutputRangeMax = 50
Const OutputRangeMin = 100

Const UseIDP = 0 'choose =1 to use the IDP and = 0 not to use it
Const IDP = 10 'choose this element to be highly connected
Const LoopsTillStorm = 1000

'END OF CHOICES


' %%%%%%%%%%%%%%%%%%%%%%%%%%%%% Maurice - 05-06-2008 - Déclarations pour séquences
Maurice Affichages %%%%%%%%%%%%%%%%%%%%%%%%
Dim Susp As Integer                                                          '%
Dim Stopper As Integer                                                        '%
Dim Ctr As Integer                ' Pour comptage des captures Excel                 '%
Dim Save_faite As Integer            ' Pour fichier Coco_Capture                     '%
'Dim xlApp As Excel.Application          ' Déclaration des Objets Excel  - Application          '%
'Dim xlBook As Excel.Workbook            '        - id -       - Classeur                '%
'Dim xlSheet As Excel.Worksheet          '        - id -       - Feuille                 '%
Dim Spy, Esp, Esp1, xe, ye As Integer        ' Pour diverses séquences Maurice               '%
'
%%%%%%%%%%%%%%%%%%%%%%%%%%%%%%%%%%%%%%%%%%%%%%%%%%%%%%%%%%%%%%%%%%%%%%%%%%%%%%%%%%%%%%%%%%%%%%%%%%%%%%%%%%%%%%%%%%%%%%%%%%%%%%%%%%%%%%%%%%%%%%%%%%%%%%%%%%%%%%%%%%%%%%%%%%%%%%%%%%%%%%%%%%%%
%%

Dim InactivateThisNext As Integer
Dim TempNext1 As Integer
Dim TempNext2 As Integer
Dim TempNext3 As Integer
Dim TempNext4 As Integer
Dim TempNext5 As Integer
Dim TempNext6 As Integer
Dim TempNext7 As Integer
Dim TempNext8 As Integer
Dim TempNext9 As Integer
Dim TempNext10 As Integer
Dim TempNow1 As Integer
Dim TempNow2 As Integer
Dim TempNow3 As Integer
Dim TempNow4 As Integer
Dim TempNow5 As Integer
Dim TempNow6 As Integer
Dim TempNow7 As Integer
Dim TempNow8 As Integer
Dim TempNow9 As Integer
Dim TempNow10 As Integer
```

```
Dim ThisOne As Integer
Dim Counter As Integer
Dim Rank As Integer
Dim OrderedNext(Enumber, 2) As Integer
Dim NewAnumber As Integer
Dim Downtime(Enumber) As Integer
Dim DowntimeMax(Enumber) As Integer
Dim InsertionScore As Integer
Dim FirstForcedElement As Integer
Dim TimeForStorm As Integer
Dim AnumberCounter As Integer
Dim StormElement As Integer
Dim StormDownTime(Enumber) As Integer
Dim Position As Integer
Dim BinNow(Enumber) As Integer
Dim BinNext(Enumber) As Integer
Dim BinnedScore As Integer

Dim ZeroNow As Integer
Dim ZeroNext As Integer
Dim ConnectivityNow(Enumber) As Integer
Dim ConnectivityNext(Enumber) As Integer

Dim IDPElement As Integer
Dim KNextNumber(Enumber) As Integer
Dim KNowNumber(Enumber) As Integer
Dim NowNextNumberMidpoint As Integer 'defined as equal to half the NowNextRange in Initialize
Dim IDPNowNextNumberMidpoint As Integer
Dim NowGrowing As Integer
Dim NextGrowing As Integer

Dim MutationFieldSize(Enumber) As Integer

Dim ScrambleLowestNumber As Integer
Dim MarkScrambleLowestStart As Integer
Dim TempScrambleLowestAddress As Integer
Dim TempScrambleLowestScore As Integer

Dim UnusedElement(Enumber, 2) As Integer
Dim LowestUsedElement(Enumber, 2) As Integer
Dim TempUnusedElement(2) As Integer

Dim UsedElement(Enumber, 2) As Integer
Dim HighestUsedElement(Enumber, 2) As Integer
Dim TempUsedElement(2) As Integer

Dim SpareLink(Enumber, Enumber) As Integer
Dim LinkFrom As Integer
Dim LinkTo As Integer
Dim MatrixNow(Enumber, Enumber) As Integer
Dim MatrixNext(Enumber, Enumber) As Integer
Dim TempMatrixNow(Enumber, Enumber) As Integer
Dim TempMatrixNext(Enumber, Enumber) As Integer
Dim ValueOfMatrixNow As Integer
Dim ValueOfMatrixNext As Integer
```

```
Dim OutputFrequency(Enumber) As Integer
Dim LineChoice As Integer


Dim CitedElement As Integer
Dim CitingElement As Integer

Dim InputA(InputRange) As Integer
Dim OutputA(OutputRange) As Integer


Dim OutputNowScore(Enumber) As Integer

Dim OutputNeeded As Integer
Dim DontOverwriteNow As Integer
Dim DontOverwriteNext As Integer
Dim DontOverwriteNextOverlap As Integer
Dim CompatibilityTable(Enumber, Enumber) As Integer
Dim FirstElement As Integer
Dim SecondElement As Integer

Dim CounterForPreviousSuccess As Integer
Dim RewardDecision As Integer
Dim PositionAR1 As Integer
Dim PositionNow1 As Integer
Dim OtherPositionsAR1 As Integer
Dim PositionAR2 As Integer
Dim PositionNext1 As Integer
Dim OtherPositionsAR2 As Integer

Dim PositionAR3 As Integer
Dim PositionNext3 As Integer
Dim OtherPositionsAR3 As Integer

Dim PositionAR4 As Integer
Dim PositionNext4 As Integer
Dim OtherPositionsAR4 As Integer

Dim PositionAR5 As Integer
Dim PositionNext5 As Integer

Dim OutputLacking As Integer
Dim ForcedOutputPosition As Integer
Dim MutationFrequency As Integer
Dim MutationPosition As Integer
Dim MutationElement As Integer
Dim MutatedElement As Integer
Dim MutationNow As Integer
Dim MutationNext As Integer
Dim MutationOverlap As Integer

Dim ConstantInput As Integer
Dim InputTotal As Integer
'for Activity register need to indicate linenumber
```

```
Dim LineNumber As Integer
Dim NewLineNumber As Integer
Dim Activity(EndOfActivityRegister, AnumberMax) As Integer
Dim Element(Enumber, IDPNowNextRange) As Integer
Dim SignElement(Enumber, Enumber) As Integer
Dim Sign As Integer

'Calculate most frequent elements
Dim NextScoreRegister(Enumber) As Integer
Dim NowScoreRegister(Enumber) As Integer

Dim EmergentNextScoreRegister(Enumber) As Integer
Dim EmergentNowScoreRegister(Enumber) As Integer


Dim HighestNext(Enumber, 2) As Integer
Dim TempHighestNext(2) As Integer
Dim TempHighestNow(2) As Integer
Dim HighestNow(Enumber, 2) As Integer
'Dim TopNext As Integer
'Dim TopNow As Integer
Dim SavedHighestNext(Enumber, 2) As Integer
Dim SavedHighestNow(Enumber, 2) As Integer


Dim Bigloop0 As Long 'Byte
Dim Bigloop1 As Byte 'unused
Dim A As Integer
Dim E As Integer
Dim K As Integer 'for field within phase element
Dim L As Integer 'for field within phase element
Dim P As Integer 'for phase element
Dim Q As Integer 'for phase element
Dim S As Integer
Dim T As Integer
Dim RandomizeAgain As Integer
Dim InputTally As Integer
Dim JustTestingNow As Integer
Dim JustTestingNext1 As Integer
Dim JustTestingNext2 As Integer
Dim Filler As Integer



'Variables for using the Cycle routine
Dim CycleElement As Integer
Dim CycleStep As Integer
Dim Cycling As Integer
Dim CyclePermission As Integer
'Dim CycleLimit As Integer

'Variables for Scrambling Nows
Dim ScrambleNowNumber As Integer
Dim TempScrambleNowAddress As Integer
Dim TempScrambleNowScore As Integer
```

```
Dim MarkScrambleNowStart As Integer

'Variables for Scrambling Nexts
Dim ScrambleNextNumber As Integer
Dim TempScrambleNextAddress As Integer
Dim TempScrambleNextScore As Integer
Dim MarkScrambleNextStart As Integer

'Variables for Running Score (short term memory of successes)
Dim Adaptation As Integer
Dim UseAdaptation As Integer
Dim DeltaRunningScore As Integer
Dim TimeToUseRunningScore As Integer
Dim RunningScorePointer As Integer
Dim RunningScoreTotal1 As Integer
Dim RunningScoreTotal2 As Integer
Dim RunningScoreTotal As Integer
Dim RunningScore(20000) As Integer 'note that this was Enumber but crashes if Enumber is small
Dim RunningScoreAction As Integer
Dim IncrementRunningScore As Integer
Dim RunningScoreWindow As Integer
Dim RunningScoreLength As Integer
Dim SuccessTableWindow As Integer
Dim PresentResult As Integer
Dim SameResult As Integer

'Variables for neighbourhood connections
Dim MakeLocalConnection As Integer
Dim MakeDistalConnection As Integer
Dim TwoInputs As Integer
Dim MakeLocalNextConnection As Integer
Dim MakeDistalNextConnection As Integer

Dim MakeLocalNowConnection As Integer
Dim MakeDistalNowConnection As Integer
Dim MakeLocalOverlapConnection As Integer
Dim MakeDistalOverlapConnection As Integer

'Victor addition to Coco20atelier 7-6-2008
Dim ModuloRemainder As Integer
Dim WithinLocalNextRangeOutput As Integer
Dim WithinLocalNextRangeInput As Integer
Dim LinkNextOutput  As Integer
Dim LinkNextInput  As Integer
Dim ForceOutput As Integer
Dim DontForceOutput As Integer

'Variables for long term memory
Dim LTMemory(Enumber, Enumber) As Integer

'Victor addition to Coco 20 atelier 7-6-2008
'Variables for phase separation
Dim StuckInLoop As Integer
Dim StuckInDistalLoop As Integer
Dim StuckInLocalLoop As Integer
```

```
Dim NewModulusConnection As Integer
Dim ModulusRandomElement As Integer
Dim ModuloReceiver As Integer
Dim ModuloDonor As Integer
Dim ModuloGroup As Integer
'END Victor addition to Coco 20 atelier 7-6-2008

'Victor addition to Coco 20 atelier 28-6-2008
'This is to test for double entry in the Activity Register
Dim DoubleEntry As Integer
'END Victor addition to Coco 20 atelier 28-6-2008

Dim ZeroNSRTotal As Integer
Dim UnusedTopNow As Integer
Dim UnusedTopNext As Integer
Dim InitialTopNow As Integer
Dim InitialTopNext As Integer

'For rewarding and punishing
Dim RewardInputtoOutput As Integer
Dim PunishInputtoOutput As Integer
Dim RewardOutputtoOutput As Integer
Dim PunishOutputtoOutput As Integer

Dim SumNow As Integer
Dim SumNext As Integer
Dim MeanNow As Integer
Dim MeanNext As Integer
Dim InputNowScore(Enumber) As Integer
Dim InputNextScore(Enumber) As Integer

Dim OldOutputLine As Integer
Dim GoodNewOutput As Integer
Dim GoodOldOutput As Integer

'for phase separation of outputs
Dim TenNowSet(Enumber) As Integer
Dim TwentyNowSet(Enumber) As Integer
Dim TenNextSet(Enumber) As Integer
Dim TwentyNextSet(Enumber) As Integer
Dim PreviousLine As Integer
Dim TenMinusTwentyNow As Integer
Dim TenMinusTwentyNext As Integer

'For recording successes
Dim RecordPointer As Integer
Dim SuccessTable(2000, 3)
Dim OutputResult As Integer
Dim PunishNextLine As Integer
Dim ShortTermMemoryLength As Integer
Dim FullSuccessStory As Integer

'Dim YinYang As Integer
'Dim FailureRecordForYinYang As Integer
'Dim NumberOfYinYangs As Integer
```

```
'Dim YinYangCounter As Integer

Dim ExistingNowLink As Integer
Dim ExistingNextLink As Integer

'Each loop counter is used in ONLY one loop

Dim I0 As Integer
Dim I1 As Integer

Dim I2 As Integer
Dim J2 As Integer

Dim I3 As Integer

Dim I4 As Integer
Dim J4 As Integer
Dim I5 As Integer
Dim I6 As Integer
Dim I7 As Integer
Dim J7 As Integer
Dim I8 As Integer
Dim J8 As Integer
Dim I9 As Integer
Dim J9 As Integer
Dim I10 As Integer
Dim J10 As Integer
Dim I11 As Integer
Dim J11 As Integer
Dim I12 As Integer
Dim I13 As Integer
Dim I14 As Integer
Dim I15 As Integer
Dim I16 As Integer
Dim I17 As Integer
Dim I18 As Integer
Dim J18 As Integer
Dim I19 As Integer
Dim J19 As Integer
Dim I20 As Integer
Dim J20 As Integer
Dim I21 As Integer
Dim I22 As Integer
Dim I23 As Integer
Dim J23 As Integer
Dim I24 As Integer
Dim J24 As Integer
Dim I25 As Integer
Dim J25 As Integer
Dim I26 As Integer
Dim J26 As Integer

Dim I27 As Integer
Dim I28 As Integer
Dim I29 As Integer
```

```
Dim I30 As Integer
Dim I31 As Integer
Dim I32 As Integer
Dim I33 As Integer
Dim I34 As Integer
Dim J34 As Integer
Dim I35 As Integer
Dim J35 As Integer
Dim I36 As Integer
Dim J36 As Integer
Dim I37 As Integer
Dim I38 As Integer
Dim I39 As Integer
Dim I40 As Integer
Dim J40 As Integer
Dim I41 As Integer
Dim I42 As Integer
Dim J42 As Integer
Dim I43 As Integer
Dim J43 As Integer
Dim I44 As Integer
Dim J44 As Integer
Dim I45 As Integer
Dim J45 As Integer
Dim I46 As Integer
Dim J46 As Integer
Dim K46 As Integer
Dim I47 As Integer
Dim J47 As Integer
Dim I48 As Integer
Dim J48 As Integer
Dim I49 As Integer
Dim J49 As Integer
Dim I50 As Integer
Dim J50 As Integer
Dim I51 As Integer
Dim J51 As Integer
Dim I52 As Integer
Dim J52 As Integer
Dim I53 As Integer
Dim I54 As Integer
Dim J54 As Integer
Dim K54 As Integer
Dim I55 As Integer
Dim I56 As Integer
Dim I57 As Integer
Dim I58 As Integer
Dim I59 As Integer
Dim J59 As Integer
Dim I60 As Integer
Dim J60 As Integer
Dim I61 As Integer
Dim I62 As Integer
Dim I63 As Integer
Dim J63 As Integer
```

```
Dim K63 As Integer
Dim L63 As Integer

Dim I64 As Integer
Dim J64 As Integer
Dim I65 As Integer
Dim J65 As Integer
Dim K65 As Integer
Dim I66 As Integer
Dim J66 As Integer
Dim K66 As Integer
Dim I67 As Integer
Dim J67 As Integer
Dim I68 As Integer
Dim J68 As Integer
Dim I69 As Integer
Dim I70 As Integer
Dim J70 As Integer
Dim I71 As Integer
Dim K71 As Integer
Dim I72 As Integer
Dim K72 As Integer
Dim I73 As Integer
Dim I74 As Integer
Dim I75 As Integer
Dim I76 As Integer
Dim J76 As Integer
Dim I77 As Integer
Dim J77 As Integer
Dim I78 As Integer
Dim J78 As Integer
Dim I79 As Integer
Dim J79 As Integer
Dim I80 As Integer
Dim J80 As Integer
Dim I81 As Integer
Dim I82 As Integer
Dim I83 As Integer
Dim I84 As Integer
Dim J84 As Integer
Dim I85 As Integer
Dim I86 As Integer
Dim J86 As Integer
Dim K86 As Integer
Dim I87 As Integer
Dim J87 As Integer
Dim I88 As Integer
Dim J88 As Integer
Dim I89 As Integer
Dim J89 As Integer
Dim I90 As Integer
Dim J90 As Integer
Dim I91 As Integer
Dim J91 As Integer
Dim I92 As Integer
```

```
Dim J92 As Integer
Dim K92 As Integer
Dim I93 As Integer
Dim I94 As Integer
Dim J94 As Integer
Dim K94 As Integer
Dim I95 As Integer
Dim J95 As Integer
Dim I96 As Integer
Dim J96 As Integer
Dim I97 As Integer
Dim I98 As Integer
Dim J98 As Integer
Dim I99 As Integer
Dim J99 As Integer
Dim K99 As Integer
Dim I100 As Integer
Dim J100 As Integer
Dim K100 As Integer
Dim L100 As Integer
Dim I101 As Integer
Dim I102 As Integer
Dim I103 As Integer
Dim I104 As Integer
Dim J104 As Integer

'R is for random
Dim R As Integer
Dim R0 As Integer
Dim R1 As Integer
Dim R2 As Integer
Dim R3 As Integer
Dim R4 As Integer
Dim R5 As Integer
Dim R6 As Integer
Dim R7 As Integer
Dim R8 As Integer
Dim R9 As Integer
Dim R10 As Integer
Dim R11 As Integer
Dim R12 As Integer
Dim R13 As Integer
Dim R14 As Integer
Dim R15 As Integer
Dim R16 As Integer
Dim R17 As Integer
Dim R18 As Integer
Dim R19 As Integer
Dim R20 As Integer
Dim R21 As Integer
Dim R22 As Integer
Dim R23 As Integer
Dim R24 As Integer
Dim R25 As Integer
Dim R26 As Integer
```

```
Dim R27 As Integer
Dim R28 As Integer
Dim R29 As Integer
Dim R30 As Integer
Dim R31 As Integer
Dim R32 As Integer
Dim R33 As Integer
Dim R34 As Integer
Dim R35 As Integer
Dim R36 As Integer
'Victor addition to Coco 20 atelier 12-6-2008
Dim R37 As Integer
Dim R38 As Integer
Dim R39 As Integer
Dim R40 As Integer
Dim R41 As Integer
Dim R42 As Integer
Dim R43 As Integer
Dim R44 As Integer
Dim R45 As Integer
Dim R46 As Integer
Dim R47 As Integer
Dim R48 As Integer
Dim R49 As Integer
Dim R50 As Integer
Dim R51 As Integer
Dim R52 As Integer
Dim R53 As Integer
Dim R54 As Integer
Dim R55 As Integer
Dim R56 As Integer
Dim R57 As Integer
Dim R58 As Integer
Dim RandomElement As Integer
Dim R60 As Integer
Dim R61 As Integer
Dim R62 As Integer
Dim R63 As Integer
Dim R64 As Integer
Dim R65 As Integer
Dim R66 As Integer
Dim R67 As Integer
Dim R68 As Integer
Dim R69 As Integer
Dim R70 As Integer
Dim R71 As Integer
Dim R72 As Integer
Dim R73 As Integer
Dim R74 As Integer

'End Victor addition to Coco 20 atelier 12-6-2008

Dim OnlyScoreOneNow As Integer
Dim OnlyScoreOneNext As Integer
Dim AfterInputPosition As Integer
```

```
Dim AvailablePosition As Integer
Dim Sofar As Integer
Dim Sofartemp As Integer
Dim RewardNow As Integer
Dim RewardNext As Integer

Dim NumberOfOutputs As Integer
Dim RecordSuccess As Long
Dim RecordFailure As Long
Dim InputNeeded As Integer
Dim OutputLinePlusOne As Integer
Dim InputLine As Integer
Dim Reward As Integer
Dim GrowthResponse As Integer
Dim SporulationResponse As Integer
Dim SubtractionOfLines As Integer
Dim StartLoop As Integer
Dim EndLoop As Integer
Dim ActRegLine As Integer
Dim TooBiased As Integer
Dim ForcedOutput As Integer
'

Private Sub Anumber_Change()
If Anumber.Text = "" Then
    Anumber.Text = 1
    Exit Sub
End If
If (Val(Anumber) < 2) Then Anumber.Text = 2
If (Val(Anumber) > AnumberMax) Then Anumber.Text = AnumberMax
End Sub

' %%%%%%%%%%%%%%%%%%%%%%%%%%%%%%%%%%%%%%%%%%%%%% Maurice - 24-05-2008 commande
Pause  %%%%%%%%%%%%%%%%%%%%%%%%%%%%%%%%%%%%%%%%%%%%%%%%%%%%%%
Private Sub Cmd_Susp_Click()                                                     '%
   Susp = 1 - Susp                                                          '%
End Sub                                                                   '%
'
%%%%%%%%%%%%%%%%%%%%%%%%%%%%%%%%%%%%%%%%%%%%%%%%%%%%%%%%%%%%%%%%%%%%%%%
%%%%%%%%%%%%%%%%%%%%%%%%%%%%%%%%%%%%%%%%%%%%%%%%%%%%%%%%%%%%%%%%%%%%%%%
%



' %%%%%%%%%%%%%%%%%%%%%%%%%%%%%%%%%%%%%%%%%%%%%% Maurice - 29-05-2008 commande
Stop  %%%%%%%%%%%%%%%%%%%%%%%%%%%%%%%%%%%%%%%%%%%%%%%%%%%%%%%
Private Sub Stop_Coco_Click()                                                    '%
   Stopper = 1 - Stopper                                                      '%
End Sub                                                                   '%
'
%%%%%%%%%%%%%%%%%%%%%%%%%%%%%%%%%%%%%%%%%%%%%%%%%%%%%%%%%%%%%%%%%%%%%%%
%%%%%%%%%%%%%%%%%%%%%%%%%%%%%%%%%%%%%%%%%%%%%%%%%%%%%%%%%%%%%%%%%%%%%%%
%

Private Sub Bigloop0Max_Change()
```

```
If Bigloop0Max.Text = "" Then
    Bigloop0Max.Text = 1
    Exit Sub
End If
If (Val(Bigloop0Max) < 1) Then Bigloop0Max.Text = 1
If (Val(Bigloop0Max) > 32755) Then Bigloop0Max.Text = 32755
End Sub
'


Private Sub Command1_Click()          ' Command1 = Go     %%%% Maurice 03-06-2008 - Vrai début du
déroulement du code
'
5                                                      ' Repère 5                              '%
   Esp = "Sub Command1_Click"        ' Maurice 24-05-2008 Espionnage                                   '%
   Espion                      ' Maurice 24-05-2008 Espionnage                                   '%
' %%%%%%%%%%%%%%%%%%%%%%%%  Maurice - 24-05-2008 - Chargement feuille Annexe pour Espion
%%%%%%%%%%%%%%%%%%%%%%%%%%%%%%%%%%%
   Load Annexe                   ' Maurice 24-05-2008 - chargement feuille Annexe . . .                     '%
   Annexe.Visible = False           '                  . . . invisible dans le cas général                    '%
'
%%%%%%%%%%%%%%%%%%%%%%%%%%%%%%%%%%%%%%%%%%%%%%%%%%%%%%%%%%%%%%
%%%%%%%%%%%%%%%%%%%%%%%%%%%%%%%%%%%%%%%%%%%%%%%%%%%%%%%%%%%%%%
%%
'
'Show          ' %%%% Maurice 03-06-2008 - inutile, car 'Visible=On' est positionné dans les propriétés de la
feuille        '%
'
Initialize 'If UseInitializeStructure = 1 Then InitializeStructureSub Else Initialize
' %%%%%%%%%%%%%%%%%%%%%%%% Maurice - 29-05-2008 - Initialisation à partir de l'écran
%%%%%%%%%%%%%%%%%%%%%%%%%%%%%%%%%%%%%%
'    Enumber = Enumber_txt.Text        ' Impossible tant que ces variables sont définies                        '%
'    Knumber = Knumber_txt.Text        ' en contantes, ce qui est obligatoire car elles                        '%
'    Anumber = Anumber_txt.Text        ' apparaissent ddans des Dim(variable).                              '%
'    DowntimeNumber = DownTime_txt.Text ' Reste à étudier un autre système !                              '%
'
%%%%%%%%%%%%%%%%%%%%%%%%%%%%%%%%%%%%%%%%%%%%%%%%%%%%%%%%%%%%%%
%%%%%%%%%%%%%%%%%%%%%%%%%%%%%%%%%%%%%%%%%%%%%%%%%%%%%%%%%%%%%%
%%
For I0 = 1 To MetaLoop
Compute
'ShowResults
'Reflect - this will include condensation and expansion
Next I0
   Maurice_AffTbÉlémFinProg          ' %%%% Maurice - 04-06-2008 - vers affichage en fin de run du tableau des
éléments
'
End Sub

Public Sub ShowResults()
   Esp = "Sub ShowResults"           ' Maurice 24-05-2008 - Espionnage
   Espion                      ' Maurice 24-05-2008 - Espionnage
10                                                     ' Repère 10
   I12 = 0
   For I12 = 1 To InputRange
```

```
'       AInput(I12 - 1) = InputA(I12)
    Next I12
    Label4.Caption = ConstantInput
'
    Maurice_AffTbActivity              ' %%%% Maurice - 04-06-2008 - vers affichage en fin de run du tableau
Activity
'
    I16 = 0
    For I16 = 1 To 11
        LHighestNext1(I16 - 1) = SavedHighestNext(I16, 1)
        LHighestNext2(I16 - 1) = SavedHighestNext(I16, 2)

        LHighestNow1(I16 - 1) = SavedHighestNow(I16 + 1, 1)
        LHighestNow2(I16 - 1) = SavedHighestNow(I16 + 1, 2)
    Next I16
'
'    I17 = 0                         '%%%% Maurice 04-06-2008 - Inutile
    'LTopNow = TopNow                  '%%%% Maurice 04-06-2008 - Remise en service du champ sur Feuille
principale  %%%%
    'LTopNext = TopNext                '                          - id -
    LRecordSuccess = RecordSuccess
    LRecordFailure = RecordFailure
'
End Sub

Public Sub InitializeStructureSub()
'This is no longer an alternative to Initialize

Esp = "CreateStructureSub"            ' Maurice 24-05-2008 - Espionnage
    Espion                        ' Maurice 24-05-2008 - Espionnage
30                                                   ' Repère 30

ConnectivityExtractSub
ConnectivityBinSub
ConnectivityDisplaySub
Maurice_AffTbÉlémFinProg

40                                                   ' Repère 40
'HERE IS THE NEW STRUCTURE-CREATING BIT
For I99 = (Enumber / 20) To ((Enumber / 20) * 9) Step 100 '((Enumber / 10) + (4 * Anumber)) Step 100
For J99 = I99 + 1 To (I99 + (6 * Anumber)) 'for Enumber=4000, I99 goes from 200 to 1800
'and J99 from 200 to 260, 300 to 360 etc..
For K99 = 1 To KNextMax
R51 = I99 + Int(4 * Anumber * Rnd) 'R51 = 200+(0 to 39)

Select Case K99
Case Is < KNowNumber(J99)
'do nothing
Case Is < KNextNumber(J99)
Element(J99, K99) = R51
Case Else
End Select

Next K99
Next J99
```

```
Next I99

ConnectivityExtractSub
ConnectivityBinSub
ConnectivityDisplaySub
Maurice_AffTbÉlémFinProg
End Sub

Public Sub Initialize()
   Esp = "Initialize"            ' Maurice 24-05-2008 - Espionnage
   Espion                    ' Maurice 24-05-2008 - Espionnage
30                                                  ' Repère 30

   NewAnumber = 12
   NowNextNumberMidpoint = NowNextRange / 2
   IDPNowNextNumberMidpoint = IDPNowNextRange / 2

   CompatibilitySub 'TestCompatibilitySub3 ' 'TestCompatibilitySub2 '
   RecordSuccess = 0
   RecordFailure = 0
   InputNeeded = 1

'Randomly fills fields of elements with addresses of other elements
'note that arrays are array(row, column)ie Element(Enumber,Knumber)
   I18 = 1
   J18 = 1
   For I18 = 1 To Enumber
   R72 = Int(KNowMin * Rnd) + 1 'e.g., int4xRnd +1 = 1,2,3
   KNowNumber(I18) = R72
   R73 = KNowMin + Int((KNextMax - KNowMin) * Rnd) + 1 'e.g., 3+int(6-3)1=3+(0,1,2)+1
   KNextNumber(I18) = R73
      For J18 = R72 To R73 'KNowMin To KNextMin
InitializeAgain:
         Randomize
         R51 = Int((Enumber * Rnd) + 1)
                  Select Case I18 'avoid connecting 1,2 or 3 to outputs
                  Case Is <= InputRange
                  If R51 > (Enumber - OutputRange) Then GoTo InitializeAgain
                  Case Is > (Enumber - OutputRange)
                  If R51 <= InputRange Then GoTo InitializeAgain
                  Case Else

         End Select

         Element(I18, J18) = R51

      Next J18
   Next I18


'Randomly gives signs to connections
For I40 = 1 To Enumber
 For J40 = 1 To Enumber
 SignElement(I40, J40) = 1
         Randomize
```

```
        R15 = Int((100 * Rnd) + 1)
        If R15 > 10 Then SignElement(I40, J40) = 1 Else SignElement(I40, J40) = -1
 Next J40
Next I40

40                                        ' Repère 40

InitializeStructureSub

InitializeDownTimeSub

If UseIDP = 1 Then InitializeIDPSub

'Removes inputs that occur in the
'initialisation step and that might be confusing
  RemoveSpuriousInputSub

  I21 = 0
'Load Activity Register with zeroes
  For LineNumber = 1 To EndOfActivityRegister
    For I21 = 1 To Anumber
      Activity(LineNumber, I21) = 0
    Next
  Next

  LineNumber = 1
  NewLineNumber = 2

'set first line of Activity register to random values (but not inputs!)
50                                        ' Repère 50

'TEST, OVERWRITE THIS in testsetups

ReLoadFirstLine:
  I22 = 0
  For I22 = 1 To Anumber
SetUpCycle:
    Randomize
    Activity(LineNumber, I22) = Int((Enumber * Rnd) + 1)
    If Activity(LineNumber, I22) <= InputRange Then GoTo SetUpCycle
  Next

I10 = 0
  DoubleEntry = 0
   For I10 = 1 To Anumber - 1
    For J10 = I10 + 1 To Anumber
    If Activity(LineNumber, I10) = Activity(LineNumber, J10) Then DoubleEntry = DoubleEntry + 1
    If DoubleEntry = 0 Then GoTo Noproblem
    DoubleEntry = DoubleEntry + 1
Noproblem:
    Next J10
    Next I10

If DoubleEntry > 0 Then GoTo ReLoadFirstLine
```

```
'Gives initial CycleElement
CycleElement = 1

'gives initial inputs

I10 = 0
   For I10 = 1 To InputRange
   InputA(I10) = 0
   Next I10

   InputA(1) = 1
   InputTotal = 1
   ConstantInput = 1

   CounterForPreviousSuccess = 1
RunningScoreWindow = 10 'REMOVE AND RESTORE =10
RecordPointer = 1
SuccessTable(RecordPointer, 1) = 1 'inputline
SuccessTable(RecordPointer, 2) = 1 'outputline
SuccessTable(RecordPointer, 3) = 0

ConnectivityExtractSub
ConnectivityBinSub
ConnectivityDisplaySub
Maurice_AffTbÉlémFinProg

End Sub

Public Sub InitializeDownTimeSub()

For I103 = 1 To Enumber
DowntimeMax(I103) = Int(DowntimeNumberRange * Rnd) + 12
Next I103
End Sub

Public Sub InitializeIDPSub()

'Loads the IDP with full fields
   KNowNumber(IDP) = 1 'KNowMax
   KNextNumber(IDP) = IDPNowNextRange 'KNextMax for IDP

   IDPElement = IDP + 100
   'this fills the Now and Next fields of the IDP
      For I18 = 1 To IDPNowNextRange - 1 Step 2 'KNowMax To KNextMax
'RepeatInitializeAgain:
         'Randomize
         IDPElement = IDPElement + 1
         Element(IDP, I18) = IDPElement
         Element(IDPElement, KNowMin) = IDP
         SignElement(IDP, IDPElement) = 1 'Gives positive signs to connections between IDP and its favourites
         SignElement(IDPElement, IDP) = 1
         Element(IDP, I18 + 1) = IDPElement
         Element(IDPElement, KNowMin) = IDP
         SignElement(IDP, IDPElement) = 1 'Gives positive signs to connections between IDP and its favourites
         SignElement(IDPElement, IDP) = 1
```

```
      Next I18

'Increase links to the IDP from the other elements
For I18 = InputRange To (Enumber - OutputRange)
      R15 = Int((100 * Rnd) + 1)
         If R15 > 10 Then GoTo MissIDP
         If I18 = IDP Then GoTo MissIDP

      For J18 = KNowMin To KNextMin

         Randomize
         'R51 = InputRange + Int(((Enumber - (InputRange + OutputRange)) * Rnd) + 1) 'inputRange=4
outputrange=3; so must be >3 and <4998

         Element(I18, J18) = IDP
         SignElement(Element(I18, J18), IDP) = 1
         SignElement(IDP, Element(I18, J18)) = 1
      Next J18
MissIDP:
   Next I18

ConnectivityExtractSub
ConnectivityBinSub
ConnectivityDisplaySub
Maurice_AffTbÉlémFinProg

End Sub


Public Sub CompatibilitySub()
   'RESTORE THIS WHEN TESTING FINISHED

   Esp = "Compatibility"            ' Maurice 24-05-2008 - Espionnage
   Espion                       ' Maurice 24-05-2008 - Espionnage

'could make random compatibility groups between 1 and 10 for all elements except for inputs
'but instead makes full compatibilities
For I63 = 1 To Enumber
For J63 = (I63 + 1) To (Enumber - 1)
Randomize
CompatibilityTable(I63, J63) = 1 'was Int((10 * Rnd) + 1)
CompatibilityTable(J63, I63) = 1 ' was = CompatibilityTable(I63, J63)
Next J63
Next I63

'makes full compatibilities for inputs and all other elements upto first half of Enumber
For I63 = 1 To InputRange
For J63 = (InputRange + 1) To Enumber / 2
CompatibilityTable(I63, J63) = 1  'was 10
CompatibilityTable(J63, I63) = 1 'was 10
Next J63
Next I63

'makes full compatibilies for inputs with second half of elements (but could make this incompatibilities
For I63 = 1 To InputRange
```

```
For J63 = ((Enumber / 2) + 1) To Enumber
CompatibilityTable(I63, J63) = 1
CompatibilityTable(J63, I63) = 1
Next J63
Next I63

'makes full incompatibilities between inputs
For I63 = 1 To InputRange
For J63 = (I63 + 1) To InputRange
CompatibilityTable(I63, J63) = 0 ' was 1 remove
CompatibilityTable(J63, I63) = 0 ' was 1 remove
Next J63
Next I63

'Diagnonal selfing compatibilities
For I63 = 1 To Enumber
CompatibilityTable(I63, I63) = 1 'was 10
Next I63

End Sub

Public Sub TestSetUpWinningSeries2()

End Sub


Public Sub WhatShouldWeDoSub()

End Sub


Public Sub Compute() '4/02/2011

   Esp = "Compute"             ' Maurice 24-05-2008 - Espionnage
   Espion                      ' Maurice 24-05-2008 - Espionnage
'this Bigloop comprises most of the program; it starts by loading inputs

   Picture1.Line (0, 0)-(0, 0)

   EnumberLabel.Text = Enumber
   KnumberLabel.Text = NowNextRange
   Anumber.Text = Anumber
   DownTimeLabel = DowntimeNumberRange

   For Bigloop0 = 1 To Bigloop0Max '0

   LBoucle = Bigloop0

    'Clear NewLine:
      I31 = 0
      For I31 = 1 To Anumber
      Activity(NewLineNumber, I31) = 0
      Next I31

   InactivateElementsSub 'If DowntimeNumberRange > 0 Then InactivateElementsSub
```

```
   'CycleLimit = 10 * Int(Enumber / 10)

   If CyclePermission = 1 Then CycleSub

         'The following is needed for a mutational storm
         Select Case Bigloop0 'This gives the label StormDownTime(element) to the elements in the Active subset
         Case Is < LoopsTillStorm - 30
         'do nothing
         Case Is < LoopsTillStorm - 1
         For I101 = 1 To Enumber
         If Downtime(I101) > 0 Then StormDownTime(I101) = 1
         Next I101
         Case Else
         End Select

         Select Case InputNeeded
         Case Is = 0
         Case Is = 1
         Randomize
         R66 = R66 + 1 'Int((4 * Rnd) + 1) doesn't do anything in its present form
         If R66 = 1 Then InputSub
         If R66 = 1 Then R66 = 0
         End Select

   'If InputNeeded = 1 Then InputSub 'along with InputAverageConnectivitySub puts InputNextScore(1 0r 2 0r 3)
to a high value

   NextExtractionSub 'puts Next scores from elements in Activity(Linenumber) into the NextScoreRegister
   'EmergenceSub is not used here even if there is an input or a cyclic element and the first HighestNext has yet
to be loaded
   'and Activity(NewLine, 1) = 0.

   'The following is because EmergentNextScoreRegister has not been filled in EmergenceSub
     I4 = 0
      For I4 = 1 To Enumber
      EmergentNextScoreRegister(I4) = NextScoreRegister(I4) 'EmergentNextScoreRegister is used by the
following NextOrderSub
      Next I4

   NewNextOrderSub 'NextOrderSub     'NewNextOrderSub
   ScrambleHighestNextSub
   FindFirstAvailablePositionSub 'FINDS FIRST AVAILABLE POSITION IN ACTIVITY REGISTER, loads HighestNext as
Sofar

SavedHighestNext(1, 1) = HighestNext(1, 1)
SavedHighestNext(1, 2) = HighestNext(1, 2)
70                                                  ' Repère 70

Select Case Bigloop0
Case Is < LoopsTillStorm - 100
SofarSub
Case Is = LoopsTillStorm - 1
Anumber = NewAnumber
SofarSub
```

```
Case Else
Anumber = NewAnumber
SofarSub
End Select

'SofarSub 'LOAD ACTIVITY REGISTER USING NOW/NEXT COMPETITION BY OBTAINING HIGHEST NOW EACH
CYCLE
'CHECK THAT THERE ARE NO ZEROES IN ACTIVITY REGISTER ELSE WILL GET AN ERROR FROM element (A1, A2)
SINCE A2 WILL EQUAL ZERO'

120                                     ' Repère 120

If Bigloop0 < (LoopsTillStorm - 200) And OutputNeeded = 1 Then ForcedOutputSub 'remove?

      Select Case UseIDP
      Case Is = 0
          Select Case TimeForStorm
          Case Is = 0
          If Bigloop0 >= LoopsTillStorm And InputTotal = 5 Then ForcedElementsInsertionSub
'MutationStormSub '
          If Bigloop0 >= LoopsTillStorm And InputTotal = 5 Then TimeForStorm = 1

          Case Else
          'do nothing
          End Select
      Case Is = 1
           Select Case TimeForStorm
           Case Is = 0
           If Bigloop0 >= LoopsTillStorm And InputTotal = 5 Then ForcedElementsInsertionSub
'IDPMutationStormSub 'ForcedElementsInsertionSub '
           If Bigloop0 >= LoopsTillStorm And InputTotal = 5 Then TimeForStorm = 1
           Case Else
          'do nothing
           End Select
      End Select

'eliminates a second input from Coco
If RemoveInputByCoco = 1 Then RemoveInputGenerationByCocoSub

'sets variables to zero
  Reward = 0
  RewardNow = 0
  RewardNext = 0

If InputNeeded = 0 Then DetectionOutputSub 'detects the presence, nature and number of outputs

'ShowResults 'disable this and enable it in SofarSub if you want to follow loading the ActivityRegister step by
step

UpdateLineNumberSub
140                                     ' Repère 140
'This is to avoid dividing by zeroes
  If RecordSuccess = 0 Then RecordSuccess = 1
  If RecordFailure = 0 Then RecordFailure = 1
```

```
   Picture1.Line -((Bigloop0 / Bigloop0Max) * 100, RecordSuccess / (RecordSuccess + RecordFailure) * 100),
QBColor(0)
145
   ShowResults
   DoEvents
Next Bigloop0    'end of bigloop0        ' Maurice 05-06-2008 - Pour s'assurer que le Next est bien attribué à
Bigloop0 - Repère Paquet

ConnectivityExtractSub
ConnectivityBinSub
ConnectivityDisplaySub
Maurice_AffTbÉlémFinProg

End Sub

Public Sub RemoveZeroAndDoubleEntrySub()
I10 = 0
   For I10 = 1 To Anumber
      Randomize
      If Activity(NewLineNumber, I10) = 0 Then Activity(NewLineNumber, I10) = Int((Enumber * Rnd) + 1)
   Next I10

RerunDoubleEntry:

   I10 = 0
   DoubleEntry = 0
    For I10 = 1 To Anumber - 1
      For J10 = I10 + 1 To Anumber
      If Activity(NewLineNumber, I10) = Activity(NewLineNumber, J10) Then DoubleEntry = DoubleEntry + 1
      If DoubleEntry = 0 Then GoTo Noproblem
      Activity(NewLineNumber, I10) = Int((Enumber * Rnd) + 1)

      DoubleEntry = DoubleEntry + 10

Noproblem:
      Next J10
      Next I10

      If DoubleEntry > 0 Then GoTo RerunDoubleEntry
      'If DoubleEntry > 0 Then WhatShouldWeDoSub
End Sub
Public Sub FindFirstAvailablePositionSub()

'after this routine, Sofar points to a filled position
Sofar = 0
   I5 = 0
   For I5 = 1 To Anumber
      AvailablePosition = 0
      If Activity(NewLineNumber, I5) = 0 Then AvailablePosition = 1
      'By inactivating the following line, I take control away from the previous AR Line
      If AvailablePosition = 1 Then Activity(NewLineNumber, I5) = HighestNext(1, 1) 'There should ALWAYS be a
HighestNext to insert
      If AvailablePosition = 1 Then Rank = 2 'this is the pointer for the Nexts
      If AvailablePosition = 1 Then Sofar = I5
      If AvailablePosition = 1 Then I5 = Anumber
```

```
   Next I5

End Sub

Public Sub SoFarAnumberIncreaseSub()
'

End Sub
Public Sub SofarSub()

'Sofar points to a filled space in the Activity Register

   For Sofartemp = Sofar To (Anumber - 1)

Select Case Activity(NewLineNumber, Sofar)

Case Is <= InputRange 'The element is an input

 I62 = 0
   For I62 = 1 To InputRange
   InputNowScore(I62) = 0
   InputNextScore(I62) = 0
   Downtime(I62) = DowntimeMax(I62)
   Next I62

Case Else

End Select


NowExtractionSub
'NextExtractionSub
'If OutputNeeded = 1 Then ForcedOutputSub 'remove and restore this, maybe ...

R50 = Int((20 * Rnd) + 1)
If NoiseLevel < R50 Then GoTo MakeNoNoise

'If (RunningScoreTotal / RunningScoreWindow) > (1 / 4) Then GoTo MakeNoNoise

         Randomize
         R50 = Int(RunningScoreWindow * Rnd) - 1 'make this - 1 if want to avoid all Noise after successful
learning

         If (RunningScoreTotal / RunningScoreWindow) > (R50 / RunningScoreWindow) Then GoTo MakeNoNoise

NoiseSub

MakeNoNoise:

EmergenceSub

DoubleEntrySub

NowOrderSub
```

```
ScrambleHighestNowSub 'This is to avoid the artefact in which Nows with the same score always have the lowest address on top

'NextOrderSub

'ScrambleHighestNextSub

SavedHighestNext(Sofartemp + 1, 1) = HighestNext(Rank, 1)
SavedHighestNext(Sofartemp + 1, 2) = HighestNext(Rank, 2)
SavedHighestNow(Sofartemp + 1, 1) = HighestNow(1, 1)
SavedHighestNow(Sofartemp + 1, 2) = HighestNow(1, 2)

'ShowResults 'enable this and disable it in SofarSub if you want to follow loading the ActivityRegister step by step


110                                              ' Repère 110
   Select Case HighestNext(Rank, 2)
   Case Is <= (NowNextWeighting * HighestNow(1, 2))
   Activity(NewLineNumber, Sofar + 1) = HighestNow(1, 1) 'Inactivation will be done by NowExtractionSub
   InactivateThisNext = HighestNow(1, 1)
   NextReorderSub
   Case Else
   Activity(NewLineNumber, Sofar + 1) = HighestNext(Rank, 1)
   Rank = Rank + 1
   End Select

'in case of a bizarre problem:
If Activity(NewLineNumber, Sofar + 1) = 0 Then Activity(NewLineNumber, Sofar + 1) = Int(Enumber * Rnd) + 1

   Sofar = Sofar + 1

'End of Sofartemp loop
Next Sofartemp

End Sub

Public Sub DoubleEntrySub()
I9 = 0
J9 = 0

   For I9 = 1 To Enumber
      For J9 = 1 To Sofar
         If I9 = Activity(NewLineNumber, J9) Then NowScoreRegister(I9) = 0
         If I9 = Activity(NewLineNumber, J9) Then NextScoreRegister(I9) = 0
         If I9 = Activity(NewLineNumber, J9) Then EmergentNowScoreRegister(I9) = 0
         If I9 = Activity(NewLineNumber, J9) Then EmergentNextScoreRegister(I9) = 0
      Next J9

   Next I9
End Sub


Public Sub TemporaryResetNextScoreRegisterSub()
For I67 = 1 To Sofar
```

```
J67 = Activity(NewLineNumber, I67)
NextScoreRegister(J67) = 0

Next I67

NextOrderSub
End Sub

Public Sub ExemptSuccessfulOutputLinksSub() 'IMPORTANT KNumber has not been modified

For I94 = 1 To Enumber
For J94 = 1 To Enumber
SpareLink(I94, J94) = SpareLink(I94, J94) - 1
If SpareLink(I94, J94) < 0 Then SpareLink(I94, J94) = 0
Next J94
Next I94


For I94 = 1 To OutputRange
For J94 = 1 To Anumber
If OutputA(I94) = 1 And Activity(NewLineNumber, J94) = (1 + Enumber) - I94 Then LinkTo =
Activity(NewLineNumber, J94)
Next J94
Next I94

For J94 = 1 To Anumber
LinkFrom = Activity(NewLineNumber, J94)
For K94 = 1 To NowNextNumberMidpoint
If Element(LinkFrom, K94) = LinkTo Then SpareLink(LinkFrom, LinkTo) = 2
Next K94
Next J94

For J94 = 1 To Anumber
LinkFrom = Activity(NewLineNumber - 1, J94)
For K94 = 1 To NowNextNumberMidpoint
If Element(LinkFrom, NowNextNumberMidpoint + K94) = LinkTo Then SpareLink(LinkFrom, LinkTo) = 2
Next K94
Next J94

End Sub

Public Sub DetectionOutputSub()
  NumberOfOutputs = 0

I11 = 0
  For I11 = 1 To Anumber
  For J11 = 1 To OutputRange
    If Activity(NewLineNumber, I11) = (1 + Enumber) - J11 Then NumberOfOutputs = NumberOfOutputs + 1
    Next J11
  Next I11

I11 = 0

  OutputLinePlusOne = NewLineNumber + 1
```

```
   If OutputLinePlusOne = EndOfActivityRegister + 1 Then OutputLinePlusOne = 1
'InputLine has been set by the InputSubroutine
130                                                    ' Repère 130

   Select Case NumberOfOutputs

   Case 0
   'There is no output (NumberOfOutputs = 0) and there are 2 possibilities:

            Select Case OutputLinePlusOne

            Case InputLine
               '1/ The Activity Register is full and OutputLinePlusOne = InputLine
               InputNeeded = 1
               RecordFailure = RecordFailure + 1

            Case Else
               '2/ The Activity Register is not full and OutputLinePlusOne <> InputLine

            InputNeeded = 0
            Randomize
            OutputLacking = OutputLacking + 1

            If OutputLacking > ForcedOutputProbability Then OutputNeeded = 1 '

            End Select

   Case 1
   'There is a single output - but is it the desired one?
      TestDecisionRewardOrPunishSub 'remove
      InputNeeded = 1
      OutputLacking = 0

   Case Else
   'There is more than one output so there must be punishment
      TestDecisionRewardOrPunishSub 'remove
      InputNeeded = 1
      OutputLacking = 0 'remove

      DoEvents
   End Select
End Sub



Public Sub ForcedOutputSub()

DontForceOutput = 0 'new routine to remove

For I78 = 1 To Anumber
For J78 = 1 To OutputRange
If Activity(NewLineNumber, I78) = (1 + Enumber - J78) Then DontForceOutput = 1
Next J78
Next I78
```

```
If DontForceOutput = 1 Then GoTo MissForcingOutput

Randomize
R54 = Int(OutputRange * Rnd)
Activity(NewLineNumber, Anumber) = Enumber - R54 'CHECK THIS!!!!!!!!!!

MissForcingOutput:

OutputNeeded = 0

End Sub

Public Sub IDPNextExtractionSub()
   For J2 = (IDPNowNextNumberMidpoint + 1) To IDPNowNextRange
      Randomize
      If Element(A, J2) = 0 Then Element(A, J2) = Int((Enumber * Rnd) + 1)

If Downtime(Element(A, J2)) > 0 Then GoTo NoScoringOfOutputNexts3

'Stop cyclic element being loaded twice
If Element(A, J2) = CycleElement Then GoTo NoScoringOfOutputNexts3
S = Element(A, J2)

Sign = SignElement(A, Element(A, J2))
If Sign >= 0 Then NextScoreRegister(S) = NextScoreRegister(S) + 1
If Sign < 0 Then NextScoreRegister(S) = NextScoreRegister(S) - 1

NoScoringOfOutputNexts3:

   Next J2
   NextScoreRegister(IDP) = IDPSparing
End Sub
Public Sub NextExtractionSub()
   Esp = "NextExtractionSub"
   Espion

'Extracts how often an address has been referred to in the Next field of the elements active in the Activity Register.
'linenumber selects 'the line in the Activity Register and I2 selects the element within it; J+Knumber selects the elements within its Next field

'I1 Sets NextScoreRegister and HighestNext to zero
I1 = 0
   For I1 = 1 To Enumber
      NextScoreRegister(I1) = 0
      HighestNext(I1, 1) = 0
      HighestNext(I1, 2) = 0
   Next I1

I2 = 0
J2 = 0
S = 0
For I2 = 1 To Anumber
   A = Activity(LineNumber, I2)
```

```
 'No scoring from outputs
   If A > Enumber - OutputRange Then GoTo NoScoringOfOutputNexts1

   If A = IDP And UseIDP = 1 Then IDPNextExtractionSub
   If A = IDP And UseIDP = 1 Then GoTo DoneIDPNextExtraction
   If A = 0 Then GoTo ThisIsAnumberIncreaseProblem
      For J2 = (NowNextNumberMidpoint + 1) To KNextNumber(A)
      Randomize
      If Element(A, J2) = 0 Then Element(A, J2) = Int((Enumber * Rnd) + 1)
      If Downtime(Element(A, J2)) > 0 Then GoTo NoScoringOfOutputNexts2

      'Stop cyclic element being loaded twice
      If Element(A, J2) = CycleElement Then GoTo NoScoringOfOutputNexts2
      S = Element(A, J2)

      Sign = SignElement(A, Element(A, J2))
      If Sign >= 0 Then NextScoreRegister(S) = NextScoreRegister(S) + 1
      If Sign < 0 Then NextScoreRegister(S) = NextScoreRegister(S) - 1

NoScoringOfOutputNexts2:

      Next J2

NoScoringOfOutputNexts1:
DoneIDPNextExtraction:

Next I2
ThisIsAnumberIncreaseProblem:

I61 = 0
For I61 = 1 To InputRange
If InputA(I61) = 1 Then NextScoreRegister(I61) = NextScoreRegister(I61) + InputNextScore(I61)
'If InputA(I61) = 1 Then Downtime(I61) = 0 '1 'DowntimeMax(I61)
Next I61

For I77 = 1 To Enumber
If Downtime(I77) > 0 Then NextScoreRegister(I77) = 0
Next I77

If UseIDP = 1 Then NextScoreRegister(IDP) = 5

End Sub


Public Sub IDPNowExtractionSub()
For J7 = 1 To IDPNowNextNumberMidpoint
      If Element(A, J7) = 0 Then Element(A, J7) = Int((Enumber * Rnd) + 1)
      If Downtime(Element(A, J7)) > 0 Then GoTo NoScoringOfIDPNows
      S = Element(A, J7)
      Sign = SignElement(A, S)
If Sign > 0 Then NowScoreRegister(S) = NowScoreRegister(S) + 1
If Sign < 0 Then NowScoreRegister(S) = NowScoreRegister(S) - 1

NoScoringOfIDPNows:
  Next J7
```

```
End Sub
Public Sub NowExtractionSub()
'FIND HIGHEST NOW VALUES FROM ELEMENTS IN ACTIVITY AND LOAD INTO NowScoreRegister

'Sets NowScoreRegister and Highest Now to zero
90                                                  ' Repère 90
    I6 = 0
    For I6 = 1 To Enumber
      NowScoreRegister(I6) = 0
      HighestNow(I6, 1) = 0
      HighestNow(I6, 2) = 0
    Next I6


I7 = 0
J7 = 0
For I7 = 1 To Sofar
  A = Activity(NewLineNumber, I7)
   'Do not score Output fields
    If A > Enumber - OutputRange Then GoTo NoScoringOfOutputNows

    Randomize
    If A = 0 Then A = Int((Enumber * Rnd) + 1)
    If A = IDP And UseIDP = 1 Then IDPNowExtractionSub
    If A = IDP And UseIDP = 1 Then GoTo DoneIDPNowExtraction

    For J7 = (KNowNumber(A)) To NowNextNumberMidpoint
    If Element(A, J7) = 0 Then Element(A, J7) = Int((Enumber * Rnd) + 1)

    If Downtime(Element(A, J7)) > 0 Then GoTo NoScoringOfNows

    S = Element(A, J7)
    Sign = SignElement(A, S)
If Sign > 0 Then NowScoreRegister(S) = NowScoreRegister(S) + 1
If Sign < 0 Then NowScoreRegister(S) = NowScoreRegister(S) - 1


NoScoringOfNows:

   Next J7
NoScoringOfOutputNows:
NoScoringOfInputNows:
DoneIDPNowExtraction:

Next I7

'Ensure that a Now that is ALREADY in the ActivityRegister does not get scored (and perhaps loaded again)
  I76 = 0
  For I76 = 1 To Sofar
   A = Activity(NewLineNumber, I76)
   If A = 0 Then WhatShouldWeDoSub
  If A = 0 Then GoTo DontResetNowScore
  NowScoreRegister(A) = 0
  NextScoreRegister(A) = 0
DontResetNowScore:
```

```
   Next I76

   For I76 = 1 To Enumber
   If Downtime(I76) > 0 Then NowScoreRegister(I76) = 0
   Next I76

End Sub

Public Sub NewNextOrderSub()

For I4 = 1 To Enumber
OrderedNext(I4, 2) = 0
Next I4

   For I4 = 1 To Enumber
   HighestNext(I4, 1) = I4
   HighestNext(I4, 2) = EmergentNextScoreRegister(I4) 'These are the scores modified by EmergenceSub
   'If HighestNext(I4, 2) > 0 Then WhatShouldWeDoSub
   Next I4


For Rank = 1 To Anumber
For I4 = 1 To Enumber
If OrderedNext(Rank, 2) > HighestNext(I4, 2) Then GoTo MissOrder
OrderedNext(Rank, 2) = HighestNext(I4, 2)
OrderedNext(Rank, 1) = HighestNext(I4, 1)
ThisOne = HighestNext(I4, 1)
MissOrder:
Next I4

HighestNext(ThisOne, 2) = 0
Next Rank

For Rank = 1 To Anumber
HighestNext(Rank, 1) = OrderedNext(Rank, 1)
HighestNext(Rank, 2) = OrderedNext(Rank, 2)
Next Rank

End Sub

Public Sub NextReorderSub()
'called on by SofarSub
'first find the element that has appeared in the new as yet unfinished Activity line
'then re-order the HighestNexts from that entry onwards

For I104 = 1 To (Anumber - 1) 'should it be 2 x anumber?
If HighestNext(I104, 1) <> InactivateThisNext Then GoTo NotFoundIt
   For J104 = I104 To Anumber
   HighestNext(J104, 1) = HighestNext(J104 + 1, 1)
   HighestNext(J104, 2) = HighestNext(J104 + 1, 2)
   Next J104
I104 = Anumber
NotFoundIt:
Next I104
End Sub
```

```
Public Sub NextOrderSub()

      Esp = "NextOrderSub"                  ' Maurice 24-05-2008 - Espionnage
   Espion
'(3)use NextScoreRegister so most frequent are ordered in HighestNext

80                                                   ' Repère 80

   TempHighestNext(1) = 0
   TempHighestNext(2) = 0

   I4 = 0
   For I4 = 1 To Enumber
   HighestNext(I4, 1) = I4
   HighestNext(I4, 2) = EmergentNextScoreRegister(I4) 'These are the scores modified by EmergenceSub
   Next I4

I4 = 0
J4 = 0
For I4 = 1 To (Enumber - 1)
For J4 = (I4 + 1) To Enumber
If HighestNext(I4, 2) >= HighestNext(J4, 2) Then GoTo KeepHighestNext
TempHighestNext(1) = HighestNext(I4, 1)
TempHighestNext(2) = HighestNext(I4, 2)
HighestNext(I4, 1) = HighestNext(J4, 1)
HighestNext(I4, 2) = HighestNext(J4, 2)
HighestNext(J4, 1) = TempHighestNext(1)
HighestNext(J4, 2) = TempHighestNext(2)

KeepHighestNext:
Next J4
Next I4

End Sub

Public Sub EmergenceSub()

For I64 = 1 To Sofar
For J64 = 1 To Enumber
If Activity(NewLineNumber, I64) = 0 Then GoTo ZeroAddressError 'this is an error condition

Select Case CompatibilityTable(Activity(NewLineNumber, I64), J64)

Case Is < 1
EmergentNowScoreRegister(J64) = NowScoreRegister(J64) * 0 'remove l
EmergentNextScoreRegister(J64) = NextScoreRegister(J64) * 1 'remove 1

Case Is = 1
EmergentNowScoreRegister(J64) = NowScoreRegister(J64) * 1 'was * 1 / 2
EmergentNextScoreRegister(J64) = NextScoreRegister(J64) * 1 'was * 1 / 2
Case Is = 2
EmergentNowScoreRegister(J64) = NowScoreRegister(J64) * 10 'was * 1 / 2
EmergentNextScoreRegister(J64) = NextScoreRegister(J64) * 10 'was * 1  / 2
Case Is = 3
```

```
EmergentNowScoreRegister(J64) = NowScoreRegister(J64) * 1 'was * 1 / 2
EmergentNextScoreRegister(J64) = NextScoreRegister(J64) * 1 'was * 1  / 2
Case Is = 4
EmergentNowScoreRegister(J64) = NowScoreRegister(J64) * 1 'was * 1 / 2
EmergentNextScoreRegister(J64) = NextScoreRegister(J64) * 1 'was * 1 / 2
Case Is = 5
EmergentNowScoreRegister(J64) = NowScoreRegister(J64) * 1 'was * 1  / 2
EmergentNextScoreRegister(J64) = NextScoreRegister(J64) * 1 'was * 1 / 2
Case Is = 6
EmergentNowScoreRegister(J64) = NowScoreRegister(J64) * 1 'was 2
EmergentNextScoreRegister(J64) = NextScoreRegister(J64) * 1 'was 2
Case Is = 7
EmergentNowScoreRegister(J64) = NowScoreRegister(J64) * 1 'was 2
EmergentNextScoreRegister(J64) = NextScoreRegister(J64) * 1 'was 2
Case Is = 8
EmergentNowScoreRegister(J64) = NowScoreRegister(J64) * 1 'was 2
EmergentNextScoreRegister(J64) = NextScoreRegister(J64) * 1 'was 2
Case Is = 9
EmergentNowScoreRegister(J64) = NowScoreRegister(J64) * 1 'was 2
EmergentNextScoreRegister(J64) = NextScoreRegister(J64) * 1 'was 2

Case Is > 9
EmergentNowScoreRegister(J64) = NowScoreRegister(J64) * 1 'was 3
EmergentNextScoreRegister(J64) = NextScoreRegister(J64) * 1 'was 3

End Select
Next J64
Next I64

ZeroAddressError:

End Sub

Public Sub NowOrderSub() '7/02/2011
  Esp = "NowOrderSub"                    ' Maurice 24-05-2008 - Espionnage
   Espion

TempHighestNow(1) = 0
TempHighestNow(2) = 0

      'I8 = 0

   For I8 = 1 To Enumber
   HighestNow(I8, 1) = I8
   HighestNow(I8, 2) = EmergentNowScoreRegister(I8)
    Next I8


      I8 = 0
      J8 = 0
For I8 = 1 To (Enumber - 1)
For J8 = (I8 + 1) To Enumber
If HighestNow(I8, 2) >= HighestNow(J8, 2) Then GoTo KeepHighestNow
TempHighestNow(1) = HighestNow(I8, 1)
TempHighestNow(2) = HighestNow(I8, 2)
```

```
HighestNow(I8, 1) = HighestNow(J8, 1)
HighestNow(I8, 2) = HighestNow(J8, 2)
HighestNow(J8, 1) = TempHighestNow(1)
HighestNow(J8, 2) = TempHighestNow(2)

KeepHighestNow:
Next J8

Next I8


End Sub

Public Sub NoiseSub()

'this forcibly inserts a randomly chosen element (that is not an input) into the Activity Register
Esp = "NoiseSub"
   Espion

'R49 = 100 + Int((100) * Rnd) 'to get elements between 100 and Enumber
R49 = Int((Enumber - (InputRange + 1)) * Rnd) + (InputRange + 1) 'to get elements between 1 and Enumber
If R49 > Enumber - OutputRange Then GoTo NoNoise


NowScoreRegister(R49) = 10 * NowNextRange * Anumber


NoNoise:
End Sub


Public Sub CycleSub()
   Esp = "CycleSub"                  ' Maurice 24-05-2008 - Espionnage
   Espion                            ' Maurice 24-05-2008 - Espionnage

Cycling = 0
'CycleLength says how many lines of the ActivityRegister there are per insertion of a CycleElement

CycleStep = CycleStep + 1
If CycleStep < CycleLength Then GoTo MissCycle

If CycleStep = CycleLength Then CycleStep = 0


CycleElement = CycleElement + 1
If CycleElement > Enumber Then CycleElement = 4
If CycleElement < 4 Then CycleElement = 4
If CycleElement > Enumber - OutputRange Then CycleElement = 4

Activity(NewLineNumber, 1) = CycleElement

'Let other routines know a CycleElement has been inserted
Cycling = 1

MissCycle:
```

```
End Sub

Public Sub ScrambleHighestNowSub()
   Esp = "ScrambleHighestNowSub"            ' Maurice 24-05-2008 - Espionnage
   Espion                              ' Maurice 24-05-2008 - Espionnage
'The problem to be solved (if it is a problem) is to prevent the same Nows from being loaded again and again even though
'there are other Nows with the same score. This is because the ordering routine puts the lowest addresses on top. So scramble them.
ScrambleNowNumber = 0
MarkScrambleNowStart = 0

For I49 = 1 To Anumber - 1
If MarkScrambleNowStart = 0 Then MarkScrambleNowStart = I49
If HighestNow(I49, 2) = HighestNow(I49 + 1, 2) Then ScrambleNowNumber = ScrambleNowNumber + 1

Select Case ScrambleNowNumber

Case Is = 0
'There is just one line so don't try to scramble!
MarkScrambleNowStart = 0
GoTo ContinueUpdatingNow

Case Is > 0
'If HighestNow(I49, 2) = HighestNow(I49 + 1, 2) Then GoTo ContinueUpdatingNow
'Scramble HighestNows with the same score by swapping
   Randomize
   R20 = Int((2 * Rnd)) '
      Select Case R20
      Case Is = 0
      Case Else
      TempScrambleNowAddress = HighestNow(MarkScrambleNowStart, 1)
      TempScrambleNowScore = HighestNow(MarkScrambleNowStart, 2)
      HighestNow(MarkScrambleNowStart, 1) = HighestNow(MarkScrambleNowStart + 1, 1)
      HighestNow(MarkScrambleNowStart, 2) = HighestNow(MarkScrambleNowStart + 1, 2)
      HighestNow(MarkScrambleNowStart + 1, 1) = TempScrambleNowAddress
      HighestNow(MarkScrambleNowStart + 1, 2) = TempScrambleNowScore
      End Select
ScrambleNowNumber = 0
MarkScrambleNowStart = 0

End Select

ContinueUpdatingNow:

Next I49

End Sub

Public Sub ScrambleHighestNextSub()
'THIS ROUTINE IS PROBABLY NONSENSICAL!!!!!
   Esp = "ScrambleHighestNextSub"          ' Maurice 24-05-2008 - Espionnage
   Espion                             ' Maurice 24-05-2008 - Espionnage
'The problem to be solved (if it is a problem) is to prevent the same Nexts from being loaded again and again even though
```

```
'there are other Nexts with the same score. This is because the ordering routine puts the lowest addresses on top. So scramble them.
ScrambleNextNumber = 0
MarkScrambleNextStart = 0
TempScrambleNextAddress = Enumber

'this goes through the ranked HighestNext array
For I52 = 1 To Anumber - 1
If MarkScrambleNextStart = 0 Then MarkScrambleNextStart = I52
If HighestNext(I52, 2) = HighestNext(I52 + 1, 2) Then ScrambleNextNumber = ScrambleNextNumber + 1

Select Case ScrambleNextNumber
Case Is = 0
'There is just one line so don't try to scramble!
MarkScrambleNextStart = 0
GoTo ContinueUpdatingNext
Case Is > 0
'If HighestNext(I52, 2) = HighestNext(I52 + 1, 2) Then GoTo ContinueUpdatingNext
'Scramble HighestNows with the same score by swapping
   Randomize
   R21 = Int((2 * Rnd)) 'this gives 0 and 1
   Select Case R21
   Case Is = 0
   Case Else
   TempScrambleNextAddress = HighestNext(MarkScrambleNextStart, 1)
   TempScrambleNextScore = HighestNext(MarkScrambleNextStart, 2)
   HighestNext(MarkScrambleNextStart, 1) = HighestNext(MarkScrambleNextStart + 1, 1)
   HighestNext(MarkScrambleNextStart, 2) = HighestNext(MarkScrambleNextStart + 1, 2)
   HighestNext(MarkScrambleNextStart + 1, 1) = TempScrambleNextAddress
   HighestNext(MarkScrambleNextStart + 1, 2) = TempScrambleNextScore
   End Select
ScrambleNextNumber = 0
MarkScrambleNextStart = 0

End Select

ContinueUpdatingNext:

Next I52

End Sub

Public Sub InactivateElementsSub()
   Esp = "InactivateElementsSub"            ' Maurice 24-05-2008 - Espionnage
   Espion                            ' Maurice 24-05-2008 - Espionnage
'This prevents elements that have been active in the AR from being active again
'for a down time

'This allows neurones to recover progressively
   For I38 = 1 To Enumber
      Downtime(I38) = Downtime(I38) - 1
      If Downtime(I38) < 0 Then Downtime(I38) = 0
   Next I38

   For I39 = 1 To Anumber
```

```
        A = Activity(LineNumber, I39)
        If A = 0 Then GoTo AnumberIncreased
        Downtime(A) = DowntimeMax(A)
AnumberIncreased:
    Next I39
    'This stops the IDP being degraded/inactivated:

     If UseIDP = 1 Then Downtime(IDP) = 0

'Must not prevent a response else cannot have 10 in successive rows when needed!

For I85 = 1 To OutputRange
Downtime(1 + Enumber - I85) = 0
Next I85

End Sub

Public Sub TempSuccessfulConnectionsSub()

If LineNumber < 2 Then GoTo MissTempSuccessfulConnections

CitedElement = Activity(NewLineNumber, Sofar + 1) 'This is the latest element to be loaded into the
ActivityRegister

Select Case CitedElement

    Case Is = HighestNow(1, 1) 'if the cited element equals this then strengthen the Now link
    I90 = 0
    J90 = 0

    For I90 = 1 To Sofar
    CitingElement = Activity(NewLineNumber, I90)
        For J90 = (KNowNumber(CitingElement)) To NowNextNumberMidpoint
        If Element(CitingElement, J90) = CitedElement Then TempMatrixNow(CitingElement, CitedElement) = 1
        Next J90
    Next I90

    Case Is = HighestNext(1, 1) 'if the cited element equals this then strengthen the Next link
    I90 = 0
    J90 = 0

    For I90 = 1 To Sofar
    CitingElement = Activity(LineNumber, I90)
        For J90 = (NowNextNumberMidpoint + 1) To (KNextNumber(CitingElement))
        If Element(CitingElement, J90) = CitedElement Then TempMatrixNext(CitingElement, CitedElement) = 1
        Next J90
    Next I90

End Select

MissTempSuccessfulConnections:

End Sub

Public Sub SuccessfulConnectionsSub()
```

```
For I91 = 1 To Enumber
For J91 = 1 To Enumber
MatrixNow(I91, J91) = MatrixNow(I91, J91) + TempMatrixNow(I91, J91)
MatrixNext(I91, J91) = MatrixNext(I91, J91) + TempMatrixNext(I91, J91)
Next J91
Next I91

I91 = 0
J91 = 0

For I91 = 1 To Enumber
For J91 = 1 To Enumber

If MatrixNow(I91, J91) > 10 < 20 Then MatrixNow(I91, J91) = 25
If MatrixNow(I91, J91) = 25 Then GoTo EndMatrixNowUpdate
If MatrixNow(I91, J91) > 19 < 30 Then MatrixNow(I91, J91) = 35
If MatrixNow(I91, J91) = 35 Then GoTo EndMatrixNowUpdate
If MatrixNow(I91, J91) > 29 < 50 Then MatrixNow(I91, J91) = 45
If MatrixNow(I91, J91) = 45 Then GoTo EndMatrixNowUpdate
If MatrixNow(I91, J91) >= 45 Then MatrixNow(I91, J91) = 60

EndMatrixNowUpdate:

If MatrixNext(I91, J91) > 10 < 20 Then MatrixNext(I91, J91) = 25
If MatrixNext(I91, J91) = 25 Then GoTo EndMatrixNextUpdate
If MatrixNext(I91, J91) > 19 < 30 Then MatrixNext(I91, J91) = 35
If MatrixNext(I91, J91) = 35 Then GoTo EndMatrixNextUpdate
If MatrixNext(I91, J91) > 29 < 50 Then MatrixNext(I91, J91) = 45
If MatrixNext(I91, J91) = 45 Then GoTo EndMatrixNextUpdate
If MatrixNext(I91, J91) >= 49 Then MatrixNext(I91, J91) = 60

EndMatrixNextUpdate:
Next J91
Next I91

For I91 = 1 To Enumber
For J91 = 1 To Enumber
TempMatrixNow(I91, J91) = 0
TempMatrixNext(I91, J91) = 0
Next J91
Next I91

End Sub


Public Sub SuccessTableBasedDecisionRewardOrPunishSub() '25/3/2011
Esp = "SuccessTableBasedDecisionRewardOrPunishSub"            ' Maurice 24-05-2008 - Espionnage
  Espion
' input sequence is 1, 2, 3, 2, 1, 2, 3 … for outputs 10 20 20 10 10 20 20
'Recall, InputTotal = 1, or 2 for growth (needs 10 as output)
'and InputTotal = 3 for sporulation (needs 20 as output)
'note that having more than one copy of the right output is also punishable

RewardDecision = 0
```

```
RunningScoreAction = 0
GoodNewOutput = 0


   Select Case InputTotal


   Case 1 '1 needs 10
      If GrowthResponse = 1 And SporulationResponse = 0 Then GoodNewOutput = 1

   Case 2  '2 here needs 20'
      If SporulationResponse = 1 And GrowthResponse = 0 Then GoodNewOutput = 1

   Case 3  '3 needs 20
      If SporulationResponse = 1 And GrowthResponse = 0 Then GoodNewOutput = 1

   'Case 4  '2 here needs 10 RESTORE THIS WHEN NO LONGER TESTING
      'If GrowthResponse = 1 And SporulationResponse = 0 Then GoodNewOutput = 1

   End Select

   Select Case GoodNewOutput

   Case Is = 0
   RecordPointer = RecordPointer + 1
SuccessTable(RecordPointer, 1) = InputLine
SuccessTable(RecordPointer, 2) = NewLineNumber
SuccessTable(RecordPointer, 3) = 0
      RecordFailure = RecordFailure + 1

Case Is = 1
RecordPointer = RecordPointer + 1
SuccessTable(RecordPointer, 1) = InputLine
SuccessTable(RecordPointer, 2) = NewLineNumber
SuccessTable(RecordPointer, 3) = 1
      RecordSuccess = RecordSuccess + 1

End Select

SuccessTableWindow = SuccessTableWindow + 1
If SuccessTableWindow = 1 Then SuccessTableSub 'remove
If SuccessTableWindow = 1 Then SuccessTableWindow = 0


  DoEvents
End Sub

Public Sub TestDecisionRewardOrPunishSub()
RewardDecision = 0
RunningScoreAction = 0
GoodNewOutput = 0

For I84 = 1 To OutputRange
For J84 = 1 To Anumber
If OutputA(I84) = 1 And Activity(NewLineNumber, J84) = (1 + Enumber) - I84 Then GoodNewOutput = 1
```

```
Next J84
Next I84

      If NumberOfOutputs > 1 Then GoodNewOutput = 0

   Select Case GoodNewOutput

   Case Is = 0 'CHOICE OF PUNISHMENTS
       'Example: StartLoop=17 where there is an output of 3998 (happens to be right); the following input is in
line 18
       'Set Endloop=18 so the punishing of the Nexts is in the loop from the Next fields of elements in line 17
       'to the elements in the following line BUT here this following line is (Endloop-1)= line 17 so just doing one
line!

       If Bigloop0 > LoopsTillStorm Then GoTo TooLateToPunish

       LineChoice = NewLineNumber - (OldOutputLine + 1)

       StartLoop = (OldOutputLine) + Int(LineChoice * Rnd)
       EndLoop = StartLoop

       For I81 = 1 To RepeatRewardPunish
       StartLoop = (OldOutputLine)
       EndLoop = StartLoop + 1
       PunishMutateNextSub
       Next I81

       'Startloop now =20 (17+1 + a random 2) Endloop=21
       StartLoop = (OldOutputLine + 1) + Int(LineChoice * Rnd)
       EndLoop = NewLineNumber
       For I81 = 1 To RepeatRewardPunish
       PunishMutateNowSub
       Next I81

TooLateToPunish:
       RecordFailure = RecordFailure + 1

      Case Is = 1

       If Bigloop0 > LoopsTillStorm Then GoTo ToolatetoReward

       StartLoop = InputLine
       EndLoop = NewLineNumber

       For I81 = 1 To RepeatRewardPunish
       ShortRewardNowSub
       Next I81

       If GoodOldOutput = 1 Then StartLoop = OldOutputLine Else StartLoop = InputLine 'remove this addition to
Coco64?

       For I81 = 1 To RepeatRewardPunish
       ShortRewardNextSub
       Next I81
```

```
ToolatetoReward:

       RecordSuccess = RecordSuccess + 1

End Select

 GoodOldOutput = GoodNewOutput
 OldOutputLine = NewLineNumber

'This is for MutationSub, which is not used in this version of Coco
RunningScorePointer = RunningScorePointer + 1
If RunningScorePointer > RunningScoreWindow Then RunningScorePointer = 1
   If GoodNewOutput = 1 Then RunningScore(RunningScorePointer) = 1
   If GoodNewOutput = 0 Then RunningScore(RunningScorePointer) = 0

End Sub



Public Sub SuccessTableSub()
   'Esp = "SuccessTableSub"       ' Maurice 24-05-2008 - Espionnage
   'Espion                        ' Maurice 24-05-2008 - Espionnage

StartLoop = SuccessTable(RecordPointer - SuccessTableWindow, 1)

For I73 = (RecordPointer - SuccessTableWindow) To RecordPointer
PresentResult = SuccessTable(I73, 3)
SameResult = 0
If SuccessTable(I73, 3) = SuccessTable(I73 + 1, 3) Then SameResult = 1
EndLoop = SuccessTable(I73, 2)

Select Case SameResult

Case Is = 0 'the result is different so reward or punish the previous set

   Select Case PresentResult
   Case Is = 0 'so punish present set
   PunishMutateNowSub
   PunishMutateNextSub
   'give startloop of new set
   'If I73 + 1 = RecordPointer Then GoTo EndSuccessLoop
   StartLoop = SuccessTable(I73 + 1, 1)

   Case Is = 1 'so reward present set
   ShortRewardNowSub
   ShortRewardNextSub

   'give startloop of new set
   'If I73 + 1 = RecordPointer Then GoTo EndSuccessLoop
   StartLoop = SuccessTable(I73 + 1, 1)
   End Select

Case Is = 1 'just continue updating unless need to exit
   Select Case RecordPointer
   Case Is = I73
```

```
        Select Case PresentResult
        Case Is = 0
        PunishMutateNowSub
        PunishMutateNextSub
        Case Is = 1
        ShortRewardNowSub
        ShortRewardNextSub

        End Select
    Case Else

    End Select

End Select

EndSuccessLoop:

Next I73

End Sub

Public Sub ShortRewardNowSub()

    If ActivateReward = 0 Then GoTo MissRandomRewardNow

I25 = 0
    J25 = 0

    For ActRegLine = StartLoop To EndLoop

For I25 = 1 To Anumber

DontOverwriteNow = 0

AvoidDirectCouplingInputOutput:
AvoidSelfingRandomNow:
            Randomize
            R7 = Int((Anumber * Rnd) + 1)

            FirstElement = Activity(ActRegLine, I25)
            SecondElement = Activity(ActRegLine, R7)
            If FirstElement = 0 Then GoTo AnotherAnumberIncrease4
            If SecondElement = 0 Then GoTo AnotherAnumberIncrease4
            If FirstElement = SecondElement Then GoTo AvoidSelfingRandomNow
            If FirstElement = IDP And UseIDP = 1 Then GoTo DoneIDPShortRewardNow

            TooBiased = 0
            I35 = 0
            For I35 = KNowNumber(FirstElement) To NowNextNumberMidpoint
            If Element(FirstElement, I35) = SecondElement Then TooBiased = TooBiased + 1
            Next I35
            If TooBiased > NowTooBiasedLimit Then GoTo AvoidCouplingInputToSecondHalfEnumberNow:
```

```
        NowGrowing = 0
        If KNowNumber(FirstElement) > KNowMax Then NowGrowing = 1

        Select Case NowGrowing

        Case Is = 0
         Randomize
         R6 = Int((NowNextNumberMidpoint - KNowNumber(FirstElement) * Rnd) + 1)
         'it needs this to learn properly:
          For J25 = 1 To Anumber 'avoid reward routine overwriting a good combination
          If Element(FirstElement, R6) = Activity(ActRegLine, J25) Then DontOverwriteNow = 1
          'If SignElement(FirstElement, Element(FirstElement, R6)) < 0 Then DontOverwriteNow = 1
          Next J25
        If DontOverwriteNow = 0 Then Element(FirstElement, R6) = SecondElement

        Case Is > 0
        KNowNumber(FirstElement) = KNowNumber(FirstElement) - 1
        Element(FirstElement, KNowNumber(FirstElement)) = SecondElement

        End Select

AvoidCouplingInputToSecondHalfEnumberNow:
AvoidCouplingEvenOddOrOddEven:
'AvoidCouplingIDPtoNow:
DoneIDPShortRewardNow:
    Next I25
AnotherAnumberIncrease4:
  Next ActRegLine

MissRandomRewardNow:

End Sub



Public Sub ShortRewardNextSub()

If ActivateReward = 0 Then GoTo MissRandomRewardNext
'If RunningScoreTotal / RunningScoreWindow > 1 / 2 Then GoTo MissRandomRewardNext
'If RunningScoreTotal >= RunningScoreWindow Then GoTo MissRandomRewardNext
  'If DeltaRunningScore > 0 Then GoTo MissRandomRewardNext

  I26 = 0
  J26 = 0
  I35 = 0

  For ActRegLine = StartLoop To (EndLoop - 1)

 For I26 = 1 To Anumber

        DontOverwriteNext = 0

AvoidDirectCouplingInputNextOutput:

        Randomize
```

```
        R9 = Int((Anumber * Rnd) + 1)

        FirstElement = Activity(ActRegLine, I26)
        SecondElement = Activity(ActRegLine + 1, R9)
        If FirstElement = 0 Then GoTo AnotherAnumberIncrease3
        If SecondElement = 0 Then GoTo AnotherAnumberIncrease3
        If FirstElement = SecondElement Then GoTo AvoidCouplingNextElementToItself

        If FirstElement = IDP And UseIDP = 1 Then GoTo DoneIDPShortRewardNext

       TooBiased = 0
        For I35 = (NowNextNumberMidpoint + 1) To KNextNumber(FirstElement)
          If Element(FirstElement, I35) = SecondElement Then TooBiased = TooBiased + 1
        Next I35
        If TooBiased > NextTooBiasedLimit Then GoTo MakeNoRandomNextReward


        NextGrowing = 0
        If KNextNumber(FirstElement) < KNextMax Then NextGrowing = 1

         Select Case NextGrowing

          Case Is = 0

          Randomize
          R8 = NowNextNumberMidpoint + Int(((KNextNumber(FirstElement) - NowNextNumberMidpoint) * Rnd)
+ 1)

           For J26 = 1 To Anumber 'avoid reward routine overwriting a good combination
           If Element(FirstElement, R8) = Activity(ActRegLine + 1, J26) Then DontOverwriteNext = 1
           'Sign = SignElement(FirstElement, Element(FirstElement, R8 + Knumber))
           'If Sign < 0 Then DontOverwriteNext = 1
           Next J26
           If DontOverwriteNext = 0 Then Element(FirstElement, R8) = SecondElement

          Case Is > 0
          KNextNumber(FirstElement) = KNextNumber(FirstElement) + 1
          Element(FirstElement, KNextNumber(FirstElement)) = SecondElement

          End Select

AvoidCouplingInputToSecondHalfEnumberNext:
AvoidCouplingNextElementToItself:
AvoidCouplingEvenEvenOrOddOdd:
AvoidCouplingNext:
AvoidCouplingIDPtoNewElements:
DoneIDPShortRewardNext:

   Next I26
AnotherAnumberIncrease3:
MakeNoRandomNextReward:

   Next ActRegLine

MissRandomRewardNext:
```

```
End Sub


Public Sub DefineInputOutputSub()

'InputRange = 4 is defined in choices
'OutputRange = 2 is defined in choices
'set InputTotalMax to have the size of the pattern
'define inputA(inputrange) as integer
'OutputA(1)= 1 means we want enumber as output
'OutputA(2)= 1 means we want enumber-1 as output
'OutputA(3)= 1 means we want enumber-2 as output


For I82 = 1 To InputRange
InputA(I82) = 0
Next I82

For I83 = 1 To OutputRange
OutputA(I83) = 0
Next I83

'This gives the input pattern and the corresponding output pattern
Select Case InputTotal '1,2,3,4,5 ...1000,999,998,997,996

Case Is = 1
InputA(1) = 1
Downtime(1) = 0
OutputA(1) = 1

Case Is = 2
InputA(2) = 1
Downtime(2) = 0
OutputA(2) = 1

Case Is = 3
InputA(3) = 1
Downtime(3) = 0
OutputA(2) = 1

Case Is = 4
InputA(2) = 1
Downtime(2) = 0
OutputA(1) = 1

Case Is = 5
InputA(3) = 1
Downtime(3) = 0
OutputA(3) = 1

'Case Is = 6
'InputA(6) = 1
```

```
'OutputA(6) = 1

'Case Is = 7
'InputA(7) = 1
'OutputA(7) = 1

'Case Is = 8
'InputA(8) = 1
'OutputA(8) = 1

'Case Is = 9
'InputA(9) = 1
'OutputA(9) = 1
End Select


End Sub

Public Sub InputSub()
   Esp = "InputSub"                        ' Maurice 24-05-2008 - Espionnage
   Espion                                  ' Maurice 24-05-2008 - Espionnage

'START OF INPUT SECTION
'cross-referencing of inputs has not been eliminated in this program

'constantinput allows input to remain unchanged for x turns
'as far as I can see, it just means skipping the input section
280                                                    ' Repère 280
   ConstantInput = ConstantInput + 1
   If ConstantInput < KeepInputSameForThisNumber Then GoTo keepinput
   If ConstantInput = KeepInputSameForThisNumber Then ConstantInput = 1


'the input pattern is only changed when ConstantInput allows the following

   InputTotal = InputTotal + 1
   If InputTotal = InputTotalMax Then InputTotal = 1

DefineInputOutputSub


keepinput: 'load newline of Activity Register
285                                                    ' Repère 285


'If Cycling = 1 there is already an address in the first 'position of the AR NewLine
   AfterInputPosition = 1
If Cycling = 1 Then AfterInputPosition = 2

InputAverageConnectivitySub

InputNeeded = 0

   InputLine = NewLineNumber
```

```
'END OF INPUT SECTION
End Sub

Public Sub InputPerturberSub()

'Loads the perturbing, unstable IDP with full fields
   KNowNumber(15) = KNowMax
   KNextNumber(15) = KNextMax
   Element(15, 1) = 16

      For J18 = (KNowMax + 1) To KNextMax Step 10
         Element(15, J18) = Element(15, J18 - 1) + 1
         Element(15, J18 + 1) = Element(15, J18 - 1) + 1
         Element(15, J18 + 2) = Element(15, J18 - 1) + 1
         Element(15, J18 + 3) = Element(15, J18 - 1) + 1
         Element(15, J18 + 4) = Element(15, J18 - 1) + 1
         Element(15, J18 + 5) = Element(15, J18 - 1) + 1
         Element(15, J18 + 6) = Element(15, J18 - 1) + 1
         Element(15, J18 + 7) = Element(15, J18 - 1) + 1
         Element(15, J18 + 8) = Element(15, J18 - 1) + 1
         Element(15, J18 + 9) = Element(15, J18 - 1) + 1
      Next J18

NextScoreRegister(15) = 400

End Sub


Public Sub InputAverageConnectivitySub()
Esp = "InputAverageConnectivitySub"
   Espion

'The original intention was to allow anticipation by using the Next scores of the previous line - it could still be
done
'This gives a score to the input that should allow it to be inserted BUT make sure that the register is not set to
zero after this!!!!!!!!!


   I32 = 0
   For I32 = 1 To InputRange
      Select Case Bigloop0
      Case Is < (LoopsTillStorm - 200)
      If InputA(I32) = 1 Then InputNowScore(I32) = 112 + (NowNextRange / Enumber) * Anumber 'or could be =
HighestNow(200, 2)
      If InputA(I32) = 1 Then InputNextScore(I32) = 212 + (NowNextRange / Enumber) * Anumber 'or could be =
HighestNext(20 * Anumber, 2)
      Case Else
      If InputA(I32) = 1 Then InputNowScore(I32) = 0
      If InputA(I32) = 1 Then InputNextScore(I32) = 0
      End Select
   Next I32
End Sub

Public Sub LongTermMemorySub()
   Esp = "LongTermMemorySub"              ' Maurice 24-05-2008 - Espionnage
```

```
   Espion                              ' Maurice 24-05-2008 - Espionnage
'The LTM contains the essence of the Elements table and is a matrix Elements x Elements.
'It is updated depending on RunningScore (i.e. the equivalent of laying down a short term memory) if thngs go well
'It is linked to Downtime so that an important connection is not be disrupted readily
'It can be used to reconstruct the Elements table after a period in which there has been no real progress

'Victor addition 11-06-2008

For I54 = 1 To Enumber
For J54 = 1 To Enumber
LTMemory(I54, J54) = 0
Next J54
Next I54


For I54 = 1 To Enumber
For K54 = 1 To NowNextRange
J54 = Element(I54, K54)
'Addition to Coco22 31-07-2008
If J54 = 0 Then GoTo MissZeroElement
LTMemory(I54, J54) = LTMemory(I54, J54) + SignElement(I54, K54)
MissZeroElement:
'End of Addition to Coco22 31-07-2008
Next K54
Next I54

End Sub

Public Sub UpdateLineNumberSub()
   Esp = "UpdateLineNumberSub"                ' Maurice 24-05-2008 - Espionnage
   Espion                              ' Maurice 24-05-2008 - Espionnage
290                                                        ' Repère 290
   LineNumber = LineNumber + 1
   If LineNumber = EndOfActivityRegister + 1 Then LineNumber = 1
   NewLineNumber = LineNumber + 1
   If LineNumber = EndOfActivityRegister Then NewLineNumber = 1
   If LineNumber = 1 Then NewLineNumber = 2
End Sub

Public Sub IDPMutationStormSub()
'change this depending on whether an IDP is used: If MutatedElement = IDP Then GoTo MutateIDPAgain

Maurice_AffTbÉlémFinProg
ConnectivityExtractSub
ConnectivityBinSub
ConnectivityDisplaySub
Maurice_AffTbÉlémFinProg

For I80 = 1 To Enumber Step 2
MutateIDPAgain:
      Randomize
      MutatedElement = I80 'Int((Enumber * Rnd) + 1)
      If MutatedElement = IDP Then GoTo DoNotMutateTheIDP
      'MutationFieldSize(MutatedElement) = Int((NowNextRange * Rnd) + 1)
```

```
        For J80 = KNowNumber(MutatedElement) To KNextNumber(MutatedElement) Step 4
'MutationFieldSize(MutatedElement) Step 2
        MutationElement = Int((Enumber * Rnd) + 1)
        If MutatedElement = MutationElement Then GoTo MutateIDPAgain
        'If MutationElement = IDP Then GoTo MutateIDPAgain
        MutationPosition = J80
        Randomize
        If Element(MutatedElement, MutationPosition) = IDP Then GoTo KeepIDP
        Element(MutatedElement, MutationPosition) = MutationElement
KeepIDP:
        Next J80
DoNotMutateTheIDP:
        Next I80


ConnectivityExtractSub
ConnectivityBinSub
ConnectivityDisplaySub

Maurice_AffTbÉlémFinProg

End Sub

Public Sub MutationStormSub()
'change this depending on whether an IDP is used: If MutatedElement = IDP Then GoTo MutateIDPAgain

Maurice_AffTbÉlémFinProg
ConnectivityExtractSub
ConnectivityBinSub
ConnectivityDisplaySub
Maurice_AffTbÉlémFinProg

For I80 = 1 To Enumber Step 2
MutateThisElementAgain:
        Randomize
        MutatedElement = I80 'Int((Enumber * Rnd) + 1)
        'MutationFieldSize(MutatedElement) = Int((NowNextRange * Rnd) + 1)
        If MutatedElement = IDP And UseIDP = 1 Then GoTo DontMutateTheIDP

        For J80 = KNowNumber(MutatedElement) To KNextNumber(MutatedElement) Step 4
'MutationFieldSize(MutatedElement) Step 2
        MutationElement = Int((Enumber * Rnd) + 1)
        If MutatedElement = MutationElement Then GoTo MutateThisElementAgain
        MutationPosition = J80
        Randomize
        Element(MutatedElement, MutationPosition) = MutationElement
        Next J80

DontMutateTheIDP:
        Next I80


ConnectivityExtractSub
ConnectivityBinSub
```

```
ConnectivityDisplaySub

Maurice_AffTbÉlémFinProg

End Sub

Public Sub ForcedElementsInsertionSub()

'This finds elements to be inserted AT RANDOM that are NOT in the Active subset (i.e., have a
StormDownTime>0)
'because all elements that have recently been in the Active subset have a StormDownTime=1
Maurice_AffTbÉlémFinProg
InsertionScore = KNowMin
For I100 = 1 To Anumber
   For J100 = InputRange To (Enumber - OutputRange)
   If StormDownTime(J100) = 1 Then GoTo NewStormElementNeeded
   If KNowNumber(J100) > InsertionScore Then GoTo NewStormElementNeeded
    If UseIDP = 1 And J100 = IDP Then GoTo NewStormElementNeeded
   StormElement = J100
   InsertionScore = KNowNumber(StormElement)
NewStormElementNeeded:
    Next J100
 StormDownTime(StormElement) = 1
 Downtime(StormElement) = DowntimeMax(StormElement)
 Activity(LineNumber, I100) = StormElement
 Next I100

End Sub


Public Sub StormInactivationLearnedElementsSub()

For I102 = Enumber - OutputRange To InputRange Step -1 'inputrange=4 so 4 should be the last one
If Downtime(I102) > 0 Then Downtime(I102) = DowntimeMax(I102) 'Stops the learnt elements in the Activity
subset from being loaded
Next I102
End Sub
Public Sub MutationSub()

   Select Case Bigloop0


   Case Is < 200


   Case Else

   'There is a common frequency for mutations irrespective of the number of elements
   'so if it is 1/10, we need to allow for different enumbers by calling up the MutationSub Enumber/100 times

   'This changes the severity of reward and punishment according to recent history
   TimeToUseRunningScore = TimeToUseRunningScore + 1

If TimeToUseRunningScore < RunningScoreWindow Then GoTo TooSoonToMutate
RunningScoreTotal = 0
```

```
   For I53 = 1 To RunningScoreWindow
RunningScoreTotal = RunningScoreTotal + RunningScore(I53) 'information comes from
TestDecisionRewardOrPunishSub
Next I53

      Select Case RunningScoreTotal

   Case Is <= RunningScoreWindow - 3
      For I80 = 1 To Enumber / 100
      Randomize
      MutatedElement = Int((Enumber * Rnd) + 1)
      MutationElement = LowestUsedElement(I80, 1) 'Int((Enumber * Rnd) + 1)
      Randomize
      MutationPosition = KNowNumber(MutatedElement) + Int((1 + KNextNumber(MutatedElement) -
KNowNumber(MutatedElement)) * Rnd)
      Randomize

      Element(MutatedElement, MutationPosition) = MutationElement
      Next I80


      TimeToUseRunningScore = 0

      Case Is = RunningScoreWindow - 2
      For I80 = 1 To Enumber / 200
      Randomize
      MutatedElement = Int((Enumber * Rnd) + 1)
      MutationElement = LowestUsedElement(I80, 1) 'Int((Enumber * Rnd) + 1)
      Randomize
     MutationPosition = KNowNumber(MutatedElement) + Int((1 + KNextNumber(MutatedElement) -
KNowNumber(MutatedElement)) * Rnd)
      Randomize

      Element(MutatedElement, MutationPosition) = MutationElement
      Next I80


      TimeToUseRunningScore = 0

      Case Is = RunningScoreWindow - 1
      For I80 = 1 To Enumber / 400
      Randomize
      MutatedElement = Int((Enumber * Rnd) + 1)
      MutationElement = HighestUsedElement(I80, 1) 'Int((Enumber * Rnd) + 1)
      A = HighestUsedElement(I80, 2)
      Randomize
      MutationPosition = KNowNumber(MutatedElement) + Int((1 + KNextNumber(MutatedElement) -
KNowNumber(MutatedElement)) * Rnd)
      Randomize

      Element(MutatedElement, MutationPosition) = MutationElement

      Randomize
      MutatedElement = Int((Enumber * Rnd) + 1)
```

```
        MutationElement = LowestUsedElement(I80, 1) 'Int((Enumber * Rnd) + 1)
        A = LowestUsedElement(I80, 2)
        Randomize
        MutationPosition = KNowNumber(MutationElement) + Int(((KNextNumber(MutationElement) -
KNowNumber(MutationElement)) * Rnd) + 1)

        Randomize

        Element(MutatedElement, MutationPosition) = MutationElement
        Next I80

        TimeToUseRunningScore = 0

        Case Else
        'do nothing

        End Select
'runningscoretotal = maximum?
TooSoonToMutate:

End Select

End Sub

Public Sub UsedElementSub()

For I96 = 1 To Enumber
UsedElement(I96, 1) = I96
Next I96

For I96 = 1 To InputRange
UsedElement(I96, 2) = 0
Next I96

For I96 = Enumber - (OutputRange + 1) To Enumber
UsedElement(I96, 2) = 0
Next I96

For I96 = 1 To Enumber
UsedElement(I96, 2) = UsedElement(I96, 2) - 1
If UsedElement(I96, 2) < 1 Then UsedElement(I96, 2) = 1
Next I96

For I96 = 1 To Enumber
HighestUsedElement(I96, 1) = UsedElement(I96, 1)
HighestUsedElement(I96, 2) = UsedElement(I96, 2)
Next I96

        I95 = 0
        J95 = 0
For I95 = 1 To Enumber - 1
For J95 = (I95 + 1) To Enumber
If HighestUsedElement(I95, 2) >= HighestUsedElement(J95, 2) Then GoTo KeepHighestUsed
TempUsedElement(1) = HighestUsedElement(I95, 1)
TempUsedElement(2) = HighestUsedElement(I95, 2)
```

```
HighestUsedElement(I95, 1) = HighestUsedElement(J95, 1)
HighestUsedElement(I95, 2) = HighestUsedElement(J95, 2)
HighestUsedElement(J95, 1) = TempUsedElement(1)
HighestUsedElement(J95, 2) = TempUsedElement(2)
KeepHighestUsed:
Next J95
Next I95

'scramble
For I95 = 1 To Enumber / 10
Randomize
R71 = Int(Rnd * Enumber / 10) + 1
TempUsedElement(1) = HighestUsedElement(I95, 1)
TempUsedElement(2) = HighestUsedElement(I95, 2)
HighestUsedElement(I95, 1) = HighestUsedElement(R71, 1)
HighestUsedElement(I95, 2) = HighestUsedElement(R71, 2)
HighestUsedElement(R71, 1) = TempUsedElement(1)
HighestUsedElement(R71, 2) = TempUsedElement(2)
If HighestUsedElement(I95, 1) = 0 Then WhatShouldWeDoSub
Next I95

End Sub
Public Sub UnusedElementSub()

'The UnusedElement matrix has two columns, one corresponding to the address of the element and the other to its score
For I96 = 1 To Enumber
UnusedElement(I96, 1) = I96
Next I96

For I96 = 1 To InputRange
UnusedElement(I96, 2) = Enumber 'This excludes inputs from being considered
Next I96

For I96 = Enumber - (OutputRange + 1) To Enumber
UnusedElement(I96, 2) = Enumber 'This excludes outputs
Next I96

For I96 = 1 To Enumber
UnusedElement(I96, 2) = UnusedElement(I96, 2) - 1
If UnusedElement(I96, 2) < 1 Then UnusedElement(I96, 2) = 1
Next I96

For I96 = 1 To Enumber
LowestUsedElement(I96, 1) = UnusedElement(I96, 1)
LowestUsedElement(I96, 2) = UnusedElement(I96, 2)
Next I96


    I95 = 0
    J95 = 0
For I95 = 1 To Enumber - 1
For J95 = (I95 + 1) To Enumber
If LowestUsedElement(I95, 2) <= LowestUsedElement(J95, 2) Then GoTo KeepLowestUsed
TempUnusedElement(1) = LowestUsedElement(I95, 1)
```

```
TempUnusedElement(2) = LowestUsedElement(I95, 2)
LowestUsedElement(I95, 1) = LowestUsedElement(J95, 1)
LowestUsedElement(I95, 2) = LowestUsedElement(J95, 2)
LowestUsedElement(J95, 1) = TempUnusedElement(1)
LowestUsedElement(J95, 2) = TempUnusedElement(2)

KeepLowestUsed:
Next J95
Next I95

'GoTo MissScramble

'scramble
ScrambleLowestNumber = 0
MarkScrambleLowestStart = 0

For I97 = 1 To Enumber - 1
If MarkScrambleLowestStart = 0 Then MarkScrambleLowestStart = I97
If LowestUsedElement(I97, 2) = LowestUsedElement(I97 + 1, 2) Then ScrambleLowestNumber =
ScrambleLowestNumber + 1

Select Case ScrambleLowestNumber

Case Is = 0
'There is just one line so don't try to scramble!
MarkScrambleLowestStart = 0
GoTo ContinueUpdatingLowest

Case Is > 0
If LowestUsedElement(I97, 2) = LowestUsedElement(I97 + 1, 2) Then GoTo ContinueUpdatingLowest
'Scramble LowestUsedElements with the same score by swapping
   Randomize
   R71 = Int((ScrambleLowestNumber * Rnd)) ' check this gives 0 to some number!!!
TempScrambleLowestAddress = LowestUsedElement(MarkScrambleLowestStart, 1)
TempScrambleLowestScore = LowestUsedElement(MarkScrambleLowestStart, 2)
LowestUsedElement(MarkScrambleLowestStart, 1) = LowestUsedElement(MarkScrambleLowestStart + R71, 1)
LowestUsedElement(MarkScrambleLowestStart, 2) = LowestUsedElement(MarkScrambleLowestStart + R71, 2)
LowestUsedElement(MarkScrambleLowestStart + R71, 1) = TempScrambleLowestAddress
LowestUsedElement(MarkScrambleLowestStart + R71, 2) = TempScrambleLowestScore
ScrambleLowestNumber = 0
MarkScrambleLowestStart = 0

End Select

ContinueUpdatingLowest:

Next I97


MissScramble:

End Sub
```

```
Public Sub PunishMutateNowSub()
   'Esp = "PunishMutateNowSub"                ' Maurice 24-05-2008 - Espionnage
   'Espion                              ' Maurice 24-05-2008 - Espionnage

If ActivatePunish = 0 Then GoTo MissOnPunishNows

For ActRegLine = StartLoop To EndLoop

If ActRegLine = 0 Then GoTo MissActRegLineZero
PositionAR1 = 0
PositionNow1 = 0

For PositionAR1 = 1 To Anumber

         FirstElement = Activity(ActRegLine, PositionAR1)
         If FirstElement = 0 Then GoTo AnotherAnumberIncrease2
         If FirstElement = IDP And UseIDP = 1 Then GoTo DoneIDPPunishMutateNow

     If KNowNumber(FirstElement) < KNowMin Then Element(FirstElement, KNowNumber(FirstElement)) = 0

     If KNowNumber(FirstElement) < KNowMin Then KNowNumber(FirstElement) = KNowNumber(FirstElement)
+ 1 'shorten KNow field

      For PositionNow1 = (KNowNumber(FirstElement)) To NowNextNumberMidpoint
Randomize
MutationNow = Int((100 * Rnd) + 1)
If MutationNow < MutationThreshold Then GoTo NoPunishMutateNow
Sevencycle:
           Randomize
           R2 = Int((Enumber * Rnd) + 1)



'Avoid creating inputs at random
If R2 <= InputRange Then GoTo Sevencycle
'Avoid creating outputs at random
'If R2 = 10 Then GoTo Sevencycle 'Remove
'If R2 = 20 Then GoTo Sevencycle 'Remove
'Avoid selfing
If R2 = FirstElement Then GoTo Sevencycle

  'Not used
  Select Case FirstElement
   Case Is <= InputRange
  'If R2 > Enumber / 2 Then GoTo Sevencycle

   Case Is > Enumber - OutputRange
'If R2 < Enumber / 2 Then GoTo Sevencycle 'Remove

   Case Else

  End Select

Element(FirstElement, PositionNow1) = R2
NoPunishMutateNow:
         Next PositionNow1
```

```
DoneIDPPunishMutateNow:
   Next PositionAR1
AnotherAnumberIncrease2:
MissActRegLineZero:
 Next ActRegLine

MissOnPunishNows:

End Sub

Public Sub PunishMutateNextSub()
   'Esp = "PunishMutateNextSub"              ' Maurice 24-05-2008 - Espionnage
   'Espion                             ' Maurice 24-05-2008 - Espionnage

If ActivatePunish = 0 Then GoTo MissOnPunishNexts

If StartLoop = 0 Then StartLoop = 1
For ActRegLine = StartLoop To (EndLoop - 1)
PositionAR2 = 0
PositionNext1 = 0
For PositionAR2 = 1 To Anumber

      FirstElement = Activity(ActRegLine, PositionAR2)
      If FirstElement = 0 Then GoTo AnotherAnumberIncrease1
      'If FirstElement = IDP Then IDPPunishMutateNextSub
      If FirstElement = IDP And UseIDP = 1 Then GoTo DoneIDPPunishMutateNext

      If KNextNumber(FirstElement) > KNextMin Then Element(FirstElement, KNextNumber(FirstElement)) = 0
      If KNextNumber(FirstElement) > KNextMin Then KNextNumber(FirstElement) =
KNextNumber(FirstElement) - 1

      For PositionNext1 = (NowNextNumberMidpoint + 1) To KNextNumber(FirstElement)
            Select Case UseIDP
            Case Is = 0
            'do nothing
            Case Is = 1
            If Element(IDP, PositionNext1) = IDP Then GoTo NoPunishMutateNext
            End Select

         MutationNext = Int((100 * Rnd) + 1)
         If MutationNext < MutationThreshold Then GoTo NoPunishMutateNext

Eightcycle:
         Randomize
         R4 = Int((Enumber * Rnd) + 1)

            'no selfing
            If R4 = FirstElement Then GoTo Eightcycle
            'no spurious input
            If R4 <= InputRange Then GoTo Eightcycle


   'not used
   Select Case FirstElement
```

```
    Case Is <= InputRange
    'If R4 > Enumber / 2 Then GoTo Eightcycle

    Case Is >= Enumber - OutputRange
    'If R4 < Enumber / 2 Then GoTo Eightcycle 'Remove

    Case Else
    'If Activity(ActRegLine, PositionAR2) Mod 2 = R4 Mod 2 Then GoTo Eightcycle

   End Select

Element(FirstElement, PositionNext1) = R4

NoPunishMutateNext:
      Next PositionNext1
DoneIDPPunishMutateNext:
   Next PositionAR2
AnotherAnumberIncrease1:

 Next ActRegLine

MissOnPunishNexts:

End Sub


Public Sub RemoveInputGenerationByCocoSub()
   Esp = "RemoveInputGenerationByCocoSub"      ' Maurice 24-05-2008 - Espionnage
   Espion                              ' Maurice 24-05-2008 - Espionnage
'Eliminates a second input coming from Coco
'If there is a real input, it is in the first position in the AR
'so check the New line and the following lines for a second input and replace it at random
'this sub MUST be disabled if we want to run in an anticipatory mode in which inputs are predicted

I45 = 0
J45 = 0
InputTally = 0
RandomizeAgain = 0

Select Case NewLineNumber

Case Is = InputLine

   For I45 = 1 To Anumber
If Activity(NewLineNumber, I45) > 3 Then GoTo DontDoAnything1

   InputTally = InputTally + 1
   If InputTally < 2 Then GoTo DontDoAnything1
ReplaceInput1:
   Randomize
   RandomizeAgain = 0
   R16 = Int((Enumber * Rnd) + 1)
   If R16 < 4 Then RandomizeAgain = 1
   If R16 > Enumber - OutputRange Then RandomizeAgain = 1
   If RandomizeAgain = 1 Then GoTo ReplaceInput1
```

```
        For J45 = 1 To Anumber
        If R16 = Activity(NewLineNumber, J45) Then RandomizeAgain = 1
        Next J45
    If RandomizeAgain = 1 Then GoTo ReplaceInput1
    Activity(NewLineNumber, I45) = R16
    InputTally = InputTally - 1
'Victor addition to Coco 19 atelier 4-6-2008
DontDoAnything1:
    Next I45

Case Else

    For I45 = 1 To Anumber
If Activity(NewLineNumber, I45) > 3 Then GoTo DontDoAnything2
If Activity(NewLineNumber, I45) = Activity(InputLine, 1) Then GoTo DontDoAnything2 'allows the same input to
be repeated
    InputTally = InputTally + 1
    If InputTally < 1 Then GoTo DontDoAnything2
ReplaceInput2:
    Randomize
    RandomizeAgain = 0
    R16 = Int((Enumber * Rnd) + 1)
    If R16 < 4 Then RandomizeAgain = 1
    If R16 > Enumber - OutputRange Then RandomizeAgain = 1
    If RandomizeAgain = 1 Then GoTo ReplaceInput2
        For J45 = 1 To Anumber
        If R16 = Activity(NewLineNumber, J45) Then RandomizeAgain = 1
        Next J45
    If RandomizeAgain = 1 Then GoTo ReplaceInput2
    Activity(NewLineNumber, I45) = R16
    InputTally = InputTally - 1
'Victor addition to Coco 19 atelier 4-6-2008
DontDoAnything2:
    Next I45

    End Select


End Sub
Public Sub RemoveSpuriousInputSub()
    Esp = "RemoveSpuriousInputSub"            ' Maurice 24-05-2008 - Espionnage
    Espion                                    ' Maurice 24-05-2008 - Espionnage
'Eliminates inputs in all fields so the system cannot learn!
330                                           ' Repère 330
    I20 = 0
    J20 = 0
    For I20 = 1 To Enumber
        For J20 = KNowMin To KNextMin
            If Element(I20, J20) > 3 Then GoTo ThisIsNotAnInput
ReplaceBiCycle:
            Randomize
            R3 = Int((Enumber * Rnd) + 1)
            If R3 < 4 Then GoTo ReplaceBiCycle
            Element(I20, J20) = R3
ThisIsNotAnInput:
```

```
      Next J20
   Next I20
'
End Sub







'
' %%%%%%%%%%%%%%%%%%%%%%%%%%%%%% MAURICE 05-06-2008 - PAQUET DES SÉQUENCES
MAURICE %%%%%%%%%%%%%%%%%%%%%%%%%%%%%%%%%%%%%%%%%%%
'Sub Patch()        ' REMPLACER TOUT LE PAQUET MAURICE PAR CELUI CI-DESSOUS ET SUPPRIMER CETTE LIGNE
Sub
'
' %%%%%%%%%%%%%%%%%%%%%%%%%%%%%% MAURICE 05-06-2008 - PAQUET DES SÉQUENCES
MAURICE %%%%%%%%%%%%%%%%%%%%%%%%%%%%%%%%%%%%%%%%%%%

' %%%%%%%%%%%%%%%%%%%%%%%  Maurice - 05-06-2008 - Événement bouton de commande Spy
%%%%%%%%%%%%%%%%%%%%%%%%%%%%%%%%%%%%%%%%%%%%
'                                                                                   '%
Private Sub Espionnage_Click()                                                      '%
   Spy = 1 - Spy                                                                    '%
   If Spy = 1 Then                                                                  '%
      Annexe.Visible = True                                                         '%
   Else                                                                             '%
      Annexe.Visible = False                                                        '%
   End If                                                                           '%
End Sub                                                                             '%
'
%%%%%%%%%%%%%%%%%%%%%%%%%%%%%%%%%%%%%%%%%%%%%%%%%%%%%%%%%%%%
%%%%%%%%%%%%%%%%%%%%%%%%%%%%%%%%%%%%%%%%%%%%%%%%%%%%%%%%%%%%
%%

'
'%%%%%%%%%%%%%%%%%%%%%%%%%%  Maurice - 05-06-2008 - Fin des affichages en fin de
programme  %%%%%%%%%%%%%%%%%%%%%%%%%%%%%%%%%%%
'

 Private Sub Espion()
'
' %%%%%%%%%%%%%%%%%%%%%%%  Maurice - 05-06-2008 - Enregistrement Espions
%%%%%%%%%%%%%%%%%%%%%%%%%%%%%%%%%%%%%%%%%%%%%%%%%%%%
Espion:                                                                             '%
   If Esp1 <> 1 Then                                                                '%
      xe = 200: ye = -300          ' Passage 1ère fois                              '%
      Esp1 = 1                                                                      '%
      GoTo 2                                                                        '%
   End If                                                                           '%
2                                   'Repère 2                                       '%
'   If xe > 7000 Then               ' Mettre en service si une donnée accompagne le nom de Sub    '%
'      xe = -3800: ye = ye + 250                                                    '%
'   End If                                                                          '%
```

```
   If ye > 13500 Then                  ' Bas de page, on efface et on reprend en haut de page                          '%
      Annexe.Cls                                                                                  '%
      ye = 400                                                                                    '%
   End If                                                                                         '%
'    xe = xe + 4000                    ' Mettre en service si une donnée accompagne le nom de Sub                      '%
   ye = ye + 300                                                                                  '%
   Annexe.CurrentX = xe: Annexe.CurrentY = ye                                                      '%
   Annexe.Print Esp                                                                               '%
Attente:                                                                                          '%
   DoEvents                                                                                       '%
   If Spy = 1 Then GoTo Attente                                                                   '%
'                                                                                                 '%
 End Sub                                                                                          '%
'%%%%%%%%%%%%%%%%%%%%%%%%%%%%  Maurice - 05-06-2008 - Fin enregistrement Espion
%%%%%%%%%%%%%%%%%%%%%%%%%%%%%%%%%%%%%%%%%%%%%%%%%%%%%%%%%

'


'
' %%%%%%%%%%%%%%%%%%%%%%%%%%%%%%%%%%% MAURICE 05-06-2008 - PAQUET DES SÉQUENCES
MAURICE %%%%%%%%%%%%%%%%%%%%%%%%%%%%%%%%%%%%%%%%%%%%%%%%%%
'Sub Patch()        ' REMPLACER TOUT LE PAQUET MAURICE PAR CELUI CI-DESSOUS ET SUPPRIMER CETTE LIGNE
Sub
'
' %%%%%%%%%%%%%%%%%%%%%%%%%%%%%%%%%%% MAURICE 05-06-2008 - PAQUET DES SÉQUENCES
MAURICE %%%%%%%%%%%%%%%%%%%%%%%%%%%%%%%%%%%%%%%%%%%%%%%%%%
'
'
'
'%%%%%%%%%%%%%%%%%%%%%%%%%%%%  Maurice 05-06-2008 - Début affichage tableau Elément sur
Pause  %%%%%%%%%%%%%%%%%%%%%%%%%%%%%%%%%%%%%%%%%%
Public Sub Maurice_AffTbÉlémPause()                                                                '%
'%
   Esp = "Maurice_AffTbÉlémPause"          ' Maurice 03-06-2008 - Espionnage                                   '%
   Espion                              ' Maurice 03-06-2008 - Espionnage                             '%
600                                  ' Repère 600 sur lequel on revient si l'option Pause n'est pas active            '%
   DoEvents                                                                                       '%
   Dim PremierStop As String               ' Maurice 04-05-2008 - Prise en compte de la commande Stop
'%
   If Stopper = 0 Then                                                                            '%
      If PremierStop = "Oui" Then End                                                              '%
   End If                                                                                         '%
   If Stopper = 1 Then                                                                            '%
      PremierStop = "Oui"                                                                         '%
      GoTo 600                                                                                    '%
   End If                                                                                         '%
'
%%%%%%%%%%%%%%%%%%%%%%%%%%%%%%%%%%%%%%%%%%%%%%%%%%%%%%%%%%%%%%%%%%%%%%%%%
%%%%%%%%%%%%%%%%%%%%%%%%%%%%%%%%%%%%%%%%%%%%%%%%%%%%%%%%%%%%%%%%%%%%%%%%%
%%
601
'  %%%%%%%%%%%%%%%%%%%%%%%%%%%%%%%  Maurice 05-06-2008 - Affichages commande Pause
%%%%%%%%%%%%%%%%%%%%%%%%%%%%%%%%%%%%%%%%%%%%%%%%%%%%%%%%%%%%
   Dim Fin_affiche As String               '08-06-2008                                              '%
   Dim xt, yt, i, j, MaxLgn, MaxCol As Integer              ' 08-06-2008                                  '%
```

```
'%%%%%%%%%%%%%%%%%%%%%%%                                                          '%
'Susp = 1 - Susp '<---¤¤¤¤ POUR SIMULATION COMMANDE PAUSE - LAISSER CETTE INSTRUCTION EN
COMMENTAIRE ¤¤¤¤                    '%
'%%%%%%%%%%%%%%%%%%%%%%%                                                          '%
   If Susp = 0 Then                                                            '%
      Fin_affiche = "Non"                     ' Bouton "Pause" est Off, on saute l'affichage               '%
      GoTo 650                                                                '%
   End If                                                                    '%
   If Fin_affiche = "Oui" Then GoTo 600                   ' Attente du bouton "Pause" en position On                   '%
'            %%%%  Test de l'option choisie %%%%           ' %%%% Maurice 03-06-2008 %%%%
'%
'                                                                            '%
   If Enumber > 32 Then MaxLgn = 32 Else MaxLgn = Enumber      ' Pour éviter dépassement de capacité - 08-06-
2008             '%
   If NowNextRange > 10 Then MaxCol = 10 Else MaxCol = NowNextRange      ' Pour éviter dépassement de
capacité - 08-06-2008             '%
'                                                                            '%
   If Option1.Value = True Then GoTo Affiche_Éléments         '                                     '%
   If Option2.Value = True Then GoTo Affiche_LongTermMemorySub    '                                     '%
'                                                                            '%
Affiche_Éléments:                                                                  '%
 Cls                                                                         '%
 Form1.Font.Size = 8                                                                '%
 CurrentX = 3100: CurrentY = 6410                                                         '%
 Print " Now                                      "                   '%
 CurrentX = 3100: CurrentY = 6410                                                         '%
 ForeColor = RGB(255, 0, 0)                                                            '%
 Print "                                          Next"              '%
 ForeColor = RGB(0, 0, 0)                                                             '%
 xt = 1000: yt = 6660                                                               '%
'                                                                            '%
602                                                                          '%
 For i = 1 To MaxLgn                        ' Pour éviter dépassement de capacité - 08-06-2008            '%
'%
    CurrentX = 900: CurrentY = yt                                                        '%
    ForeColor = RGB(0, 0, 255)                                                          '%
    Print i                                                                   '%
    ForeColor = RGB(0, 0, 0)                                                           '%
    For j = 1 To 2 * MaxCol                   ' Pour éviter dépassement de capacité - 08-06-2008            '%
       If j = 11 Then xt = xt + 800 Else xt = xt + 400                                          '%
       If j > 10 Then ForeColor = RGB(255, 0, 0)                                             '%
       CurrentX = xt: CurrentY = yt                                                       '%
       Print Element(i, j)                                                            '%
    Next j                                                                    '%
   ForeColor = RGB(0, 0, 0)                                                            '%
   xt = 1000: yt = yt + 245                                                            '%
 Next i                                                                      '%
   GoTo Fin_Affichage                       ' %%%% Maurice 03-06-2008 %%%%                         '%
'                           '                                                '%
Affiche_LongTermMemorySub:                          '                                    '%
'                                 ' %%%% Maurice 04-06-2008 pour toute la séquence %%%%         '%
'%
   Dim Tltm, Hltm, Vltm, Xltm, Yltm As Integer                                              '%
   Cls                                                                       '%
   CurrentX = 150: Xltm = 150                                                          '%
```

```
    CurrentY = 6450: Yltm = 6450                                                          '%
    ForeColor = RGB(0, 0, 255)                                                            '%
    For Tltm = 1 To MaxLgn                  ' Pour éviter dépassement de capacité - 08-06-2008       '%
       Xltm = Xltm + 580: CurrentX = Xltm: CurrentY = Yltm                                     '%
       Print Tltm                                                                        '%
    Next Tltm                                                                            '%
'                                                                                       '%
    For Hltm = 1 To MaxLgn                  ' Pour éviter dépassement de capacité - 08-06-2008       '%
       Yltm = Yltm + 240                                                                 '%
       CurrentX = 150: Xltm = 150                                                        '%
       CurrentY = Yltm                                                                   '%
       ForeColor = RGB(0, 0, 255)                                                        '%
       Print Hltm                                                                        '%
       ForeColor = RGB(0, 0, 0)                                                          '%
       For Vltm = 1 To MaxLgn               ' Pour éviter dépassement de capacité - 08-06-2008       '%
          Xltm = Xltm + 580: CurrentX = Xltm                                             '%
          CurrentY = Yltm                                                                '%
          Print LTMemory(Hltm, Vltm)                                                     '%
       Next Vltm                                                                         '%
     Next Hltm                                                                           '%
'                                                                                       '%
Fin_Affichage:                      '                                                    '%
    Fin_affiche = "Oui"               '                                                  '%
'                                                                                       '%
' %%%%%%%%%%%%%%%%%%%%%%%%%%%%%%% Maurice 05-06-2008 - Écrire du tableau sur classeur
Excel si nb de captures > 0 %%%%%%%%%%%%%%%
'                    Ce classeur est nommé Coco_Capture.xls et se trouve directement sur C:          '%
%
620                          ' Repère 620                                                '%
'                                                                                       '%
                                                                                   '%
'                                                                                       '%
Séq_Excel:                                                                              '%
 Dim TbExcel(30, 20)                    ' Table miroir du tableau des Elément pour écriture sur Excel    '%
 Dim LgTitreNow, ClTitreNow, LgTitreNext, ClTitreNext, Valij, incr, Couleur As Integer              '%
 Dim ValTitreNow, ValTitreNext, Path As String                                           '%
 LgTitreNow = 3: ClTitreNow = 7: ValTitreNow = "Now"       ' Titrage des tableaux capturés
'%
 LgTitreNext = 3: ClTitreNext = 19: ValTitreNext = "Next"   '                               '%
 Path = "C:\Coco_Capture.xls"              '                                              '%
 If Not Dir(Path) = "" Then Kill Path           ' Suppression du précédent classeur Coco_Capture.xls s'il existe
'%
'                                                                                       '%
    Ctr = Ctr + 1                      ' Compteur numérique, meilleur pour servir d'indice - Repère Paquet '%
  '%                                                                                    %
    For i = 1 To MaxLgn                  ' Installation dans les cellules du tableau capturé - 08-06-2008   '%
       For j = 1 To 2 * MaxCol               '       - id -                 - 08-06-2008    '%
          If j > 10 Then                 '          - id -                              '%
             Couleur = 3                 '          - id -                              '%
             incr = 1                  '          - id -                               '%
          Else                       '          - id -                                '%
             Couleur = 1                 '          - id -                              '%
             incr = 0                  '          - id -                               '%
          End If                      '          - id -                               '%
          'xlSheet.Cells(i + 5, j + incr).Value = Element(i, j)  '       - id -                  '%
```

```
%
      Next j                                ' 	- id -                              '%
   Next i                                  '	- id -                              '%
'                                                                                '%
   GoTo 600                                                                        '%
Sauvegarde:                                 '                                      '%
   'xlSheet.SaveAs "C:\Coco_Capture.xls"             ' Nommer et sauvegarder le fichier Coco_Capture
'%
   'xlBook.Close                            ' Fermer le fichier                          '%
   Save_faite = 1                                                                  '%
'   xlApp.Quit                              ' Quitter Excel                            '%
'   Set xlApp = Nothing                         ' Déactivation des objets    Inutile (semble-t-il ?)          '%
'   Set xlBook = Nothing                        '      - id -                          '%
'   Set xlSheet = Nothing                       '      - id -                          '%
'                                                                                '%
%
   GoTo 600                                                                        '%
650                                  ' Repère 650                                   '%
'                                                                                '%
' %%%%%%%%%%%%%%%%%%%%%%%%%%%%%%%%%%%%%%% Maurice - 05-06-2008 - Fin écriture du
tableau sur classeur Excel  %%%%%%%%%%%%%%%%%%%%%%
'
'  %%%%%%%%%%%%%%%%%%%%%%%%%%%%%%%%  Maurice 05-06-2008 - Fin affichage sur commande Pause
%%%%%%%%%%%%%%%%%%%%%%%%%%%%%%%%%%%%%%%%%%%
'                                                                                '%
End Sub                                                                            '%

'
'%%%%%%%%%%%%%%%%%%%%%%%%%%%%%  Maurice - 05-06-2008 - Début des affichages en fin de
programme  %%%%%%%%%%%%%%%%%%%%%%%%%%%%%%%%%%%%%%
'                                                                                '%
Public Sub Maurice_AffTbÉlémFinProg()      '                                        '%
'%
   Esp = "Maurice_AffTbÉlémFinProg"        ' Maurice 03-06-2008 - Espionnage                          '%
   Espion                          ' Maurice 03-06-2008 - Espionnage                          '%
   If Option1.Value = True Then GoTo Affiche_Élémentsf        '          - id -                          '%
   'If Option2.Value = True Then GoTo Affiche_LongTermMemorySubf    '          - id -                          '%
'                                                                                '%
'          1) - Tableau des éléments                                               '%
Affiche_Élémentsf:                                                                 '%
   Dim xt, yt, i, j As Integer                                                     '%
   Cls                                                                             '%
   CurrentX = 3100: CurrentY = 6410                                                '%
   Print " Now                                           "                 '%
   CurrentX = 3100: CurrentY = 6410                                                '%
   ForeColor = RGB(255, 0, 0)                                                      '%
   Print "                                          Next"            '%
   ForeColor = RGB(0, 0, 0)                                                        '%
   xt = 1000: yt = 6660                                                            '%
   For i = 1 To Enumber                       ' Attention si Enumber > 32, dépassement capacité         '%
      CurrentX = 900: CurrentY = yt                                                '%
      ForeColor = RGB(0, 0, 255)                                                   '%
      Print i                                                                      '%
      ForeColor = RGB(0, 0, 0)                                                     '%
      For j = 1 To IDPNowNextRange                 ' Attention si NOWNEXTRANGE > 10, dépassement
```

```
capacité          '%
         If j = IDPNowNextRange + 1 Then xt = xt + 800 Else xt = xt + 400  '               - id -                        '%
         If j > IDPNowNextRange Then ForeColor = RGB(255, 0, 0)          '               - id -                        '%
         CurrentX = xt: CurrentY = yt                                                    '%
         Print Element(i, j)                                                      '%
      Next j                                                             '%
   ForeColor = RGB(0, 0, 0)                                                        '%
   xt = 1000: yt = yt + 245                                                      '%
   Next i                                                                '%
'%
   GoTo Fin_Affichage_Fin_Prog                                                          '%
'                                                                       '%
                                                              '%
'                                                                       '%
                                                                 '%
'                                                                       '%
Fin_Affichage_Fin_Prog:                          '                                   '%
End Sub                                                                    '%
'                                                                       '%
'%%%%%%%%%%%%%%%%%%%%%%%%%%  Maurice - 05-06-2008 - Fin des affichages en fin de
programme  %%%%%%%%%%%%%%%%%%%%%%%%%%%%%%%%%%%%
'

 '
' %%%%%%%%%%%%%%%%%%%%%%%%%%%%%%%%%%%%% Maurice - 05-06-2008 - Début Calcul Table
Activity %%%%%%%%%%%%%%%%%%%%%%%%%%%%%%%%%%%%%
Private Sub Maurice_AffTbActivity()             ' %%%% Maurice - 03-06-2008 - affichage du tableau Activity
'%
   Esp = "Maurice_AffTbActivity"               ' Maurice 03-06-2008 - Espionnage                              '%
   Espion                              ' Maurice 03-06-2008 - Espionnage                              '%
'                                                                       '%
' En entrée Table Activity indexée par la ligne la + récente NewLineNumber et le n° de colonne k = 1 à Anumber
'%
'        Le nb de lignes de la table Activity est paramétré par EndOfActivityRegister (150 à ce jour)
'%
' En sortie Tableau 'Activity Register Line' sur feuille principale                              '%
'        La ligne la + récente de l'entrée sera mise en fin du tableau 'Activity Register Line' (ligne 10)              '%
'        On calcule les n° de ligne de la table Activity à positionner en ligne (Ln) du tableau 'Activity Register Line'
:  '%
'              Si NewLineNumber > 9      alors Ltn = NewLineNumber - 9                              '%
'              Si NewLineNumber < ou = 9 alors Ltn = EndOfActivityRegister + NewLineNumber - 9
'%
'                                                                       '%
' Positionnement des n° de ligne de la table Activity                                         '%
   If NewLineNumber > 16 Then                                                        '%
      Ln(0) = NewLineNumber - 16                                                     '%
   Else                                                                  '%
      Ln(0) = EndOfActivityRegister + NewLineNumber - 16                                         '%
   End If                                                                 '%
   For I13 = 1 To 15                                                         '%
      If Ln(I13 - 1) = EndOfActivityRegister Then                                         '%
         Ln(I13) = 1                                                          '%
      Else                                                               '%
         Ln(I13) = Ln(I13 - 1) + 1                                                    '%
      End If                                                              '%
```

```
   Next I13                                                          '%
                                                               '%
' Positionnement des valeurs de la table Activity dans le champ         '%
' 'Activity Register Line' affiché à l'écran                        '%
'                                                         '%
Dim ColMax As Integer        '%
Dim LabelStart As Integer
LabelStart = 0
  If Anumber <= 12 Then ColMax = Anumber Else ColMax = 12      '%
  For I13 = 1 To 16                                        '%
   For I14 = 1 To 12                              '%
   LActivity((I13 - 1) * 12 + (I14 - 1)) = 0        '%
   Next I14                                         '%
   Next I13


        For I13 = (LineNumber - 15) To LineNumber                                       '%
           If I13 < 1 Then GoTo NoLine
           For I14 = 1 To ColMax  '%
              LActivity(LabelStart + (I14 - 1)) = Activity((I13), I14)         '%
           Next I14                                              '%
        LabelStart = LabelStart + 12
        If LabelStart >= (12 * 16) Then LabelStart = 0
NoLine:
        Next I13

'%
' %%%%%%%%%%%%%%%%%%%%%%%%%%%%%%%%%%%%%%% Maurice - 05-06-2008 - Fin Calcul Table
Activity %%%%%%%%%%%%%%%%%%%%%%%%%%%%%%%%%%%%%%%%%%%%
'
'
' %%%%%%%%%%%%%%%%%%%%%%%%%%%%%%%%%%%%%%%%%%%%%%%%%%%%%%%%%% FIN DES PAQUETS
MAURICE %%%%%%%%%%%%%%%%%%%%%%%%%%%%%%%%%%%%%%%%%%%%%%%%%%%%%%%%%%%%%%%%%
'
End Sub

Public Sub ConnectivityExtractSub()
'This gives the number of times each element is cited in the Now fields and in the Next fields of all the Enumber
elements
'Signing is ignored!

For I98 = 1 To Enumber
ConnectivityNow(I98) = 0
ConnectivityNext(I98) = 0
Next I98

'this scoring does not take account of signs
For I98 = 1 To Enumber

   Select Case I98
   Case Is = IDP
   If UseIDP = 0 Then GoTo IDPIsZero
   For J98 = 1 To IDPNowNextRange / 2
   If Element(I98, J98) = 0 Then GoTo ZeroProblemNow
   ConnectivityNow(Element(I98, J98)) = ConnectivityNow(Element(I98, J98)) + 1
```

```
ZeroProblemNow:
   Next J98
   Case Else
   For J98 = KNowNumber(I98) To KNowMin
   ConnectivityNow(Element(I98, J98)) = ConnectivityNow(Element(I98, J98)) + 1
   Next J98
   End Select

   Select Case I98
   Case Is = IDP
   For J98 = (IDPNowNextRange / 2) + 1 To (IDPNowNextRange)
   If Element(I98, J98) = 0 Then GoTo ZeroProblemNext
   ConnectivityNext(Element(I98, J98)) = ConnectivityNext(Element(I98, J98)) + 1
ZeroProblemNext:
   Next J98
   Case Else
   For J98 = KNextMin To KNextNumber(I98)
   ConnectivityNext(Element(I98, J98)) = ConnectivityNext(Element(I98, J98)) + 1
   Next J98
   End Select

IDPIsZero:
Next I98

'Print

End Sub

Public Sub ConnectivityBinSub()
'There are connectivity scores for each element.
'This finds how many times a particular score occurs
'and can bin them if the denominator is set to more than 1

ZeroNow = 0
ZeroNext = 0

I98 = 0
J98 = 0

For I98 = 1 To (NowNextRange * Anumber) 'scores are unlikely to exceed this
BinNow(I98) = 0
BinNext(I98) = 0
Next I98

   For I98 = 1 To Enumber
   BinnedScore = Int((ConnectivityNow(I98)) / 1)
      Select Case BinnedScore
      Case Is <= 0
      ZeroNow = ZeroNow + 1
      Case Else
      BinNow(BinnedScore) = BinNow(BinnedScore) + 1
      End Select

      BinnedScore = Int((ConnectivityNext(I98)) / 1)
      Select Case BinnedScore
```

```
        Case Is <= 0
        ZeroNext = ZeroNext + 1
        Case Else
        BinNext(BinnedScore) = BinNext(BinnedScore) + 1
        End Select


    Next I98

End Sub

Public Sub ConnectivityDisplaySub()

    Picture3.Scale (-5, (Enumber / 10))-((100 + 20), -20) '(left x, top y) - (right x, bottom y)
    Picture3.Cls
    Picture3.FillStyle = 0

    Picture4.Scale (-5, (Enumber / 10))-((100 + 20), -20) '(left x, top y) - (right x, bottom y)
    Picture4.Cls
    Picture4.FillStyle = 0

    Picture5.Scale (-2, (30))-((100 + 20), -2) '(left x, top y) - (right x, bottom y)
    Picture5.Cls
    Picture5.FillStyle = 0

    Picture6.Scale (-2, (30))-((100 + 20), -2) '(left x, top y) - (right x, bottom y)
    Picture6.Cls
    Picture6.FillStyle = 0

    Picture3.Line (0, 1)-(100, 1) 'x-axis normal
    Picture3.Line (0, Enumber / 10)-(0, 0) 'y-axis
    Picture4.Line (0, 1)-(100, 1) 'x-axis
    Picture4.Line (0, Enumber / 10)-(0, 0) 'y-axis
    Picture5.Line (-1, 0)-(90, 0) 'x-axis 'log
    Picture5.Line (-1, 200)-(-1, 0) 'y-axis
    Picture6.Line (-1, 0)-(90, 0) 'x-axis 'log
    Picture6.Line (-1, 200)-(-1, 0) 'y-axis

 For I1 = 1 To (Enumber / 2) Step Enumber / 100
    Picture3.Line (-2, I1)-(0, I1) 'y-axis marks
    Picture4.Line (-2, I1)-(0, I1) 'y-axis marks
    Next I1

    For I1 = 1 To 100 Step 10
    Picture3.Line (I1, 0)-(I1, -3) 'x-axis marks
    Picture4.Line (I1, 0)-(I1, -3) 'x-axis marks
    Next I1

    For I1 = 1 To 100

        Select Case BinNow(I1) 'The radius of the circle is small if the bin is empty
        Case Is = 0
        Picture3.Circle (I1, BinNow(I1)), 1 / 100 'displays bins e.g., 4 lots of sizes (as determined by
ActualMassBinSize)
        'Picture5.Circle (I1, BinNow(I1)), 1 / 100
```

```
        Case Is > 0
        Picture3.Circle (I1, BinNow(I1)), 1 / 2 'displays bins e.g., 4 lots of sizes (as determined by
ActualMassBinSize)
        Picture5.Circle (I1, Log(BinNow(I1))), 1 / 2
        End Select

    Next I1

    For I1 = 1 To 100

        Select Case BinNext(I1) 'The radius of the circle is small if the bin is empty
        Case Is = 0
        Picture4.Circle (I1, BinNext(I1)), 1 / 100 'displays bins e.g., 4 lots of sizes (as determined by
ActualMassBinSize)

        Case Is > 0
        Picture4.Circle (I1, BinNext(I1)), 1 / 2 'displays bins e.g., 4 lots of sizes (as determined by
ActualMassBinSize)
        Picture6.Circle (I1, Log(BinNext(I1))), 1 / 2
        End Select

    Next I1
    'DoEvents
End Sub
```

Program overview CocoIDP827NatComm

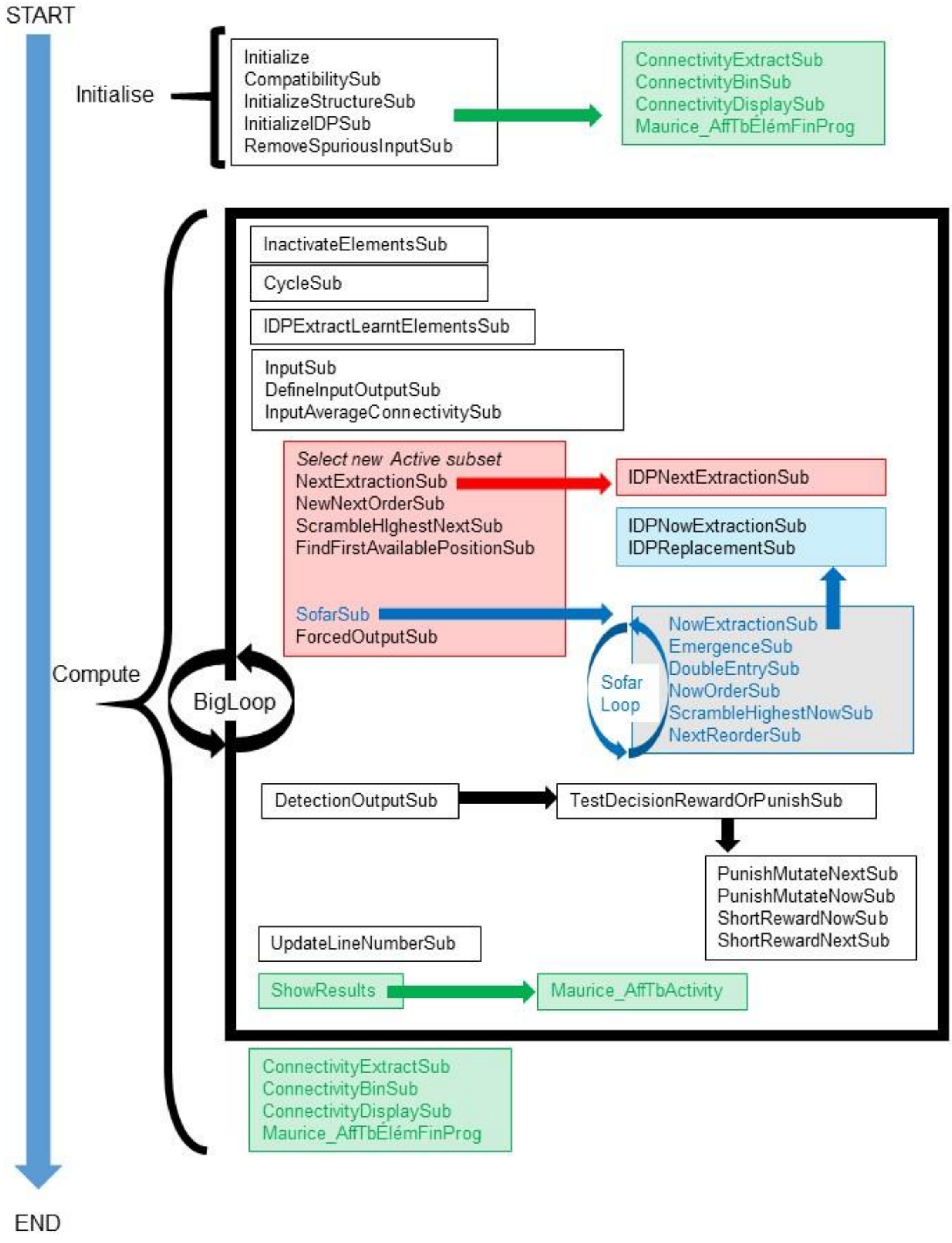

Initialise subroutines

Initialize first calls on CompatibilitySub (below). It then randomly fills the fields of all elements with addresses of other elements (but avoids putting output addresses into the fields of inputs). It makes 1/10 of these connections negative (which reduces the probability that the two elements will be selected together). It calls on InitializeStructureSub, InitializeDownTimeSub and (if UseIDP = 1) InitializeIDPSub. It calls on RemoveSpuriousInputSub, clears the Activity register, sets the LineNumber and NewLineNumber. It loads the Activity register with random elements (but excluding inputs). It sets up inputs and various counters. Finally, it shows some of the elements and their contents with Maurice_AffTbÉlémFinProg (ConnectivityExtractSub, ConnectivityBinSub, ConnectivityDisplaySub are not used here).

CompatibilitySub uses a CompatibilityTable(element1, element2) to allow altering the Now and Next scores obtained for each element so as to take into account that some elements should be selected together and others (like inputs) should not. Here this table is set so that EmergenceSub will give compatibility between all elements except for inputs with inputs.

InitializeStructureSub first calls on ConnectivityExtractSub, ConnectivityBinSub, ConnectivityDisplaySub, Maurice_AffTbÉlémFinProg. Then starting at element-51, it takes element-51 and writes the randomly chosen addresses of elements-50 to -74 into each position in the Now field of element-51; it then repeats this for element-52 and so on until element-86. With a step size of 100, it repeats this sequence starting with element-151 and writing into its field addresses of elements-150 to -174 etc.

InitializeIDPSub The IDP element is termed IDP1 (because there may be two IDP elements). It gives the IDP1 element-10 Now and Next fields of max size (1 to 60 for Now and 61 to 120 for Next)' these fields are filled with addresses 111, 111, 112, 112, 113, 113 …170, 170 (each address is added twice) so the Now range is 111 to 140 and the Next range is 141 to 170   Then 1/10 of all the elements (excluding inputs and outputs) have the IDP1 address written once into their Now and Next fields

RemoveSpuriousInputSub prevents input addresses being generated in the Activity Register that are not coming from the environment. It goes through the fields and removes input addresses. The effect of leaving allowing 'false' inputs to be generated may be worth exploring.

Maurice_AffTbÉlémFinProg is called on by several subroutines to show the first 35 elements and the contents of their fields.

Compute subroutines

InactivateElementsSub reduces the Downtime of each element by 1; then it gives the elements with addresses in the Activity Register the maximum Downtime of 12. The outputs and the IDP1 are given Downtimes of 0. If TimeToSwitch=1 (i.e., IDP2 introduced), then IDP2 can equal 0.

CycleSub If CyclePermission=1 (which is NOT the case here), Coco can insert the address of a new element (the CycleElement) every four lines of the Activity Register' this address is increased by 1 every time it is inserted so, in principle, it allows all the elements to be selected.

IDPExtractLearntElementsSub Between BigLoop 1200 and 1224 (24=2 x Downtime), each element that has had its address in the 24 consecutive lines of the Activity Register (i.e., the successful elements) is recorded as a LearntElement() to be used later.

InputSub uses a counter, InputTotal, to choose the input by calling DefineInputOutputSub and then on InputAverageConnectivitySub

DefineInputOutputSub defines the 5 conditions selected by InputTotal: InputA(1), InputA(2), and InputA(3) give inputs of 1, 2 and 3, respectively, if they are set to 1. OutputA(1), OutputA(2) and OutputA(3) which, if set to 1, require outputs of Enumber-0 (i.e., 1000), Enumber-1, and Enumber-2, respectively as outputs.

InputAverageConnectivitySub gives InputNowScore() and InputNextScore() high scores so that they are forced into the new line of the Activity Register. However, after LoopsTillStorm-200 (i.e., after Loop 400), the inputs are no longer forced into the Activity Register because their scores are set to zero here.

NextExtractionSub extracts how often an address has been referred to in the Next field of the elements in the Activity Register; this gives the score for NextScoreRegister(element address). Outputs are not scored. Signs are taken into account in increasing or decreasing the score. If UseIDP=1 and the element in the Activity Register is IDP1 orIDP2, the routine calls on IDPNextExtractionSub. If TimeToSwitch =1, which can occur at BigLoop=1224 (see above), IDP2 element can be given a high NextScore as can the input element 1 (which is to try to synchronise the learnt sequence).

IDPNextExtractionSub extracts how often an address is present in the Next field of the IDP1 and IDP2 elements; this gives a score to be added to the NextScoreRegister(element address). Signs are taken into account in increasing or decreasing the score. It gives each of the IDPs a NextScoreRegister(IDP) score of 5 (for IDP2, when TimeToSwitch =1).

NewNextOrderSub puts EmergentNextScoreRegister() = NextScoreRegister() (because the EmergenceSub is largely unused) and then it crudely ranks the scores in the NextScoreRegister() as HighestNext(top,1) = address and HighestNext(top,2) = its score, then HighestNext(second to top,1) = its address and HighestNext(second to top,2) = its score, etc.

ScrambleHighestNextSub prevents an artefact which could occur with supposedly independent runs of the program in which the same Nexts could be selected each run despite there being other Nexts with the same score (this is due to NewNextOrderSub putting the lowest addresses on top (e.g. if element-25 and element-37 have the same score, element-25 would always be selected). This routine therefore scrambles the order of elements with the same score.

FindFirstAvailablePositionSub looks at the new line of the Activity Register and, if a position is unfilled, it fills it with the HighestNext. It then updates the HighestNexts (to avoid reselecting the same element), and sets Sofar to point to the last filled position in the new line.

Compute: SofarSub

SofarSub First, it ensures that a second input cannot be loaded. It then calls on NowExtractionSub (which obtains the Now scores of the elements in the new line of the Activity Register). It could – but does not here – introduce noise. It calls on EmergenceSub (which converts the NowScoreRegister() scores into EmergentNowScoreRegister() scores), on DoubleEntrySub (which prevents an element being selected twice) and on NowOrderSub (which uses the EmergentNowScore() scores to order them into the HighestNow scores). It uses ScrambleHighestNowSub to prevent an artefact. It then selects the element from the top of the HighestNext or the top of the HighestNow with the higher score to

be the next member of the Activity Register. It then updates the Sofar pointer and continues looping until the Activity Register is full.

NowExtractionSub First, it takes all the elements that have been selected so far in the new line of the Activity Register and extracts the addresses in their Now fields and scores them in the NowScoreRegister(element address). Outputs are not scored. Signs are taken into account in increasing or decreasing the score. If UseIDP=1 and the element in the Activity Register is IDP1 or IDP2 (if TimeToSwitch=1), the routine calls on IDPNowExtractionSub. If an element is already in the Activity Register, it is given a NowScoreRegister() of 0. InsertNewIDP is used just once at BigLoop=1224 to force in IDP2. If Bigloop0 > 1500 and TimeToSwitch=1 Then NowScoreRegister(IDP2) = 0

IDPNowExtractionSub extracts how often an address has been referred to in the Now field of the IDP1 and IDP2 elements; this gives a score to be added to the NowScoreRegister(element address). Signs are taken into account in increasing or decreasing the score.

IDPReplacementSub creates an IDP2-dependent network mirroring the IDP1-dependent network.

EmergenceSub multiplies the NextScoreRegister() and the NowScoreRegister() by a factor to give the EmergentNextScoreRegister() and the EmergentNowScoreRegister(). This factor is determined via CompatibilitySub. Here, it equals 1 except for the case of inputs with inputs in the NowScoreRegister(input, input) where it equals 0. Note that Compute puts EmergentNextScoreRegister() = NextScoreRegister() (to allow the option of using EmergenceSub)

DoubleEntrySub takes all the elements so far present in the Activity Register and puts all their scores to zero.

NowOrderSub puts EmergentNowScoreRegister() = NowScoreRegister() (because the EmergenceSub is largely unused) and then it crudely ranks the scores in the NowScoreRegister() as HighestNow(top,1) = address and HighestNow(top,2) = its score, then HighestNow(second to top,1) = its address and HighestNow(second to top,2) = its score, etc.

ScrambleHighestNowSub prevents an artefact which could occur with supposedly independent runs of the program in which the same Nows could be selected each run despite there being other Nows with the same score (this is due to NowOrderSub putting the lowest addresses on top (e.g. if element-25 and element-37 have the same score, element-25 would always be selected). This routine therefore scrambles the order of elements with the same score.

NextReorderSub finds the element that has just been selected as the HighestNow in the new as yet unfinished line of the Activity Register and then re-orders the HighestNexts from that entry onwards (thereby preventing an element selected via the NowScores from being re-selected via the NextScores)

Compute continued

ForcedOutputSub generates an output at random that is forced into the Activity Register

DetectionOutputSub counts how many outputs there are in the new line of the Activity Register. It records a failure if there is more than one output. If there is no output at all and if OutputLacking > ForcedOutputProbability then it signals that OutputNeeded = 1 (which will call up ForcedOutputSub). Unless there is no output and no output is needed, it calls TestDecisionRewardOrPunishSub

TestDecisionRewardOrPunishSub DefineInputOutputSub has defined the desired outputs as OutputA(1), OutputA(2) and OutputA(3) as = 1 if we want Enumber, Enumber-1 and Enumber-2, respec-

tively, as outputs. If the program has advanced to the number of loops > LoopsTillStorm, there is neither rewarding nor punishing. To punish, a random line within an input-output sequence is chosen as start and then PunishMutateNextSub and PunishMutateNowSub are called on to act on this and subsequent lines several times according to RepeatRewardPunish (here 5). To reward, all the lines between the input and the output are chosen (plus the previous output line if this was good). Then ShortRewardNextSub and ShortRewardNowSub are called on.

ShortRewardNowSub takes each address in the line of the Activity Register as Element1 and then selects at random Element2; it then writes the address of Element2 into the Now field of Element1 (unless Element1=Element2 or Element1=IDP1 or IDP2). With the present max size of the fields, a bias limit is not used. It avoids overwriting the connections of a previously successful element because it detects whether this element has a field of maximum size. It lengthens the Now field.

ShortRewardNextSub acts like ShortRewardNowSub but takes Element2 from the next line of the Activity Register to the line containing Element1. It lengthens the Next field

PunishMutateNextSub takes each element with an address in the line of the Activity Register and writes a random address into its Next field which it also shortens. IDP1 and IDP2 are exempt.

PunishMutateNowSub takes each element with an address in the line of the Activity Register and writes a random address into its Now field which it also shortens. IDP1 and IDP2 are exempt.

UpdateLineNumberSub increases the number of the line of the Activity Register by 1.

ShowResults displays the highest scoring Now and Next addresses of the elements, the success and fail graph, and calls on Maurice_AffTbActivity

Maurice_AffTbActivity Shows the 16 most recent lines of the Activity Register.

Code for CocoIDP827

'This CocoIDP827NatComm is based on CocoSecondIDP827 is based on CocoInsertionIDP825 and on CocoSecondIDP806 ... is based on 'Coco75 and Coco ANN.' %
' %
' 5 January 2023 %
'Task: 1-1000, 2-999, 3-999, 2-1000, 3-998 ...
%
'%%

'This Coco explores the effect of the introduction of a second IDP
'and its connections on a system that has learnt.
'it extracts the elements (and their fields) in the last 24 (=2*Downtime) lines of the Activity Register
'it then copies these elements into new, unused elements
'(in the sense that they do not appear in the Activity Register)
'and then creates a second IDP with fields equivalent to those of the first IDP
'The addresses in these fields are mainly those of unused elements
'and those corresponding to inputs and outputs are replaced
'The Downtimes of the new set of IDP2 elements are all set to zero
'The second IDP is loaded into the Activity Register and confusion results.
'This confusion is cleared up if the IDP2 is removed AND if an input is given
'to synchronise the system (done in NextExtractionSub).
'There are only TWO modes of functioning:
'1/ Keep the same IDP=10 throughout by changing the number in the
'instruction "Select Case BigLoop0" IN COMPUTE to more than BigLoopMax
'(the max number of loops in the program)
'OR 2/ Bring in a second IDP AND retain the first IDP
'(by setting TimeToSwitch = 1)

'LoopsTillStorm (the number of the time step alias the value of BigLoop0) is set here to 600:
'After time step = 400 (LoopsTillStorm - 200), there is no longer a ForcedOutput or a ForcedInput
'After LoopsTillStorm =600 there is no longer rewarding and punishing
'Between BigLoop0=1200 and BigLoop0=1200 + 24 (2*DownTime)
'the addresses in the Activity Register are used as Learnt Elements in obtaining the second IDP
'After time step = 1500, the Next and Now scores that would select the second IDP
'are set to zero so it disappears (this is done in NextExtractionSub and NowExtractionSub).
'Only IDP1 remains and the phenotype is restored
'The IDP fields cannot be overwritten

Option Explicit ' VB avertira quand un nom de variable créé dans le code ne figure pas dans les déclarations ci-dessous
Option Base 1 ' Affecte la valeur 1 au plus bas niveau d'indice d'un tableau. La seule autre valeur possible est 0

'CHOICES:
Const Test = 0 'put to 0 to avoid preloading a winning pattern; REMEMBER to alter testcompatibilitysub in Initialize
Const MetaLoop = 1 'Allows the program to run for a long time if greater than one
Const NowNextWeighting = 1 'Changes the relative importance of the Now and Next connections when filling the Activity Register
Const CycleLength = 1000 'typically set to 4 to cause cycling every 4 lines of Activity Register
Const ForcedOutputProbability = 1 'OutputLacking goes up by 1 for each line where there is no output (it is

```
reset by detection of output)
'if the counter, OutputLacking, > ForcedOutputProbability Then OutputNeeded
Const NoiseLevel = 0 'for noise to cause insertion of a random element into the ActivityRegister,
            'NoiseLevel has to be greater than R50 which is random between 1 and 20 (hence NoiseLevel has to
be greater than 0
Const MutationThreshold = 90 'mutation threshold is only usedin the PunishMutate routines, range is from 1 to
100,
              'high means few mutations are inserted during punishing MutationNow Int((100 * Rnd) + 1) <
MutationThreshold then no punishing
Const ActivatePunish = 1 'If ActivatePunish = 0 then the punish routines are disabled
Const ActivateReward = 1 'If ActivateReward = 0 then the reward routines are disabled
Const KeepInputSameForThisNumber = 1 'Typically 1 for immediate cycling of inputs
Const NowTooBiasedLimit = 10 'typically = NowNextRange / 2
Const NextTooBiasedLimit = 10
Const RemoveInputByCoco = 0 'if there is a real input, a second artefactual one is removed
Const Enumber = 1000              ' Ces 4 paramètres étant cités dans des Dim
'Const AnumberMax = 100     only for Coco825            ' must be much less than Enumber
Const Anumber = 6 'only for Coco826
Const KNextMin = 9
Const KNowMin = 8
Const KNextMax = 16
Const KNowMax = 1
Const NowNextRange = 16 'divided by two gives the midpoint in Initialize. Also should equal (KNextMax -
KNowMax)+1
Const IDPNowNextRange = 120
Const IDPSparing = 5 'must be less than IDPNowNextRange/2
Const EndOfActivityRegister = 25000 'REMOVE and restore to 150
Const RepeatRewardPunish = 5
Const InputRange = 4
Const OutputRange = 3
Const InputTotalMax = 6
Const OutputRangeMax = 50
Const OutputRangeMin = 100

Const UseIDP = 1 'choose =1 to use the IDP and = 0 not to use it
'Only for Coco825 Const IDP = 10 'choose this element to be highly connected
Const LoopsTillStorm = 600
Const DownTimeNumber = 12 'only for Coco826

'END OF CHOICES


' %%%%%%%%%%%%%%%%%%%%%%%%%%%%%%%%%%%%% Maurice - 05-06-2008 - Déclarations pour séquences
Maurice Affichages %%%%%%%%%%%%%%%%%%%%%%%%%%%%
Dim Susp As Integer                                                    '%
Dim Stopper As Integer                                                 '%
Dim Ctr As Integer                  ' Pour comptage des captures Excel                '%
Dim Save_faite As Integer             ' Pour fichier Coco_Capture                     '%
'Dim xlApp As Excel.Application          ' Déclaration des Objets Excel  - Application           '%
'Dim xlBook As Excel.Workbook            '        - id -       - Classeur               '%
'Dim xlSheet As Excel.Worksheet          '        - id -       - Feuille               '%
Dim Spy, Esp, Esp1, xe, ye As Integer        ' Pour diverses séquences Maurice                '%
'
%%%%%%%%%%%%%%%%%%%%%%%%%%%%%%%%%%%%%%%%%%%%%%%%%%%%%%%%%%%%%%%%%%%%%%%%%%%%%
%%%%%%%%%%%%%%%%%%%%%%%%%%%%%%%%%%%%%%%%%%%%%%%%%%%%%%%%%%%%%%%%%%%%%%%%%%%%%
```

```
%%

Dim IDP2NowReplaced As Integer
Dim TimeToSwitch As Integer
Dim IDP2 As Integer
Dim IDP1 As Integer
Dim InsertNewIDP As Integer
Dim ReplaceThis As Integer
Dim IDP2Element(2 * DownTimeNumber * Anumber, 2) As Integer
Dim ReplacementElement(Enumber, IDPNowNextRange) As Integer
Dim LearntElement(2 * DownTimeNumber * Anumber) As Integer
Dim LearntCounter As Integer
Dim IDP1NowElement As Integer
Dim IDP2NowElement As Integer

Dim InactivateThisNext As Integer
Dim TempNext1 As Integer
Dim TempNext2 As Integer
Dim TempNext3 As Integer
Dim TempNext4 As Integer
Dim TempNext5 As Integer
Dim TempNext6 As Integer
Dim TempNext7 As Integer
Dim TempNext8 As Integer
Dim TempNext9 As Integer
Dim TempNext10 As Integer
Dim TempNow1 As Integer
Dim TempNow2 As Integer
Dim TempNow3 As Integer
Dim TempNow4 As Integer
Dim TempNow5 As Integer
Dim TempNow6 As Integer
Dim TempNow7 As Integer
Dim TempNow8 As Integer
Dim TempNow9 As Integer
Dim TempNow10 As Integer

Dim ThisOne As Integer
Dim Counter As Integer
Dim Rank As Integer
Dim OrderedNext(Enumber, 2) As Integer
Dim NewAnumber As Integer
Dim Downtime(Enumber) As Integer
Dim DowntimeMax(Enumber) As Integer
Dim InsertionScore As Integer
Dim FirstForcedElement As Integer
Dim TimeForStorm As Integer
Dim AnumberCounter As Integer
Dim StormElement As Integer
Dim StormDownTime(Enumber) As Integer
Dim Position As Integer
Dim BinNow(Enumber) As Integer
Dim BinNext(Enumber) As Integer
Dim BinnedScore As Integer
```

```
Dim ZeroNow As Integer
Dim ZeroNext As Integer
Dim ConnectivityNow(Enumber) As Integer
Dim ConnectivityNext(Enumber) As Integer

Dim IDPElement As Integer
Dim KNextNumber(Enumber) As Integer
Dim KNowNumber(Enumber) As Integer
Dim NowNextNumberMidpoint As Integer 'defined as equal to half the NowNextRange in Initialize
Dim IDPNowNextNumberMidpoint As Integer
Dim NowGrowing As Integer
Dim NextGrowing As Integer

Dim MutationFieldSize(Enumber) As Integer

Dim ScrambleLowestNumber As Integer
Dim MarkScrambleLowestStart As Integer
Dim TempScrambleLowestAddress As Integer
Dim TempScrambleLowestScore As Integer

Dim UnusedElement(Enumber, 2) As Integer
Dim LowestUsedElement(Enumber, 2) As Integer
Dim TempUnusedElement(2) As Integer

Dim UsedElement(Enumber, 2) As Integer
Dim HighestUsedElement(Enumber, 2) As Integer
Dim TempUsedElement(2) As Integer

Dim SpareLink(Enumber, Enumber) As Integer
Dim LinkFrom As Integer
Dim LinkTo As Integer
Dim MatrixNow(Enumber, Enumber) As Integer
Dim MatrixNext(Enumber, Enumber) As Integer
Dim TempMatrixNow(Enumber, Enumber) As Integer
Dim TempMatrixNext(Enumber, Enumber) As Integer
Dim ValueOfMatrixNow As Integer
Dim ValueOfMatrixNext As Integer

Dim OutputFrequency(Enumber) As Integer
Dim LineChoice As Integer


Dim CitedElement As Integer
Dim CitingElement As Integer

Dim InputA(InputRange) As Integer
Dim OutputA(OutputRange) As Integer


Dim OutputNowScore(Enumber) As Integer

Dim OutputNeeded As Integer
Dim DontOverwriteNow As Integer
Dim DontOverwriteNext As Integer
Dim DontOverwriteNextOverlap As Integer
```

```
Dim CompatibilityTable(Enumber, Enumber) As Integer
Dim FirstElement As Integer
Dim SecondElement As Integer

Dim CounterForPreviousSuccess As Integer
Dim RewardDecision As Integer
Dim PositionAR1 As Integer
Dim PositionNow1 As Integer
Dim OtherPositionsAR1 As Integer
Dim PositionAR2 As Integer
Dim PositionNext1 As Integer
Dim OtherPositionsAR2 As Integer

Dim PositionAR3 As Integer
Dim PositionNext3 As Integer
Dim OtherPositionsAR3 As Integer

Dim PositionAR4 As Integer
Dim PositionNext4 As Integer
Dim OtherPositionsAR4 As Integer

Dim PositionAR5 As Integer
Dim PositionNext5 As Integer

Dim OutputLacking As Integer
Dim ForcedOutputPosition As Integer
Dim MutationFrequency As Integer
Dim MutationPosition As Integer
Dim MutationElement As Integer
Dim MutatedElement As Integer
Dim MutationNow As Integer
Dim MutationNext As Integer
Dim MutationOverlap As Integer

Dim ConstantInput As Integer
Dim InputTotal As Integer
'for Activity register need to indicate linenumber
Dim LineNumber As Integer
Dim NewLineNumber As Integer
Dim Activity(EndOfActivityRegister, Anumber) As Integer 'AnumberMax for Coco825
Dim Element(Enumber, IDPNowNextRange) As Integer
Dim SignElement(Enumber, Enumber) As Integer
Dim Sign As Integer

'Calculate most frequent elements
Dim NextScoreRegister(Enumber) As Integer
Dim NowScoreRegister(Enumber) As Integer

Dim EmergentNextScoreRegister(Enumber) As Integer
Dim EmergentNowScoreRegister(Enumber) As Integer


Dim HighestNext(Enumber, 2) As Integer
Dim TempHighestNext(2) As Integer
Dim TempHighestNow(2) As Integer
```

```
Dim HighestNow(Enumber, 2) As Integer
'Dim TopNext As Integer
'Dim TopNow As Integer
Dim SavedHighestNext(Enumber, 2) As Integer
Dim SavedHighestNow(Enumber, 2) As Integer


Dim Bigloop0 As Long 'Byte
Dim Bigloop1 As Byte 'unused
Dim A As Integer
Dim E As Integer
Dim K As Integer 'for field within phase element
Dim L As Integer 'for field within phase element
Dim P As Integer 'for phase element
Dim Q As Integer 'for phase element
Dim S As Integer
Dim T As Integer
Dim RandomizeAgain As Integer
Dim InputTally As Integer
Dim JustTestingNow As Integer
Dim JustTestingNext1 As Integer
Dim JustTestingNext2 As Integer
Dim Filler As Integer



'Variables for using the Cycle routine
Dim CycleElement As Integer
Dim CycleStep As Integer
Dim Cycling As Integer
Dim CyclePermission As Integer
'Dim CycleLimit As Integer

'Variables for Scrambling Nows
Dim ScrambleNowNumber As Integer
Dim TempScrambleNowAddress As Integer
Dim TempScrambleNowScore As Integer
Dim MarkScrambleNowStart As Integer

'Variables for Scrambling Nexts
Dim ScrambleNextNumber As Integer
Dim TempScrambleNextAddress As Integer
Dim TempScrambleNextScore As Integer
Dim MarkScrambleNextStart As Integer

'Variables for Running Score (short term memory of successes)
Dim Adaptation As Integer
Dim UseAdaptation As Integer
Dim DeltaRunningScore As Integer
Dim TimeToUseRunningScore As Integer
Dim RunningScorePointer As Integer
Dim RunningScoreTotal1 As Integer
Dim RunningScoreTotal2 As Integer
Dim RunningScoreTotal As Integer
Dim RunningScore(20000) As Integer 'note that this was Enumber but crashes if Enumber is small
```

```
Dim RunningScoreAction As Integer
Dim IncrementRunningScore As Integer
Dim RunningScoreWindow As Integer
Dim RunningScoreLength As Integer
Dim SuccessTableWindow As Integer
Dim PresentResult As Integer
Dim SameResult As Integer

'Variables for neighbourhood connections
Dim MakeLocalConnection As Integer
Dim MakeDistalConnection As Integer
Dim TwoInputs As Integer
Dim MakeLocalNextConnection As Integer
Dim MakeDistalNextConnection As Integer

Dim MakeLocalNowConnection As Integer
Dim MakeDistalNowConnection As Integer
Dim MakeLocalOverlapConnection As Integer
Dim MakeDistalOverlapConnection As Integer

'Victor addition to Coco20atelier 7-6-2008
Dim ModuloRemainder As Integer
Dim WithinLocalNextRangeOutput As Integer
Dim WithinLocalNextRangeInput As Integer
Dim LinkNextOutput  As Integer
Dim LinkNextInput  As Integer
Dim ForceOutput As Integer
Dim DontForceOutput As Integer

'Variables for long term memory
Dim LTMemory(Enumber, Enumber) As Integer

'Victor addition to Coco 20 atelier 7-6-2008
'Variables for phase separation
Dim StuckInLoop As Integer
Dim StuckInDistalLoop As Integer
Dim StuckInLocalLoop As Integer
Dim NewModulusConnection As Integer
Dim ModulusRandomElement As Integer
Dim ModuloReceiver As Integer
Dim ModuloDonor As Integer
Dim ModuloGroup As Integer
'END Victor addition to Coco 20 atelier 7-6-2008

'Victor addition to Coco 20 atelier 28-6-2008
'This is to test for double entry in the Activity Register
Dim DoubleEntry As Integer
'END Victor addition to Coco 20 atelier 28-6-2008

Dim ZeroNSRTotal As Integer
Dim UnusedTopNow As Integer
Dim UnusedTopNext As Integer
Dim InitialTopNow As Integer
Dim InitialTopNext As Integer
```

```
'For rewarding and punishing
Dim RewardInputtoOutput As Integer
Dim PunishInputtoOutput As Integer
Dim RewardOutputtoOutput As Integer
Dim PunishOutputtoOutput As Integer

Dim SumNow As Integer
Dim SumNext As Integer
Dim MeanNow As Integer
Dim MeanNext As Integer
Dim InputNowScore(Enumber) As Integer
Dim InputNextScore(Enumber) As Integer

Dim OldOutputLine As Integer
Dim GoodNewOutput As Integer
Dim GoodOldOutput As Integer

'for phase separation of outputs
Dim TenNowSet(Enumber) As Integer
Dim TwentyNowSet(Enumber) As Integer
Dim TenNextSet(Enumber) As Integer
Dim TwentyNextSet(Enumber) As Integer
Dim PreviousLine As Integer
Dim TenMinusTwentyNow As Integer
Dim TenMinusTwentyNext As Integer

'For recording successes
Dim RecordPointer As Integer
Dim SuccessTable(2000, 3)
Dim OutputResult As Integer
Dim PunishNextLine As Integer
Dim ShortTermMemoryLength As Integer
Dim FullSuccessStory As Integer

'Dim YinYang As Integer
'Dim FailureRecordForYinYang As Integer
'Dim NumberOfYinYangs As Integer
'Dim YinYangCounter As Integer

Dim ExistingNowLink As Integer
Dim ExistingNextLink As Integer

'Each loop counter is used in ONLY one loop

Dim I0 As Integer
Dim I1 As Integer

Dim I2 As Integer
Dim J2 As Integer

Dim I3 As Integer

Dim I4 As Integer
Dim J4 As Integer
Dim I5 As Integer
```

```
Dim I6 As Integer
Dim I7 As Integer
Dim J7 As Integer
Dim I8 As Integer
Dim J8 As Integer
Dim I9 As Integer
Dim J9 As Integer
Dim I10 As Integer
Dim J10 As Integer
Dim I11 As Integer
Dim J11 As Integer
Dim I12 As Integer
Dim I13 As Integer
Dim I14 As Integer
Dim I15 As Integer
Dim I16 As Integer
Dim I17 As Integer
Dim I18 As Integer
Dim J18 As Integer
Dim I19 As Integer
Dim J19 As Integer
Dim I20 As Integer
Dim J20 As Integer
Dim I21 As Integer
Dim I22 As Integer
Dim I23 As Integer
Dim J23 As Integer
Dim I24 As Integer
Dim J24 As Integer
Dim I25 As Integer
Dim J25 As Integer
Dim I26 As Integer
Dim J26 As Integer

Dim I27 As Integer
Dim I28 As Integer
Dim I29 As Integer
Dim I30 As Integer
Dim I31 As Integer
Dim I32 As Integer
Dim I33 As Integer
Dim I34 As Integer
Dim J34 As Integer
Dim I35 As Integer
Dim J35 As Integer
Dim I36 As Integer
Dim J36 As Integer
Dim I37 As Integer
Dim I38 As Integer
Dim I39 As Integer
Dim I40 As Integer
Dim J40 As Integer
Dim I41 As Integer
Dim I42 As Integer
Dim J42 As Integer
```

```
Dim I43 As Integer
Dim J43 As Integer
Dim I44 As Integer
Dim J44 As Integer
Dim I45 As Integer
Dim J45 As Integer
Dim I46 As Integer
Dim J46 As Integer
Dim K46 As Integer
Dim I47 As Integer
Dim J47 As Integer
Dim I48 As Integer
Dim J48 As Integer
Dim I49 As Integer
Dim J49 As Integer
Dim I50 As Integer
Dim J50 As Integer
Dim I51 As Integer
Dim J51 As Integer
Dim I52 As Integer
Dim J52 As Integer
Dim I53 As Integer
Dim I54 As Integer
Dim J54 As Integer
Dim K54 As Integer
Dim I55 As Integer
Dim I56 As Integer
Dim I57 As Integer
Dim I58 As Integer
Dim I59 As Integer
Dim J59 As Integer
Dim I60 As Integer
Dim J60 As Integer
Dim I61 As Integer
Dim I62 As Integer
Dim I63 As Integer
Dim J63 As Integer
Dim K63 As Integer
Dim L63 As Integer

Dim I64 As Integer
Dim J64 As Integer
Dim I65 As Integer
Dim J65 As Integer
Dim K65 As Integer
Dim I66 As Integer
Dim J66 As Integer
Dim K66 As Integer
Dim I67 As Integer
Dim J67 As Integer
Dim I68 As Integer
Dim J68 As Integer
Dim I69 As Integer
Dim I70 As Integer
Dim J70 As Integer
```

```
Dim I71 As Integer
Dim K71 As Integer
Dim I72 As Integer
Dim K72 As Integer
Dim I73 As Integer
Dim I74 As Integer
Dim I75 As Integer
Dim I76 As Integer
Dim J76 As Integer
Dim I77 As Integer
Dim J77 As Integer
Dim I78 As Integer
Dim J78 As Integer
Dim I79 As Integer
Dim J79 As Integer
Dim I80 As Integer
Dim J80 As Integer
Dim I81 As Integer
Dim I82 As Integer
Dim I83 As Integer
Dim I84 As Integer
Dim J84 As Integer
Dim I85 As Integer
Dim I86 As Integer
Dim J86 As Integer
Dim K86 As Integer
Dim I87 As Integer
Dim J87 As Integer
Dim I88 As Integer
Dim J88 As Integer
Dim I89 As Integer
Dim J89 As Integer
Dim I90 As Integer
Dim J90 As Integer
Dim I91 As Integer
Dim J91 As Integer
Dim I92 As Integer
Dim J92 As Integer
Dim K92 As Integer
Dim I93 As Integer
Dim I94 As Integer
Dim J94 As Integer
Dim K94 As Integer
Dim I95 As Integer
Dim J95 As Integer
Dim I96 As Integer
Dim J96 As Integer
Dim I97 As Integer
Dim I98 As Integer
Dim J98 As Integer
Dim I99 As Integer
Dim J99 As Integer
Dim K99 As Integer
Dim M99 As Integer
Dim I100 As Integer
```

```
Dim J100 As Integer
Dim K100 As Integer
Dim L100 As Integer
Dim I101 As Integer
Dim I102 As Integer
Dim I103 As Integer
Dim I104 As Integer
Dim J104 As Integer

'R is for random
Dim R As Integer
Dim R0 As Integer
Dim R1 As Integer
Dim R2 As Integer
Dim R3 As Integer
Dim R4 As Integer
Dim R5 As Integer
Dim R6 As Integer
Dim R7 As Integer
Dim R8 As Integer
Dim R9 As Integer
Dim R10 As Integer
Dim R11 As Integer
Dim R12 As Integer
Dim R13 As Integer
Dim R14 As Integer
Dim R15 As Integer
Dim R16 As Integer
Dim R17 As Integer
Dim R18 As Integer
Dim R19 As Integer
Dim R20 As Integer
Dim R21 As Integer
Dim R22 As Integer
Dim R23 As Integer
Dim R24 As Integer
Dim R25 As Integer
Dim R26 As Integer
Dim R27 As Integer
Dim R28 As Integer
Dim R29 As Integer
Dim R30 As Integer
Dim R31 As Integer
Dim R32 As Integer
Dim R33 As Integer
Dim R34 As Integer
Dim R35 As Integer
Dim R36 As Integer
'Victor addition to Coco 20 atelier 12-6-2008
Dim R37 As Integer
Dim R38 As Integer
Dim R39 As Integer
Dim R40 As Integer
Dim R41 As Integer
Dim R42 As Integer
```

```
Dim R43 As Integer
Dim R44 As Integer
Dim R45 As Integer
Dim R46 As Integer
Dim R47 As Integer
Dim R48 As Integer
Dim R49 As Integer
Dim R50 As Integer
Dim R51 As Integer
Dim R52 As Integer
Dim R53 As Integer
Dim R54 As Integer
Dim R55 As Integer
Dim R56 As Integer
Dim R57 As Integer
Dim R58 As Integer
Dim RandomElement As Integer
Dim R60 As Integer
Dim R61 As Integer
Dim R62 As Integer
Dim R63 As Integer
Dim R64 As Integer
Dim R65 As Integer
Dim R66 As Integer
Dim R67 As Integer
Dim R68 As Integer
Dim R69 As Integer
Dim R70 As Integer
Dim R71 As Integer
Dim R72 As Integer
Dim R73 As Integer
Dim R74 As Integer

'End Victor addition to Coco 20 atelier 12-6-2008

Dim OnlyScoreOneNow As Integer
Dim OnlyScoreOneNext As Integer
Dim AfterInputPosition As Integer
Dim AvailablePosition As Integer
Dim Sofar As Integer
Dim Sofartemp As Integer
Dim RewardNow As Integer
Dim RewardNext As Integer

Dim NumberOfOutputs As Integer
Dim RecordSuccess As Long
Dim RecordFailure As Long
Dim InputNeeded As Integer
Dim OutputLinePlusOne As Integer
Dim InputLine As Integer
Dim Reward As Integer
Dim GrowthResponse As Integer
Dim SporulationResponse As Integer
Dim SubtractionOfLines As Integer
Dim StartLoop As Integer
```

```
Dim EndLoop As Integer
Dim ActRegLine As Integer
Dim TooBiased As Integer
Dim ForcedOutput As Integer
'

Private Sub Anumber_Change()

End Sub

' %%%%%%%%%%%%%%%%%%%%%%%%%%%%%%%%%%%%%%%%%%% Maurice - 24-05-2008 commande
Pause  %%%%%%%%%%%%%%%%%%%%%%%%%%%%%%%%%%%%%%%%%%%%%%%%%%
Private Sub Cmd_Susp_Click()                                                   '%
   Susp = 1 - Susp                                                        '%
End Sub                                                                 '%
'
%%%%%%%%%%%%%%%%%%%%%%%%%%%%%%%%%%%%%%%%%%%%%%%%%%%%%%%%%%%%%%%%%%
%%%%%%%%%%%%%%%%%%%%%%%%%%%%%%%%%%%%%%%%%%%%%%%%%%%%%%%%%%%%%%%%%%
%



' %%%%%%%%%%%%%%%%%%%%%%%%%%%%%%%%%%%%%%%%%%% Maurice - 29-05-2008 commande
Stop  %%%%%%%%%%%%%%%%%%%%%%%%%%%%%%%%%%%%%%%%%%%%%%%%%%%
Private Sub Stop_Coco_Click()                                                  '%
   Stopper = 1 - Stopper                                                   '%
End Sub                                                                 '%
'
%%%%%%%%%%%%%%%%%%%%%%%%%%%%%%%%%%%%%%%%%%%%%%%%%%%%%%%%%%%%%%%%%%
%%%%%%%%%%%%%%%%%%%%%%%%%%%%%%%%%%%%%%%%%%%%%%%%%%%%%%%%%%%%%%%%%%
%

Private Sub Bigloop0Max_Change()
If Bigloop0Max.Text = "" Then
    Bigloop0Max.Text = 1
    Exit Sub
End If
If (Val(Bigloop0Max) < 1) Then Bigloop0Max.Text = 1
If (Val(Bigloop0Max) > 32755) Then Bigloop0Max.Text = 32755
End Sub
'



Private Sub Command1_Click()          ' Command1 = Go     %%%% Maurice 03-06-2008 - Vrai début du
déroulement du code
'
5                                                   ' Repère 5                       '%
   Esp = "Sub Command1_Click"         ' Maurice 24-05-2008 Espionnage                              '%
   Espion                        ' Maurice 24-05-2008 Espionnage                       '%
' %%%%%%%%%%%%%%%%%%%%%%%%%%%  Maurice - 24-05-2008 - Chargement feuille Annexe pour Espion
%%%%%%%%%%%%%%%%%%%%%%%%%%%%%%%%%%%%%%%%
   Load Annexe                   ' Maurice 24-05-2008 - chargement feuille Annexe . . .                 '%
   Annexe.Visible = False            '                 . . . invisible dans le cas général               '%
'
%%%%%%%%%%%%%%%%%%%%%%%%%%%%%%%%%%%%%%%%%%%%%%%%%%%%%%%%%%%%%%%%%%
```

```
%%%%%%%%%%%%%%%%%%%%%%%%%%%%%%%%%%%%%%%%%%%%%%%%%%%%%%%%%%%%%%%%%%%%%%
%%
'
'Show        ' %%%% Maurice 03-06-2008 - inutile, car 'Visible=On' est positionné dans les propriétés de la
feuille      '%
'
Initialize 'If UseInitializeStructure = 1 Then InitializeStructureSub Else Initialize
' %%%%%%%%%%%%%%%%%%%%%%%%%% Maurice - 29-05-2008 - Initialisation à partir de l'écran
%%%%%%%%%%%%%%%%%%%%%%%%%%%%%%%%%%%%%%%%%%%%%%
'   Enumber = Enumber_txt.Text       ' Impossible tant que ces variables sont définies         '%
'   Knumber = Knumber_txt.Text       ' en contantes, ce qui est obligatoire car elles          '%
'   Anumber = Anumber_txt.Text       ' apparaissent ddans des Dim(variable).                   '%
'   DowntimeNumber = DownTime_txt.Text ' Reste à étudier un autre système !                    '%
'
%%%%%%%%%%%%%%%%%%%%%%%%%%%%%%%%%%%%%%%%%%%%%%%%%%%%%%%%%%%%%%%%%%%%%%
%%%%%%%%%%%%%%%%%%%%%%%%%%%%%%%%%%%%%%%%%%%%%%%%%%%%%%%%%%%%%%%%%%%%%%
%%
For I0 = 1 To MetaLoop
Compute
Next I0
   Maurice_AffTbÉlémFinProg        ' %%%% Maurice - 04-06-2008 - vers affichage en fin de run du tableau des
éléments
'
End Sub

Public Sub ShowResults()
   Esp = "Sub ShowResults"         ' Maurice 24-05-2008 - Espionnage
   Espion                          ' Maurice 24-05-2008 - Espionnage
10                                 ' Repère 10
   I12 = 0
   For I12 = 1 To InputRange
'      AInput(I12 - 1) = InputA(I12)
   Next I12
   Label4.Caption = ConstantInput
'
   Maurice_AffTbActivity           ' %%%% Maurice - 04-06-2008 - vers affichage en fin de run du tableau
Activity
'
   I16 = 0
   For I16 = 1 To 11
      LHighestNext1(I16 - 1) = SavedHighestNext(I16, 1)
      LHighestNext2(I16 - 1) = SavedHighestNext(I16, 2)

      LHighestNow1(I16 - 1) = SavedHighestNow(I16 + 1, 1)
      LHighestNow2(I16 - 1) = SavedHighestNow(I16 + 1, 2)
   Next I16
'
'   I17 = 0                        '%%%% Maurice 04-06-2008 - Inutile
   'LTopNow = TopNow               '%%%% Maurice 04-06-2008 - Remise en service du champ sur Feuille
principale  %%%%
   'LTopNext = TopNext             '                 - id -
   LRecordSuccess = RecordSuccess
   LRecordFailure = RecordFailure
'
End Sub
```

```
Public Sub InitializeStructureSub()
'This is no longer an alternative to Initialize

Esp = "CreateStructureSub"            ' Maurice 24-05-2008 - Espionnage
   Espion                     ' Maurice 24-05-2008 - Espionnage
30                                                ' Repère 30

ConnectivityExtractSub
ConnectivityBinSub
ConnectivityDisplaySub
Maurice_AffTbÉlémFinProg

40                                                ' Repère 40
'HERE IS THE NEW STRUCTURE-CREATING BIT
For I99 = (Enumber / 20) To ((Enumber / 20) * 9) Step 100 '((Enumber / 10) + (4 * Anumber)) Step 100
For J99 = I99 + 1 To (I99 + (6 * Anumber)) 'for Enumber=4000, I99 goes from 200 to 1800
'and J99 from 200 to 260, 300 to 360 etc..
For K99 = 1 To KNextMax
R51 = I99 + Int(4 * Anumber * Rnd) 'R51 = 200+(0 to 39)

Select Case K99
Case Is < KNowNumber(J99)
'do nothing
Case Is < KNextNumber(J99)
Element(J99, K99) = R51
Case Else
End Select

Next K99
Next J99
Next I99

ConnectivityExtractSub
ConnectivityBinSub
ConnectivityDisplaySub
Maurice_AffTbÉlémFinProg
End Sub

Public Sub Initialize()
   Esp = "Initialize"           ' Maurice 24-05-2008 - Espionnage
   Espion                     ' Maurice 24-05-2008 - Espionnage
30                                                ' Repère 30

   'NewAnumber = 12
   NowNextNumberMidpoint = NowNextRange / 2
   IDPNowNextNumberMidpoint = IDPNowNextRange / 2

   CompatibilitySub 'TestCompatibilitySub3 ' 'TestCompatibilitySub2 '
   RecordSuccess = 0
   RecordFailure = 0
   InputNeeded = 1

'Randomly fills fields of elements with addresses of other elements
'note that arrays are array(row, column)ie Element(Enumber,Knumber)
```

```
   I18 = 1
   J18 = 1
   For I18 = 1 To Enumber
   R72 = Int(KNowMin * Rnd) + 1 'e.g., int4xRnd +1 = 1,2,3
   KNowNumber(I18) = R72
   R73 = KNowMin + Int((KNextMax - KNowMin) * Rnd) + 1 'e.g., 3+int(6-3)1=3+(0,1,2)+1
   KNextNumber(I18) = R73
      For J18 = R72 To R73 'KNowMin To KNextMin
InitializeAgain:
         Randomize
         R51 = Int((Enumber * Rnd) + 1)
                  Select Case I18 'avoid connecting 1,2 or 3 to outputs
                  Case Is <= InputRange
                  If R51 > (Enumber - OutputRange) Then GoTo InitializeAgain
                  Case Is > (Enumber - OutputRange)
                  If R51 <= InputRange Then GoTo InitializeAgain
                  Case Else

         End Select

         Element(I18, J18) = R51

      Next J18
   Next I18

'Randomly gives signs to connections
For I40 = 1 To Enumber
 For J40 = 1 To Enumber
 SignElement(I40, J40) = 1
         Randomize
         R15 = Int((100 * Rnd) + 1)
         If R15 > 10 Then SignElement(I40, J40) = 1 Else SignElement(I40, J40) = -1
 Next J40
Next I40

40                                                    ' Repère 40

InitializeStructureSub

InitializeIDPSub 'If UseIDP = 1 Then InitializeIDPSub (this is an alternative line if you want to study the absence
of the IDP)

'Removes inputs that occur in the
'initialisation step and that might be confusing
   RemoveSpuriousInputSub

   I21 = 0
'Load Activity Register with zeroes
   For LineNumber = 1 To EndOfActivityRegister
      For I21 = 1 To Anumber
         Activity(LineNumber, I21) = 0
      Next
   Next

   LineNumber = 1
```

```
   NewLineNumber = 2

'set first line of Activity register to random values (but not inputs!)
50                                                    ' Repère 50

ReLoadFirstLine:
   I22 = 0
   For I22 = 1 To Anumber
SetUpCycle:
      Randomize
      Activity(LineNumber, I22) = Int((Enumber * Rnd) + 1)
      If Activity(LineNumber, I22) <= InputRange Then GoTo SetUpCycle
   Next

I10 = 0
   DoubleEntry = 0
    For I10 = 1 To Anumber - 1
      For J10 = I10 + 1 To Anumber
      If Activity(LineNumber, I10) = Activity(LineNumber, J10) Then DoubleEntry = DoubleEntry + 1
      If DoubleEntry = 0 Then GoTo Noproblem
      DoubleEntry = DoubleEntry + 1
Noproblem:
      Next J10
      Next I10

If DoubleEntry > 0 Then GoTo ReLoadFirstLine

'Gives initial CycleElement
CycleElement = 1

'gives initial inputs

I10 = 0
   For I10 = 1 To InputRange
   InputA(I10) = 0
   Next I10

   InputA(1) = 1
   InputTotal = 1
   ConstantInput = 1

   CounterForPreviousSuccess = 1
RunningScoreWindow = 10 'REMOVE AND RESTORE =10
RecordPointer = 1
SuccessTable(RecordPointer, 1) = 1 'inputline
SuccessTable(RecordPointer, 2) = 1 'outputline
SuccessTable(RecordPointer, 3) = 0

ConnectivityExtractSub
ConnectivityBinSub
ConnectivityDisplaySub
Maurice_AffTbÉlémFinProg

End Sub
```

```
Public Sub InitializeIDPSub()
IDP1 = 10
LearntCounter = 1
'Loads the IDP with full fields
   KNowNumber(IDP1) = 1 'KNowMax
   KNextNumber(IDP1) = IDPNowNextRange 'KNextMax for IDP

   IDPElement = IDP1 + 100
   'this fills the Now and Next fields of the IDP
      For I18 = 1 To IDPNowNextRange - 1 Step 2 'KNowMax To KNextMax

         IDPElement = IDPElement + 1
         Element(IDP1, I18) = IDPElement
         Element(IDPElement, KNowMin) = IDP1
         SignElement(IDP1, IDPElement) = 1 'Gives positive signs to connections between IDP and its favourites
         SignElement(IDPElement, IDP1) = 1
         Element(IDP1, I18 + 1) = IDPElement
         Element(IDPElement, KNowMin) = IDP1
         SignElement(IDP1, IDPElement) = 1 'Gives positive signs to connections between IDP and its favourites
         SignElement(IDPElement, IDP1) = 1
      Next I18

'Increase links to the IDP from the other elements
For I18 = InputRange To (Enumber - OutputRange)
      R15 = Int((100 * Rnd) + 1)
         If R15 > 10 Then GoTo MissIDP
         If I18 = IDP1 Then GoTo MissIDP

      For J18 = KNowMin To KNextMin

         Randomize
         'R51 = InputRange + Int(((Enumber - (InputRange + OutputRange)) * Rnd) + 1) 'inputRange=4
outputrange=3; so must be >3 and <4998

         Element(I18, J18) = IDP1
         SignElement(Element(I18, J18), IDP1) = 1
         SignElement(IDP1, Element(I18, J18)) = 1
      Next J18
MissIDP:
   Next I18

ConnectivityExtractSub
ConnectivityBinSub
ConnectivityDisplaySub
Maurice_AffTbÉlémFinProg

End Sub

Public Sub TestEnhanceSub()

I79 = 0
J79 = 0

For I79 = 20 To (Enumber - 10) Step 10
```

```
Element(I79 + 1, 1) = (I79 + 2)
Element(I79 + 2, 1) = (I79 + 3)
Element(I79 + 3, 1) = (I79 + 1)
Element(I79 + 1, 2) = (I79 + 2)
Element(I79 + 2, 2) = (I79 + 3)
Element(I79 + 3, 2) = (I79 + 1)
Element(I79 + 1, 3) = (I79 + 2)
Element(I79 + 2, 3) = (I79 + 3)
Element(I79 + 3, 3) = (I79 + 1)
Next I79


End Sub

Public Sub YinYangSub()
'YinYang with odds and evens

If NumberOfYinYangs > 1 Then GoTo MissYinYang
Select Case YinYang

Case Is = 6
For I63 = 4 To Enumber Step 2
For J63 = 4 To Enumber Step 2
CompatibilityTable(I63, J63) = 2 '
Next J63
Next I63

Case Is = 7
For I63 = 5 To Enumber Step 2
For J63 = 5 To Enumber Step 2
CompatibilityTable(I63, J63) = 2 '
Next J63
Next I63

End Select

'makes full incompatibilities between inputs
For I63 = 1 To 3
For J63 = (I63 + 1) To 3
CompatibilityTable(I63, J63) = 0 ' was 1 remove
CompatibilityTable(J63, I63) = 0 ' was 1 remove
Next J63
Next I63

'makes full compatibilities between inputs and elements
For I63 = 1 To Enumber
CompatibilityTable(I63, 1) = 1
CompatibilityTable(1, I63) = 1
CompatibilityTable(I63, 2) = 1
CompatibilityTable(2, I63) = 1
CompatibilityTable(I63, 3) = 1
CompatibilityTable(3, I63) = 1
Next I63
```

```
'makes full incompatibilities between inputs
For I63 = 1 To 3
For J63 = (I63 + 1) To 3
CompatibilityTable(I63, J63) = 0 ' was 1 remove
CompatibilityTable(J63, I63) = 0 ' was 1 remove
Next J63
Next I63

'makes full compatibilities between outputs and elements
For I63 = 1 To Enumber
For J63 = 1 To OutputRange
CompatibilityTable(I63, (1 + Enumber - OutputRange)) = 1
CompatibilityTable((1 + Enumber - OutputRange), I63) = 1
Next J63
Next I63

'Diagnonal selfing compatibilities
For I63 = 1 To Enumber
CompatibilityTable(I63, I63) = 1 'was 10
Next I63

MissYinYang:

End Sub

Public Sub ResetYinYangSub()
For I63 = 1 To Enumber
For J63 = 1 To Enumber
CompatibilityTable(I63, J63) = 1
Next J63
Next I63

'makes full incompatibilities between inputs
For I63 = 1 To 3
For J63 = (I63 + 1) To 3
CompatibilityTable(I63, J63) = 0 ' was 1 remove
CompatibilityTable(J63, I63) = 0 ' was 1 remove
Next J63
Next I63
End Sub
Public Sub CompatibilitySub()
   'RESTORE THIS WHEN TESTING FINISHED

   Esp = "Compatibility"            ' Maurice 24-05-2008 - Espionnage
   Espion                     ' Maurice 24-05-2008 - Espionnage

'could make random compatibility groups between 1 and 10 for all elements except for inputs
'but instead makes full compatibilities
For I63 = 1 To Enumber
For J63 = (I63 + 1) To (Enumber - 1)
Randomize
CompatibilityTable(I63, J63) = 1 'was Int((10 * Rnd) + 1)
CompatibilityTable(J63, I63) = 1 ' was = CompatibilityTable(I63, J63)
Next J63
Next I63
```

```
'makes full compatibilities for inputs and all other elements upto first half of Enumber
For I63 = 1 To InputRange
For J63 = (InputRange + 1) To Enumber / 2
CompatibilityTable(I63, J63) = 1  'was 10
CompatibilityTable(J63, I63) = 1 'was 10
Next J63
Next I63

'makes full compatibilies for inputs with second half of elements (but could make this incompatibilities
For I63 = 1 To InputRange
For J63 = ((Enumber / 2) + 1) To Enumber
CompatibilityTable(I63, J63) = 1
CompatibilityTable(J63, I63) = 1
Next J63
Next I63

'makes full incompatibilities between inputs
For I63 = 1 To InputRange
For J63 = (I63 + 1) To InputRange
CompatibilityTable(I63, J63) = 0 ' was 1 remove
CompatibilityTable(J63, I63) = 0 ' was 1 remove
Next J63
Next I63

'Diagnonal selfing compatibilities
For I63 = 1 To Enumber
CompatibilityTable(I63, I63) = 1 'was 10
Next I63

End Sub

Public Sub TestSetUpWinningSeries2()

End Sub


Public Sub Compute() '4/02/2011

  Esp = "Compute"            ' Maurice 24-05-2008 - Espionnage
  Espion                     ' Maurice 24-05-2008 - Espionnage
'this Bigloop comprises most of the program; it starts by loading inputs

'   Dim Fin_affiche As String                04-06-2008
  Picture1.Line (0, 0)-(0, 0)

  EnumberLabel.Text = Enumber
  KnumberLabel.Text = NowNextRange
  AnumberLabel.Text = Anumber

  For Bigloop0 = 1 To Bigloop0Max '0

  LBoucle = Bigloop0

    I31 = 0
```

```
      For I31 = 1 To Anumber
      Activity(NewLineNumber, I31) = 0
      Next I31

   InactivateElementsSub 'If DowntimeNumberRange > 0 Then InactivateElementsSub

   If CyclePermission = 1 Then CycleSub

         Select Case Bigloop0
         Case Is < 1200
         'do nothing
         Case Is < 1200 + (2 * DownTimeNumber)
         IDPExtractLearntElementsSub 'This is done a DownTime number of times.
         Case Is = 1200 + (2 * DownTimeNumber)
         InsertNewIDP = 1 'Now there must be an input of 'IDP2'
         TimeToSwitch = 1 'This is where we decide whether to have one IDP (=0) or two IDPs at the same time
(=1)
         Case Else
         End Select

         Select Case InputNeeded
         Case Is = 0
         Case Is = 1
         Randomize
         R66 = R66 + 1 'Int((4 * Rnd) + 1) doesn't do anything in its present form
         If R66 = 1 Then InputSub
         If R66 = 1 Then R66 = 0
         End Select

   'If InputNeeded = 1 Then InputSub 'along with InputAverageConnectivitySub puts InputNextScore(1 0r 2 0r 3)
to a high value

   NextExtractionSub 'puts Next scores from elements in Activity(Linenumber) into the NextScoreRegister
   'EmergenceSub is not used here even if there is an input or a cyclic element and the first HighestNext has yet
to be loaded
   'and Activity(NewLine, 1) = 0.

   'The following is because EmergentNextScoreRegister has not been filled in EmergenceSub
     I4 = 0
      For I4 = 1 To Enumber
      EmergentNextScoreRegister(I4) = NextScoreRegister(I4) 'EmergentNextScoreRegister is used by the
following NextOrderSub
      Next I4

   NewNextOrderSub 'NextOrderSub     'NewNextOrderSub
   ScrambleHighestNextSub
   FindFirstAvailablePositionSub 'FINDS FIRST AVAILABLE POSITION IN ACTIVITY REGISTER, loads HighestNext as
Sofar

SavedHighestNext(1, 1) = HighestNext(1, 1)
SavedHighestNext(1, 2) = HighestNext(1, 2)

70                                        ' Repère 70
```

```
'Only for Coco825
SofarSub 'LOAD ACTIVITY REGISTER USING NOW/NEXT COMPETITION BY OBTAINING HIGHEST NOW EACH
CYCLE
'CHECK THAT THERE ARE NO ZEROES IN ACTIVITY REGISTER ELSE WILL GET AN ERROR FROM element (A1, A2)
SINCE A2 WILL EQUAL ZERO'

120                                             ' Repère 120

If Bigloop0 < (LoopsTillStorm - 200) And OutputNeeded = 1 Then ForcedOutputSub '

      Select Case UseIDP
      Case Is = 0
        Select Case TimeForStorm
        Case Is = 0
        'If Bigloop0 >= LoopsTillStorm And InputTotal = 5 Then ForcedElementsInsertionSub '
        If Bigloop0 >= LoopsTillStorm And InputTotal = 5 Then TimeForStorm = 1
        Case Else
        'do nothing
        End Select

      Case Is = 1
        Select Case TimeForStorm
        Case Is = 0
        'If Bigloop0 >= LoopsTillStorm And InputTotal = 5 Then ForcedElementsInsertionSub '
        If Bigloop0 >= LoopsTillStorm And InputTotal = 5 Then TimeForStorm = 1
        Case Else
       'do nothing
        End Select
      End Select


'eliminates a second input from Coco
If RemoveInputByCoco = 1 Then RemoveInputGenerationByCocoSub

'sets variables to zero
  Reward = 0
  RewardNow = 0
  RewardNext = 0

If InputNeeded = 0 Then DetectionOutputSub 'detects the presence, nature and number of outputs

UpdateLineNumberSub
140                                             ' Repère 140
'This is to avoid dividing by zeroes
  If RecordSuccess = 0 Then RecordSuccess = 1
  If RecordFailure = 0 Then RecordFailure = 1

  Picture1.Line -((Bigloop0 / Bigloop0Max) * 100, RecordSuccess / (RecordSuccess + RecordFailure) * 100),
QBColor(0)
145
  ShowResults
  DoEvents
Next Bigloop0    'end of bigloop0        ' Maurice 05-06-2008 - Pour s'assurer que le Next est bien attribué à
Bigloop0 - Repère Paquet
```

```
ConnectivityExtractSub
ConnectivityBinSub
ConnectivityDisplaySub
Maurice_AffTbÉlémFinProg

End Sub

Public Sub RemoveZeroAndDoubleEntrySub()
I10 = 0
   For I10 = 1 To Anumber
      Randomize
      If Activity(NewLineNumber, I10) = 0 Then Activity(NewLineNumber, I10) = Int((Enumber * Rnd) + 1)
   Next I10

RerunDoubleEntry:

   I10 = 0
   DoubleEntry = 0
    For I10 = 1 To Anumber - 1
      For J10 = I10 + 1 To Anumber
      If Activity(NewLineNumber, I10) = Activity(NewLineNumber, J10) Then DoubleEntry = DoubleEntry + 1
      If DoubleEntry = 0 Then GoTo Noproblem
      Activity(NewLineNumber, I10) = Int((Enumber * Rnd) + 1)

      DoubleEntry = DoubleEntry + 10

Noproblem:
      Next J10
      Next I10

      If DoubleEntry > 0 Then GoTo RerunDoubleEntry

End Sub
Public Sub FindFirstAvailablePositionSub()

'after this routine, Sofar points to a filled position
Sofar = 0
   I5 = 0
   For I5 = 1 To Anumber
      AvailablePosition = 0
      If Activity(NewLineNumber, I5) = 0 Then AvailablePosition = 1
      'By inactivating the following line, I take control away from the previous AR Line
      If AvailablePosition = 1 Then Activity(NewLineNumber, I5) = HighestNext(1, 1) 'There should ALWAYS be a
HighestNext to insert
      If AvailablePosition = 1 Then Rank = 2 'this is the pointer for the Nexts
      If AvailablePosition = 1 Then Sofar = I5
      If AvailablePosition = 1 Then I5 = Anumber
   Next I5

End Sub

Public Sub SofarSub()

'Sofar points to a filled space in the Activity Register
```

```
   For Sofartemp = Sofar To (Anumber - 1)

Select Case Activity(NewLineNumber, Sofar)

Case Is <= InputRange 'The element is an input

 I62 = 0
   For I62 = 1 To InputRange
   InputNowScore(I62) = 0
   InputNextScore(I62) = 0
   Downtime(I62) = DowntimeMax(I62)
   Next I62

Case Else

End Select


NowExtractionSub

R50 = Int((20 * Rnd) + 1)
If NoiseLevel < R50 Then GoTo MakeNoNoise

         Randomize
         R50 = Int(RunningScoreWindow * Rnd) - 1 'make this - 1 if want to avoid all Noise after successful
learning

         If (RunningScoreTotal / RunningScoreWindow) > (R50 / RunningScoreWindow) Then GoTo MakeNoNoise

NoiseSub

MakeNoNoise:

EmergenceSub

DoubleEntrySub

NowOrderSub

ScrambleHighestNowSub 'This is to avoid the artefact in which Nows with the same score always have the
lowest address on top

SavedHighestNext(Sofartemp + 1, 1) = HighestNext(Rank, 1)
SavedHighestNext(Sofartemp + 1, 2) = HighestNext(Rank, 2)
SavedHighestNow(Sofartemp + 1, 1) = HighestNow(1, 1)
SavedHighestNow(Sofartemp + 1, 2) = HighestNow(1, 2)

'ShowResults 'enable this and disable it in SofarSub if you want to follow loading the ActivityRegister step by
step


110                                          ' Repère 110
   Select Case HighestNext(Rank, 2)
   Case Is <= (NowNextWeighting * HighestNow(1, 2))
   Activity(NewLineNumber, Sofar + 1) = HighestNow(1, 1) 'Inactivation will be done by NowExtractionSub
```

```
   InactivateThisNext = HighestNow(1, 1)
   NextReorderSub
   Case Else
   Activity(NewLineNumber, Sofar + 1) = HighestNext(Rank, 1)
   Rank = Rank + 1
   End Select

'in case of a bizarre problem:
If Activity(NewLineNumber, Sofar + 1) = 0 Then Activity(NewLineNumber, Sofar + 1) = Int(Enumber * Rnd) + 1

   Sofar = Sofar + 1

'End of Sofartemp loop
Next Sofartemp

End Sub

Public Sub DoubleEntrySub()
I9 = 0
J9 = 0

   For I9 = 1 To Enumber
      For J9 = 1 To Sofar
         If I9 = Activity(NewLineNumber, J9) Then NowScoreRegister(I9) = 0
         If I9 = Activity(NewLineNumber, J9) Then NextScoreRegister(I9) = 0
         If I9 = Activity(NewLineNumber, J9) Then EmergentNowScoreRegister(I9) = 0
         If I9 = Activity(NewLineNumber, J9) Then EmergentNextScoreRegister(I9) = 0
      Next J9

   Next I9
End Sub


Public Sub DetectionOutputSub()
   NumberOfOutputs = 0

I11 = 0
   For I11 = 1 To Anumber
   For J11 = 1 To OutputRange
      If Activity(NewLineNumber, I11) = (1 + Enumber) - J11 Then NumberOfOutputs = NumberOfOutputs + 1
      Next J11
   Next I11

I11 = 0

   OutputLinePlusOne = NewLineNumber + 1
   If OutputLinePlusOne = EndOfActivityRegister + 1 Then OutputLinePlusOne = 1
'InputLine has been set by the InputSubroutine
130                                         ' Repère 130

   Select Case NumberOfOutputs

   Case 0
   'There is no output (NumberOfOutputs = 0) and there are 2 possibilities:
```

```
    Select Case OutputLinePlusOne

    Case InputLine
      '1/ The Activity Register is full and OutputLinePlusOne = InputLine
      InputNeeded = 1
      RecordFailure = RecordFailure + 1

    Case Else
      '2/ The Activity Register is not full and OutputLinePlusOne <> InputLine

      InputNeeded = 0
        Randomize
  OutputLacking = OutputLacking + 1

  If OutputLacking > ForcedOutputProbability Then OutputNeeded = 1 '

    End Select

  Case 1
  'There is a single output - but is it the desired one?
   TestDecisionRewardOrPunishSub 'remove
   InputNeeded = 1
    OutputLacking = 0

  Case Else
  'There is more than one output so there must be punishment
    TestDecisionRewardOrPunishSub 'remove
    InputNeeded = 1
    OutputLacking = 0 'remove

    DoEvents
  End Select
End Sub

Public Sub ForcedOutputSub()

DontForceOutput = 0 'new routine to remove

For I78 = 1 To Anumber
For J78 = 1 To OutputRange
If Activity(NewLineNumber, I78) = (1 + Enumber - J78) Then DontForceOutput = 1
Next J78
Next I78

If DontForceOutput = 1 Then GoTo MissForcingOutput

Randomize
R54 = Int(OutputRange * Rnd)
Activity(NewLineNumber, Anumber) = Enumber - R54 'CHECK THIS!!!!!!!!!!

MissForcingOutput:

OutputNeeded = 0

End Sub
```

```
Public Sub IDPNextExtractionSub()
   For J2 = (IDPNowNextNumberMidpoint + 1) To IDPNowNextRange
      Randomize
      If Element(A, J2) = 0 Then Element(A, J2) = Int((Enumber * Rnd) + 1)

If Downtime(Element(A, J2)) > 0 Then GoTo NoScoringOfOutputNexts3

'Stop cyclic element being loaded twice
If Element(A, J2) = CycleElement Then GoTo NoScoringOfOutputNexts3
S = Element(A, J2)

Sign = SignElement(A, Element(A, J2))
If Sign >= 0 Then NextScoreRegister(S) = NextScoreRegister(S) + 1
If Sign < 0 Then NextScoreRegister(S) = NextScoreRegister(S) - 1

NoScoringOfOutputNexts3:

   Next J2

End Sub
Public Sub NextExtractionSub()
   Esp = "NextExtractionSub"
   Espion

'Extracts how often an address has been referred to in the Next field of the elements active in the Activity Register.
'linenumber selects 'the line in the Activity Register and I2 selects the element within it; J+Knumber selects the elements within its Next field

'I1 Sets NextScoreRegister and HighestNext to zero
I1 = 0
   For I1 = 1 To Enumber
      NextScoreRegister(I1) = 0
      HighestNext(I1, 1) = 0
      HighestNext(I1, 2) = 0
   Next I1

I2 = 0
J2 = 0
S = 0
For I2 = 1 To Anumber
   A = Activity(LineNumber, I2)

  'No scoring from outputs
        If A > Enumber - OutputRange Then GoTo NoScoringOfOutputNexts1

         If IDP2NowReplaced = 0 Then GoTo IDP2NotReplacedYet
         Select Case TimeToSwitch '
         Case Is = 0
         If A = IDP1 Then IDPNextExtractionSub
         If A = IDP1 Then GoTo DoneIDPNextExtraction
         Case Is = 1
         Downtime(IDP1) = 0
         If A = IDP1 Then IDPNextExtractionSub
```

```
        If A = IDP1 Then GoTo DoneIDPNextExtraction
        Downtime(IDP2) = 0
        If A = IDP2 Then IDPNextExtractionSub
        If A = IDP2 Then GoTo DoneIDPNextExtraction
        Case Else
        End Select
IDP2NotReplacedYet:

  If A = 0 Then GoTo ThisIsAnumberIncreaseProblem
  For J2 = (NowNextNumberMidpoint + 1) To KNextNumber(A)
    Randomize
    If Element(A, J2) = 0 Then Element(A, J2) = Int((Enumber * Rnd) + 1)

If Downtime(Element(A, J2)) > 0 Then GoTo NoScoringOfOutputNexts2

'Stop cyclic element being loaded twice
If Element(A, J2) = CycleElement Then GoTo NoScoringOfOutputNexts2
S = Element(A, J2)

Sign = SignElement(A, Element(A, J2))
If Sign >= 0 Then NextScoreRegister(S) = NextScoreRegister(S) + 1
If Sign < 0 Then NextScoreRegister(S) = NextScoreRegister(S) - 1

NoScoringOfOutputNexts2:

  Next J2

NoScoringOfOutputNexts1:
DoneIDPNextExtraction:

Next I2
ThisIsAnumberIncreaseProblem:

I61 = 0
For I61 = 1 To InputRange
If InputA(I61) = 1 Then NextScoreRegister(I61) = NextScoreRegister(I61) + InputNextScore(I61)
'If InputA(I61) = 1 Then Downtime(I61) = 0 '1 'DowntimeMax(I61)
Next I61

For I77 = 1 To Enumber
If Downtime(I77) > 0 Then NextScoreRegister(I77) = 0
Next I77

        Select Case TimeToSwitch
        Case Is = 0
        NextScoreRegister(IDP1) = 5
        Case Is = 1
        NextScoreRegister(IDP1) = 5
        If IDP2NowReplaced = 0 Then GoTo IDP2NotReady
        If Bigloop0 < 1500 Then NextScoreRegister(IDP2) = 5 Else NextScoreRegister(IDP2) = 0
        If Bigloop0 = 1520 Then NextScoreRegister(1) = 100
        If Bigloop0 = 1520 Then NextScoreRegister(IDP1) = 10
        Case Else
IDP2NotReady:
        End Select
```

```
End Sub


Public Sub IDPNowExtractionSub()
For J7 = 1 To IDPNowNextNumberMidpoint
      If Element(A, J7) = 0 Then Element(A, J7) = Int((Enumber * Rnd) + 1)
      If Downtime(Element(A, J7)) > 0 Then GoTo NoScoringOfIDPNows
      S = Element(A, J7)
      Sign = SignElement(A, S)
If Sign > 0 Then NowScoreRegister(S) = NowScoreRegister(S) + 1
If Sign < 0 Then NowScoreRegister(S) = NowScoreRegister(S) - 1

NoScoringOfIDPNows:
  Next J7
End Sub
Public Sub NowExtractionSub()
'FIND HIGHEST NOW VALUES FROM ELEMENTS IN ACTIVITY AND LOAD INTO NowScoreRegister

'Sets NowScoreRegister and Highest Now to zero
90                                                ' Repère 90
      I6 = 0
      For I6 = 1 To Enumber
         NowScoreRegister(I6) = 0
         HighestNow(I6, 1) = 0
         HighestNow(I6, 2) = 0
      Next I6

'(2)Extracts how often an address has been referred to in the Now field
'of the elements active in the Activity Register

I7 = 0
J7 = 0
For I7 = 1 To Sofar
   A = Activity(NewLineNumber, I7)
    'Do not score Output fields
      If A > Enumber - OutputRange Then GoTo NoScoringOfOutputNows

      Randomize
      If A = 0 Then A = Int((Enumber * Rnd) + 1)

         Select Case TimeToSwitch
         Case Is = 0
         If A = IDP1 Then IDPNowExtractionSub
         If A = IDP1 Then GoTo DoneIDPNowExtraction
         Case Is = 1
         Downtime(IDP1) = 0
         If A = IDP1 Then IDPNowExtractionSub
         If A = IDP1 Then GoTo DoneIDPNowExtraction
         If A = IDP2 And IDP2NowReplaced = 1 Then Downtime(IDP2) = 0
         If A = IDP2 Then IDPNowExtractionSub
         If A = IDP2 Then GoTo DoneIDPNowExtraction
         Case Else
         End Select
```

```
      For J7 = (KNowNumber(A)) To NowNextNumberMidpoint
      If Element(A, J7) = 0 Then Element(A, J7) = Int((Enumber * Rnd) + 1)

      If Downtime(Element(A, J7)) > 0 Then GoTo NoScoringOfNows

      S = Element(A, J7)
      Sign = SignElement(A, S)
If Sign > 0 Then NowScoreRegister(S) = NowScoreRegister(S) + 1
If Sign < 0 Then NowScoreRegister(S) = NowScoreRegister(S) - 1


NoScoringOfNows:

  Next J7
NoScoringOfOutputNows:
NoScoringOfInputNows:
DoneIDPNowExtraction:

Next I7

'Ensure that a Now that is ALREADY in the ActivityRegister does not get scored (and perhaps loaded again)
   I76 = 0
   For I76 = 1 To Sofar
    A = Activity(NewLineNumber, I76)
   If A = 0 Then GoTo DontResetNowScore
   NowScoreRegister(A) = 0
   NextScoreRegister(A) = 0
DontResetNowScore:
   Next I76

   For I76 = 1 To Enumber
   If Downtime(I76) > 0 Then NowScoreRegister(I76) = 0
   Next I76

      Select Case InsertNewIDP
      Case Is = 0
      'do nothing (note that IDPExtractNLearntElementsSub has already been used
      Case Is = 1
      If InputA(1) = 1 Then IDPReplacementSub 'gives the newIDP a NowScore =300
      If InputA(1) = 1 Then NowScoreRegister(IDP2) = 300
      If InputA(1) = 1 Then InsertNewIDP = 2
      Case Is < (Anumber) 'keep loading the
      IDPInsertNewLineSub
      InsertNewIDP = InsertNewIDP + 1
      Case Else
      End Select

         Select Case TimeToSwitch
         Case Is = 0
         Case Is = 1
         If Bigloop0 > 1500 Then NowScoreRegister(IDP2) = 0
          End Select
End Sub

Public Sub IDPInsertNewLineSub() 'changed from Coco825
```

```
'Seems to be preparing to insert the new IDP into the New line via NowExtractionSub
'IDP2 =300 and has become element 1
'it is going through the addresses in element(1)'s Now field
'Input(A)=1 and element(1) has been loaded into first place in the NewLine of the Activity register
For J99 = KNowNumber(1) To IDPNowNextNumberMidpoint
NowScoreRegister(Element(IDP2, J99)) = 100
Next J99

End Sub

Public Sub NewNextOrderSub()

For I4 = 1 To Enumber
OrderedNext(I4, 2) = 0
Next I4

   For I4 = 1 To Enumber
   HighestNext(I4, 1) = I4
   HighestNext(I4, 2) = EmergentNextScoreRegister(I4) 'These are the scores modified by EmergenceSub
   Next I4


For Rank = 1 To Anumber
For I4 = 1 To Enumber
If OrderedNext(Rank, 2) > HighestNext(I4, 2) Then GoTo MissOrder
OrderedNext(Rank, 2) = HighestNext(I4, 2)
OrderedNext(Rank, 1) = HighestNext(I4, 1)
ThisOne = HighestNext(I4, 1)
MissOrder:
Next I4

HighestNext(ThisOne, 2) = 0
Next Rank

For Rank = 1 To Anumber
HighestNext(Rank, 1) = OrderedNext(Rank, 1)
HighestNext(Rank, 2) = OrderedNext(Rank, 2)
Next Rank


End Sub

Public Sub NextReorderSub()
'called on by SofarSub
'first find the element that has appeared in the new as yet unfinished Activity line
'then re-order the HighestNexts from that entry onwards

For I104 = 1 To (Anumber - 1) '
If HighestNext(I104, 1) <> InactivateThisNext Then GoTo NotFoundIt
   For J104 = I104 To Anumber
   HighestNext(J104, 1) = HighestNext(J104 + 1, 1)
   HighestNext(J104, 2) = HighestNext(J104 + 1, 2)
   Next J104
I104 = Anumber
NotFoundIt:
```

```
Next I104
End Sub

Public Sub NextOrderSub()

    Esp = "NextOrderSub"                    ' Maurice 24-05-2008 - Espionnage
  Espion
'(3)use NextScoreRegister so most frequent are ordered in HighestNext

80                                                ' Repère 80

  TempHighestNext(1) = 0
  TempHighestNext(2) = 0

  I4 = 0
  For I4 = 1 To Enumber
  HighestNext(I4, 1) = I4
  HighestNext(I4, 2) = EmergentNextScoreRegister(I4) 'These are the scores modified by EmergenceSub
  Next I4

I4 = 0
J4 = 0
For I4 = 1 To (Enumber - 1)
For J4 = (I4 + 1) To Enumber
If HighestNext(I4, 2) >= HighestNext(J4, 2) Then GoTo KeepHighestNext
TempHighestNext(1) = HighestNext(I4, 1)
TempHighestNext(2) = HighestNext(I4, 2)
HighestNext(I4, 1) = HighestNext(J4, 1)
HighestNext(I4, 2) = HighestNext(J4, 2)
HighestNext(J4, 1) = TempHighestNext(1)
HighestNext(J4, 2) = TempHighestNext(2)

KeepHighestNext:
Next J4
Next I4

End Sub

Public Sub EmergenceSub()

For I64 = 1 To Sofar
For J64 = 1 To Enumber
If Activity(NewLineNumber, I64) = 0 Then GoTo ZeroAddressError 'this is an error condition

Select Case CompatibilityTable(Activity(NewLineNumber, I64), J64)

Case Is < 1
EmergentNowScoreRegister(J64) = NowScoreRegister(J64) * 0 'remove l
EmergentNextScoreRegister(J64) = NextScoreRegister(J64) * 1 'remove 1

Case Is = 1
EmergentNowScoreRegister(J64) = NowScoreRegister(J64) * 1 'was * 1 / 2
EmergentNextScoreRegister(J64) = NextScoreRegister(J64) * 1 'was * 1 / 2
Case Is = 2
EmergentNowScoreRegister(J64) = NowScoreRegister(J64) * 10 'was * 1 / 2
```

```
EmergentNextScoreRegister(J64) = NextScoreRegister(J64) * 10 'was * 1  / 2
Case Is = 3
EmergentNowScoreRegister(J64) = NowScoreRegister(J64) * 1 'was * 1 / 2
EmergentNextScoreRegister(J64) = NextScoreRegister(J64) * 1 'was * 1  / 2
Case Is = 4
EmergentNowScoreRegister(J64) = NowScoreRegister(J64) * 1 'was * 1 / 2
EmergentNextScoreRegister(J64) = NextScoreRegister(J64) * 1 'was * 1 / 2
Case Is = 5
EmergentNowScoreRegister(J64) = NowScoreRegister(J64) * 1 'was * 1  / 2
EmergentNextScoreRegister(J64) = NextScoreRegister(J64) * 1 'was * 1 / 2
Case Is = 6
EmergentNowScoreRegister(J64) = NowScoreRegister(J64) * 1 'was 2
EmergentNextScoreRegister(J64) = NextScoreRegister(J64) * 1 'was 2
Case Is = 7
EmergentNowScoreRegister(J64) = NowScoreRegister(J64) * 1 'was 2
EmergentNextScoreRegister(J64) = NextScoreRegister(J64) * 1 'was 2
Case Is = 8
EmergentNowScoreRegister(J64) = NowScoreRegister(J64) * 1 'was 2
EmergentNextScoreRegister(J64) = NextScoreRegister(J64) * 1 'was 2
Case Is = 9
EmergentNowScoreRegister(J64) = NowScoreRegister(J64) * 1 'was 2
EmergentNextScoreRegister(J64) = NextScoreRegister(J64) * 1 'was 2

Case Is > 9
EmergentNowScoreRegister(J64) = NowScoreRegister(J64) * 1 'was 3
EmergentNextScoreRegister(J64) = NextScoreRegister(J64) * 1 'was 3

'If CompatibilityTable(Activity(NewLineNumber, I64), J64) < 8 Then NowScoreRegister(J64) = 0 'try NSR =
NSR*table
'If CompatibilityTable(Activity(NewLineNumber, I64), J64) < 8 Then NextScoreRegister(J64) = 0

End Select
Next J64
Next I64

ZeroAddressError:

End Sub

Public Sub NowOrderSub() '7/02/2011
  Esp = "NowOrderSub"                    ' Maurice 24-05-2008 - Espionnage
   Espion

TempHighestNow(1) = 0
TempHighestNow(2) = 0

   For I8 = 1 To Enumber
   HighestNow(I8, 1) = I8
   HighestNow(I8, 2) = EmergentNowScoreRegister(I8)
   Next I8

      I8 = 0
      J8 = 0
For I8 = 1 To (Enumber - 1)
For J8 = (I8 + 1) To Enumber
```

```
If HighestNow(I8, 2) >= HighestNow(J8, 2) Then GoTo KeepHighestNow
TempHighestNow(1) = HighestNow(I8, 1)
TempHighestNow(2) = HighestNow(I8, 2)
HighestNow(I8, 1) = HighestNow(J8, 1)
HighestNow(I8, 2) = HighestNow(J8, 2)
HighestNow(J8, 1) = TempHighestNow(1)
HighestNow(J8, 2) = TempHighestNow(2)

KeepHighestNow:
Next J8

Next I8


End Sub

Public Sub NoiseSub()

'this forcibly inserts a randomly chosen element (that is not an input) into the Activity Register
Esp = "NoiseSub"
   Espion

R49 = Int((Enumber - (InputRange + 1)) * Rnd) + (InputRange + 1) 'to get elements between 1 and Enumber
If R49 > Enumber - OutputRange Then GoTo NoNoise


NowScoreRegister(R49) = 10 * NowNextRange * Anumber


NoNoise:
End Sub


Public Sub CycleSub()
   Esp = "CycleSub"                   ' Maurice 24-05-2008 - Espionnage
   Espion                             ' Maurice 24-05-2008 - Espionnage

Cycling = 0
'CycleLength says how many lines of the ActivityRegister there are per insertion of a CycleElement

CycleStep = CycleStep + 1
If CycleStep < CycleLength Then GoTo MissCycle

If CycleStep = CycleLength Then CycleStep = 0


CycleElement = CycleElement + 1
If CycleElement > Enumber Then CycleElement = 4
If CycleElement < 4 Then CycleElement = 4
If CycleElement > Enumber - OutputRange Then CycleElement = 4

Activity(NewLineNumber, 1) = CycleElement

'Let other routines know a CycleElement has been inserted
Cycling = 1
```

```
MissCycle:
End Sub


Public Sub ScrambleHighestNowSub()
   Esp = "ScrambleHighestNowSub"            ' Maurice 24-05-2008 - Espionnage
   Espion                              ' Maurice 24-05-2008 - Espionnage
'The problem to be solved (if it is a problem) is to prevent the same Nows from being loaded again and again even though
'there are other Nows with the same score. This is because the ordering routine puts the lowest addresses on top. So scramble them.
ScrambleNowNumber = 0
MarkScrambleNowStart = 0

For I49 = 1 To Anumber - 1
If MarkScrambleNowStart = 0 Then MarkScrambleNowStart = I49
If HighestNow(I49, 2) = HighestNow(I49 + 1, 2) Then ScrambleNowNumber = ScrambleNowNumber + 1

Select Case ScrambleNowNumber

Case Is = 0
'There is just one line so don't try to scramble!
MarkScrambleNowStart = 0
GoTo ContinueUpdatingNow

Case Is > 0

   Randomize
   R20 = Int((2 * Rnd)) '
      Select Case R20
      Case Is = 0
      Case Else
      TempScrambleNowAddress = HighestNow(MarkScrambleNowStart, 1)
      TempScrambleNowScore = HighestNow(MarkScrambleNowStart, 2)
      HighestNow(MarkScrambleNowStart, 1) = HighestNow(MarkScrambleNowStart + 1, 1)
      HighestNow(MarkScrambleNowStart, 2) = HighestNow(MarkScrambleNowStart + 1, 2)
      HighestNow(MarkScrambleNowStart + 1, 1) = TempScrambleNowAddress
      HighestNow(MarkScrambleNowStart + 1, 2) = TempScrambleNowScore
      End Select
ScrambleNowNumber = 0
MarkScrambleNowStart = 0

End Select

ContinueUpdatingNow:

Next I49

End Sub

Public Sub ScrambleHighestNextSub()
'THIS ROUTINE IS PROBABLY NONSENSICAL!!!!!
   Esp = "ScrambleHighestNextSub"          ' Maurice 24-05-2008 - Espionnage
   Espion                            ' Maurice 24-05-2008 - Espionnage
```

```
'The problem to be solved (if it is a problem) is to prevent the same Nexts from being loaded again and again even though
'there are other Nexts with the same score. This is because the ordering routine puts the lowest addresses on top. So scramble them.
ScrambleNextNumber = 0
MarkScrambleNextStart = 0
TempScrambleNextAddress = Enumber

'this goes through the ranked HighestNext array
For I52 = 1 To Anumber - 1
If MarkScrambleNextStart = 0 Then MarkScrambleNextStart = I52
If HighestNext(I52, 2) = HighestNext(I52 + 1, 2) Then ScrambleNextNumber = ScrambleNextNumber + 1

Select Case ScrambleNextNumber
Case Is = 0
'There is just one line so don't try to scramble!
MarkScrambleNextStart = 0
GoTo ContinueUpdatingNext
Case Is > 0
'If HighestNext(I52, 2) = HighestNext(I52 + 1, 2) Then GoTo ContinueUpdatingNext
'Scramble HighestNexts with the same score by swapping
   Randomize
   R21 = Int((2 * Rnd)) 'this gives 0 and 1
   Select Case R21
   Case Is = 0
   Case Else
   TempScrambleNextAddress = HighestNext(MarkScrambleNextStart, 1)
   TempScrambleNextScore = HighestNext(MarkScrambleNextStart, 2)
   HighestNext(MarkScrambleNextStart, 1) = HighestNext(MarkScrambleNextStart + 1, 1)
   HighestNext(MarkScrambleNextStart, 2) = HighestNext(MarkScrambleNextStart + 1, 2)
   HighestNext(MarkScrambleNextStart + 1, 1) = TempScrambleNextAddress
   HighestNext(MarkScrambleNextStart + 1, 2) = TempScrambleNextScore
   End Select
ScrambleNextNumber = 0
MarkScrambleNextStart = 0

End Select

ContinueUpdatingNext:

Next I52

End Sub

Public Sub InactivateElementsSub()
   Esp = "InactivateElementsSub"            ' Maurice 24-05-2008 - Espionnage
   Espion                            ' Maurice 24-05-2008 - Espionnage
'This prevents elements that have been active in the AR from being active again
'for a down time

'This allows neurones to recover progressively
   For I38 = 1 To Enumber
      Downtime(I38) = Downtime(I38) - 1
      If Downtime(I38) < 0 Then Downtime(I38) = 0
   Next I38
```

```
    For I39 = 1 To Anumber
        A = Activity(LineNumber, I39)
        If A = 0 Then GoTo AnumberIncreased
        Downtime(A) = DownTimeNumber '
AnumberIncreased:
    Next I39
    'This stops the IDP being degraded/inactivated:

    'Before the switch (which is when TimeToSwitch takes effect), IDP2=0
    Select Case TimeToSwitch
    Case Is = 0
    Downtime(IDP1) = 0
    Case Is = 1
    If IDP2 = 0 Then Downtime(IDP1) = 0 Else Downtime(IDP2) = 0
    Case Else
    End Select

'Must not prevent a response else cannot have IDP in successive rows when needed!
For I85 = 1 To OutputRange
Downtime(1 + Enumber - I85) = 0
Next I85

End Sub


Public Sub TestDecisionRewardOrPunishSub()
RewardDecision = 0
RunningScoreAction = 0
GoodNewOutput = 0

For I84 = 1 To OutputRange
For J84 = 1 To Anumber
If OutputA(I84) = 1 And Activity(NewLineNumber, J84) = (1 + Enumber) - I84 Then GoodNewOutput = 1
Next J84
Next I84


       If NumberOfOutputs > 1 Then GoodNewOutput = 0

    Select Case GoodNewOutput

    Case Is = 0 'CHOICE OF PUNISHMENTS
        'Example: StartLoop=17 where there is an output of 3998 (happens to be right); the following input is in
line 18
        'Set Endloop=18 so the punishing of the Nexts is in the loop from the Next fields of elements in line 17
        'to the elements in the following line BUT here this following line is (Endloop-1)= line 17 so just doing one
line!

        If Bigloop0 > LoopsTillStorm Then GoTo TooLateToPunish

        LineChoice = NewLineNumber - (OldOutputLine + 1)

        StartLoop = (OldOutputLine) + Int(LineChoice * Rnd)
        EndLoop = StartLoop
```

```
        For I81 = 1 To RepeatRewardPunish
        StartLoop = (OldOutputLine)
        EndLoop = StartLoop + 1
        PunishMutateNextSub
        Next I81

        'Startloop now =20 (17+1 + a random 2) Endloop=21
        StartLoop = (OldOutputLine + 1) + Int(LineChoice * Rnd)
        EndLoop = NewLineNumber
        For I81 = 1 To RepeatRewardPunish
        PunishMutateNowSub
        Next I81

TooLateToPunish:
        RecordFailure = RecordFailure + 1
        'CyclePermission = 1 'REMOVECYCLEPERMISSION

       Case Is = 1

       If Bigloop0 > LoopsTillStorm Then GoTo ToolatetoReward

    StartLoop = InputLine
    EndLoop = NewLineNumber

        For I81 = 1 To RepeatRewardPunish
       ShortRewardNowSub
           Next I81


    If GoodOldOutput = 1 Then StartLoop = OldOutputLine Else StartLoop = InputLine 'remove this addition to
Coco64?

        For I81 = 1 To RepeatRewardPunish
        ShortRewardNextSub
        Next I81

ToolatetoReward:

        RecordSuccess = RecordSuccess + 1

        'CyclePermission = 0 'REMOVECYCLEPERMISSION
End Select

 GoodOldOutput = GoodNewOutput
 OldOutputLine = NewLineNumber

'This is for MutationSub
RunningScorePointer = RunningScorePointer + 1
If RunningScorePointer > RunningScoreWindow Then RunningScorePointer = 1
    If GoodNewOutput = 1 Then RunningScore(RunningScorePointer) = 1
    If GoodNewOutput = 0 Then RunningScore(RunningScorePointer) = 0

End Sub
```

```
Public Sub RecordSuccessSub()

ShortTermMemoryLength = 20
FullSuccessStory = 0

StartLoop = SuccessTable(RecordPointer, 1)

I73 = RecordPointer
OutputResult = 1
Do Until OutputResult = 0
StartLoop = SuccessTable(I73, 1)
I73 = I73 - 1
If I73 = RecordPointer - ShortTermMemoryLength Then OutputResult = 0
If I73 = RecordPointer - ShortTermMemoryLength Then FullSuccessStory = 1

OutputResult = SuccessTable(I73, 3)
Loop

EndLoop = NewLineNumber

End Sub

Public Sub ShortRewardNowSub()

   If ActivateReward = 0 Then GoTo MissRandomRewardNow

I25 = 0
   J25 = 0

For ActRegLine = StartLoop To EndLoop

   For I25 = 1 To Anumber
   DontOverwriteNow = 0
AvoidDirectCouplingInputOutput:
AvoidSelfingRandomNow:
         Randomize
         R7 = Int((Anumber * Rnd) + 1)

         FirstElement = Activity(ActRegLine, I25)
         SecondElement = Activity(ActRegLine, R7)
         If FirstElement = 0 Then GoTo AnotherAnumberIncrease4
         If SecondElement = 0 Then GoTo AnotherAnumberIncrease4
         If FirstElement = SecondElement Then GoTo AvoidSelfingRandomNow

            Select Case TimeToSwitch
            Case Is = 0
            If FirstElement = IDP1 Then GoTo DoneIDPShortRewardNow
            Case Is = 1
            If FirstElement = IDP1 Then GoTo DoneIDPShortRewardNow
            If FirstElement = IDP2 Then GoTo DoneIDPShortRewardNow
            Case Else
            End Select

         TooBiased = 0
```

```
        I35 = 0
        For I35 = KNowNumber(FirstElement) To NowNextNumberMidpoint
        If Element(FirstElement, I35) = SecondElement Then TooBiased = TooBiased + 1
        Next I35
        If TooBiased > NowTooBiasedLimit Then GoTo AvoidCouplingInputToSecondHalfEnumberNow:



        NowGrowing = 0
        If KNowNumber(FirstElement) > KNowMax Then NowGrowing = 1

        Select Case NowGrowing

        Case Is = 0
         Randomize
         R6 = Int((NowNextNumberMidpoint - KNowNumber(FirstElement) * Rnd) + 1)
         'it needs this to learn properly:
          For J25 = 1 To Anumber 'avoid reward routine overwriting a good combination
          If Element(FirstElement, R6) = Activity(ActRegLine, J25) Then DontOverwriteNow = 1
          Next J25
        If DontOverwriteNow = 0 Then Element(FirstElement, R6) = SecondElement

        Case Is > 0
        KNowNumber(FirstElement) = KNowNumber(FirstElement) - 1
        Element(FirstElement, KNowNumber(FirstElement)) = SecondElement

        End Select

AvoidCouplingInputToSecondHalfEnumberNow:
AvoidCouplingEvenOddOrOddEven:
DoneIDPShortRewardNow:
      Next I25
AnotherAnumberIncrease4:
   Next ActRegLine

MissRandomRewardNow:

End Sub


Public Sub ShortRewardNextSub()

If ActivateReward = 0 Then GoTo MissRandomRewardNext

   I26 = 0
   J26 = 0
   I35 = 0

For ActRegLine = StartLoop To (EndLoop - 1)

   For I26 = 1 To Anumber
   DontOverwriteNext = 0
AvoidDirectCouplingInputNextOutput:
```

```
        Randomize
        R9 = Int((Anumber * Rnd) + 1)

        FirstElement = Activity(ActRegLine, I26)
        SecondElement = Activity(ActRegLine + 1, R9)
        If FirstElement = 0 Then GoTo AnotherAnumberIncrease3
        If SecondElement = 0 Then GoTo AnotherAnumberIncrease3
        If FirstElement = SecondElement Then GoTo AvoidCouplingNextElementToItself
        Select Case TimeToSwitch
         Case Is = 0
         If FirstElement = IDP1 Then GoTo DoneIDPShortRewardNext
         Case Is = 1
         If FirstElement = IDP1 Then GoTo DoneIDPShortRewardNext
         If FirstElement = IDP2 Then GoTo DoneIDPShortRewardNext
         Case Else
         End Select

       TooBiased = 0
        For I35 = (NowNextNumberMidpoint + 1) To KNextNumber(FirstElement)
          If Element(FirstElement, I35) = SecondElement Then TooBiased = TooBiased + 1
        Next I35
        If TooBiased > NextTooBiasedLimit Then GoTo MakeNoRandomNextReward

        NextGrowing = 0
        If KNextNumber(FirstElement) < KNextMax Then NextGrowing = 1

         Select Case NextGrowing

          Case Is = 0

          Randomize
          R8 = NowNextNumberMidpoint + Int(((KNextNumber(FirstElement) - NowNextNumberMidpoint) * Rnd)
+ 1)

           For J26 = 1 To Anumber 'avoid reward routine overwriting a good combination
           If Element(FirstElement, R8) = Activity(ActRegLine + 1, J26) Then DontOverwriteNext = 1

           Next J26
           If DontOverwriteNext = 0 Then Element(FirstElement, R8) = SecondElement

          Case Is > 0
          KNextNumber(FirstElement) = KNextNumber(FirstElement) + 1
          Element(FirstElement, KNextNumber(FirstElement)) = SecondElement

          End Select

AvoidCouplingInputToSecondHalfEnumberNext:
AvoidCouplingNextElementToItself:
AvoidCouplingEvenEvenOrOddOdd:
AvoidCouplingNext:
AvoidCouplingIDPtoNewElements:
DoneIDPShortRewardNext:

   Next I26
AnotherAnumberIncrease3:
```

```
MakeNoRandomNextReward:

   Next ActRegLine

MissRandomRewardNext:


End Sub


Public Sub DefineInputOutputSub()

'InputRange = 4 is defined in choices
'OutputRange = 2 is defined in choices
'set InputTotalMax to have the size of the pattern
'define inputA(inputrange) as integer
'OutputA(1)= 1 means we want enumber as output
'OutputA(2)= 1 means we want enumber-1 as output
'OutputA(3)= 1 means we want enumber-2 as output


For I82 = 1 To InputRange
InputA(I82) = 0
Next I82

For I83 = 1 To OutputRange
OutputA(I83) = 0
Next I83

'This gives the input pattern and the corresponding output pattern
Select Case InputTotal '1,2,3,4,5 ...1000,999,998,997,996

Case Is = 1
InputA(1) = 1
Downtime(1) = 0
OutputA(1) = 1

Case Is = 2
InputA(2) = 1
Downtime(2) = 0
OutputA(2) = 1

Case Is = 3
InputA(3) = 1
Downtime(3) = 0
OutputA(2) = 1

Case Is = 4
InputA(2) = 1
Downtime(2) = 0
OutputA(1) = 1

Case Is = 5
InputA(3) = 1
Downtime(3) = 0
```

```
OutputA(3) = 1

'Case Is = 6
'InputA(6) = 1
'OutputA(6) = 1

'Case Is = 7
'InputA(7) = 1
'OutputA(7) = 1

'Case Is = 8
'InputA(8) = 1
'OutputA(8) = 1

'Case Is = 9
'InputA(9) = 1
'OutputA(9) = 1
End Select


End Sub

Public Sub InputSub()
   Esp = "InputSub"              ' Maurice 24-05-2008 - Espionnage
   Espion                        ' Maurice 24-05-2008 - Espionnage

280                                          ' Repère 280
   ConstantInput = ConstantInput + 1
   If ConstantInput < KeepInputSameForThisNumber Then GoTo keepinput
   If ConstantInput = KeepInputSameForThisNumber Then ConstantInput = 1


'the input pattern is only changed when ConstantInput allows the following

   InputTotal = InputTotal + 1
   If InputTotal = InputTotalMax Then InputTotal = 1

DefineInputOutputSub


keepinput: 'load newline of Activity Register
285                                          ' Repère 285



'If Cycling = 1 there is already an address in the first 'position of the AR NewLine
   AfterInputPosition = 1
If Cycling = 1 Then AfterInputPosition = 2

InputAverageConnectivitySub

InputNeeded = 0

   InputLine = NewLineNumber
```

```
'END OF INPUT SECTION
End Sub


Public Sub InputAverageConnectivitySub()
Esp = "InputAverageConnectivitySub"
   Espion

'The original intention was to allow anticipation by using the Next scores of the previous line - it could still be done
'This gives a score to the input that should allow it to be inserted BUT make sure that the register is not set to zero after this!!!!!!!!!


   I32 = 0
   For I32 = 1 To InputRange
      Select Case Bigloop0
      Case Is < (LoopsTillStorm - 200)
      If InputA(I32) = 1 Then InputNowScore(I32) = 112 + (NowNextRange / Enumber) * Anumber 'or could be = HighestNow(200, 2)
      If InputA(I32) = 1 Then InputNextScore(I32) = 212 + (NowNextRange / Enumber) * Anumber 'or could be = HighestNext(20 * Anumber, 2)
      Case Else
      If InputA(I32) = 1 Then InputNowScore(I32) = 0
      If InputA(I32) = 1 Then InputNextScore(I32) = 0
      End Select
   Next I32
End Sub

Public Sub LongTermMemorySub()
   Esp = "LongTermMemorySub"            ' Maurice 24-05-2008 - Espionnage
   Espion                               ' Maurice 24-05-2008 - Espionnage
'The LTM contains the essence of the Elements table and is a matrix Elements x Elements.
'It is updated depending on RunningScore (i.e. the equivalent of laying down a short term memory) if thngs go well
'It is linked to Downtime so that an important connection is not be disrupted readily
'It can be used to reconstruct the Elements table after a period in which there has been no real progress


For I54 = 1 To Enumber
For J54 = 1 To Enumber
LTMemory(I54, J54) = 0
Next J54
Next I54


For I54 = 1 To Enumber
For K54 = 1 To NowNextRange
J54 = Element(I54, K54)
If J54 = 0 Then GoTo MissZeroElement
LTMemory(I54, J54) = LTMemory(I54, J54) + SignElement(I54, K54)
MissZeroElement:
Next K54
Next I54
```

```
End Sub

Public Sub UpdateLineNumberSub()
   Esp = "UpdateLineNumberSub"              ' Maurice 24-05-2008 - Espionnage
   Espion                              ' Maurice 24-05-2008 - Espionnage
290                                                    ' Repère 290
   LineNumber = LineNumber + 1
   If LineNumber = EndOfActivityRegister + 1 Then LineNumber = 1
   NewLineNumber = LineNumber + 1
   If LineNumber = EndOfActivityRegister Then NewLineNumber = 1
   If LineNumber = 1 Then NewLineNumber = 2
End Sub

Public Sub IDPReplacementSub()
'LearntElement(DowntimeNumber * Anumber) This is where all the elements in the old Active subset have been recorded
'IDP2Element(DowntimeNumber * Anumber, 2) Allows conversion of the old Active subset into the new Active subset

Dim ReplaceByThis As Integer

Maurice_AffTbÉlémFinProg

'First load the first column of IDP2Element(x,1), which is full of zeroes to start wtih
For I99 = 1 To (2 * DownTimeNumber * Anumber) 'Goes through the elements in the 24 stored lines of the Activity Register
For K99 = (I99 - 1) To 1 Step -1
If IDP2Element(1, 1) = 0 Then GoTo ZeroNotAProblem
If LearntElement(I99) = IDP2Element(K99, 1) Then GoTo AlreadyLoaded 'this means that an element address will only be loaded once
ZeroNotAProblem:
Next K99
IDP2Element(I99, 1) = LearntElement(I99)
AlreadyLoaded: 'There will be a zero in the IDP2Element(I99,1) where there would otherwise have been a duplicate address
Next I99

'This gives the second column of IDP2Element matrix with the learnt element and the new element to which it should be converted
For I99 = 1 To (2 * DownTimeNumber * Anumber) 'Goes through the elements in the lines of the Activity Register
If IDP2Element(I99, 1) = 0 Then GoTo IgnoreZero

   Select Case IDP2Element(I99, 1)

   Case Is < InputRange
   IDP2Element(I99, 2) = IDP2Element(I99, 1) + 3
   KNextNumber(IDP2Element(I99, 2)) = KNextNumber(IDP2Element(I99, 1))
   KNowNumber(IDP2Element(I99, 2)) = KNowNumber(IDP2Element(I99, 1))
   Downtime(IDP2Element(I99, 2)) = Downtime(IDP2Element(I99, 1))

   Case Is <= Enumber - OutputRange
RepeatReplacement:

   R72 = InputRange + Int(((Enumber - OutputRange - InputRange) * Rnd))
```

```
      For J99 = 1 To (2 * DownTimeNumber * Anumber) 'There is a place vacant in the conversion matrix
      If R72 = LearntElement(J99) Then GoTo RepeatReplacement 'is the candidate new element in the old Active subset?
      If R72 = IDP2Element(J99, 2) Then GoTo RepeatReplacement 'has the candidate new element been used already as a new element?
      Next J99
   IDP2Element(I99, 2) = R72
   KNextNumber(R72) = KNextNumber(IDP2Element(I99, 1))
   KNowNumber(R72) = KNowNumber(IDP2Element(I99, 1))
   Downtime(IDP2Element(I99, 2)) = 0 'Downtime(IDP2Element(I99, 1))

   Case Else 'This is an output THERE IS A NEW BIT WITH THE -3
   IDP2Element(I99, 2) = IDP2Element(I99, 1) - 3
   KNextNumber(IDP2Element(I99, 2)) = KNextNumber(IDP2Element(I99, 1))
   KNowNumber(IDP2Element(I99, 2)) = KNowNumber(IDP2Element(I99, 1))
   Downtime(IDP2Element(I99, 2)) = 0 'Downtime(IDP2Element(I99, 1))

   End Select

IgnoreZero:
   Next I99

'Find IDP2
   For I99 = 1 To (2 * DownTimeNumber * Anumber)
   If IDP2Element(I99, 1) = IDP1 Then IDP2 = IDP2Element(I99, 2)
   Next I99

'Make a duplicate Element(Enumber, NowNextRange) matrix
'BUT it is too short for IDPs
For I99 = 1 To Enumber
   For J99 = 1 To IDPNowNextRange
   ReplacementElement(I99, J99) = Element(I99, J99)
   Next J99
Next I99

   For I99 = 1 To (2 * DownTimeNumber * Anumber)
   If IDP2Element(I99, 1) = IDP1 Then IDP2 = IDP2Element(I99, 2)
   Next I99
   'The IDP2 fields are bigger than those of other elements done above
   KNowNumber(IDP2) = 1
   KNextNumber(IDP2) = IDPNowNextRange

'convert the Now and Next fields of all the IDP2Element(x,1) and
'paste them into the fields of the IDP2Element(x,2)
'This is explained with a PowerPoint entitled IDPReplacementSub
''This allows ReplacementElement(X, position in field) to then replace Element(X, position in field)
'The numbers refer to the PowerPoint
For I99 = 1 To (2 * DownTimeNumber * Anumber)
If IDP2Element(I99, 1) = 0 Then GoTo IgnoreZero2
'now go through the two column IDP2element() matrix:
   Select Case IDP2Element(I99, 1)

   Case Is < InputRange '
   'goes through the fields of the successful element that will be replaced seeing if they contain an element to be replaced
```

```
    J99 = 0
    For J99 = 1 To NowNextRange
    ReplaceThis = ReplacementElement(IDP2Element(I99, 1), J99)
    If ReplaceThis = 0 Then GoTo IgnoreZero3
        For K99 = 1 To (2 * DownTimeNumber * Anumber)
        Select Case ReplaceThis
        Case Is = IDP2Element(K99, 1) 'IDP2Element(9, 1)=25
        ReplaceByThis = IDP2Element(K99, 2) 'IDP2Element(9, 2) = 44
        ReplacementElement(IDP2Element(I99, 2), J99) = IDP2Element(K99, 2)
        Sign = SignElement(IDP2Element(I99, 1), ReplaceThis)
        SignElement(IDP2Element(I99, 2), ReplaceByThis) = Sign
        Downtime(ReplaceByThis) = 0 'Downtime(ReplaceThis)
        Case Else
        End Select
        Next K99
IgnoreZero3:
    Next J99

    Case Is = IDP1 'i.e., IDP2Element(I99, 1) = IDP1
    J99 = 0
    For J99 = 1 To (IDPNowNextRange - 1) Step 2 'goes through the fields of the successful element that is to be replaced to see if they contain an element to be replaced
    ReplaceThis = Element(IDP1, J99) 'remember, this = Element(10, position in fields)
    If ReplaceThis = 0 Then GoTo IgnoreZero8
        For K99 = 1 To (2 * DownTimeNumber * Anumber)
        Select Case ReplaceThis 'remember, this = Element(10, field position)
        Case Is = IDP2Element(K99, 1) 'found the address in the IDP2Element conversion matrix
        ReplaceByThis = IDP2Element(K99, 2)
        ReplacementElement(IDP2, J99) = ReplaceByThis
        ReplacementElement(IDP2, J99 + 1) = ReplaceByThis
        Sign = SignElement(IDP2Element(I99, 1), ReplaceThis)
        SignElement(IDP2Element(I99, 2), ReplaceByThis) = Sign
        Downtime(ReplaceByThis) = 0 'Downtime(ReplaceThis)
        Case Else
        End Select
        Next K99
IgnoreZero8:
    Next J99

    Case Else 'Is <= Enumber - OutputRange
    J99 = 0
    For J99 = 1 To NowNextRange 'goes through the fields of the successful element that is to be replaced to see if they contain an element to be replaced
    ReplaceThis = ReplacementElement(IDP2Element(I99, 1), J99)
    'IDP2Element(I99, 1) = 27 so ReplacementElement(27, 1) =25
    If ReplaceThis = 0 Then GoTo IgnoreZero4
        For K99 = 1 To (2 * DownTimeNumber * Anumber)
        Select Case ReplaceThis
        Case Is = IDP2Element(K99, 1) 'IDP2Element(9, 1)=25
        ReplaceByThis = IDP2Element(K99, 2) 'IDP2Element(9, 2) = 44
        ReplacementElement(IDP2Element(I99, 2), J99) = IDP2Element(K99, 2)
        Sign = SignElement(IDP2Element(I99, 1), ReplaceThis)
        SignElement(IDP2Element(I99, 2), ReplaceByThis) = Sign
        Downtime(ReplaceByThis) = 0 ' Downtime(ReplaceThis)
        Case Else
```

```
      End Select
      Next K99
IgnoreZero4:
   Next J99

   End Select
IgnoreZero2:
Next I99

'Rewrite Elements
'Replace Element(Enumber, NowNextRange) matrix
For I99 = 1 To Enumber
For J99 = 1 To IDPNowNextRange
Element(I99, J99) = ReplacementElement(I99, J99)
Next J99
Next I99

For J99 = 1 To IDPNowNextRange
Element(IDP2, J99) = ReplacementElement(IDP2, J99)
Next J99

Maurice_AffTbÉlémFinProg

IDP2NowReplaced = 1
End Sub

Public Sub IDPExtractLearntElementsSub()

I98 = LineNumber '
   For J98 = 1 To Anumber
   LearntElement(LearntCounter) = Activity(I98, J98)
   If LearntElement(LearntCounter) < 1 Then LearntElement(LearntCounter) = 1
   LearntCounter = LearntCounter + 1
   Next J98

End Sub


Public Sub ForcedElementsInsertionSub()

'This finds elements to be inserted AT RANDOM that are NOT in the Active subset (i.e., have a StormDownTime>0)
'because all elements that have recently been in the Active subset have a StormDownTime=1
Maurice_AffTbÉlémFinProg
InsertionScore = KNowMin
For I100 = 1 To Anumber
For J100 = InputRange To (Enumber - OutputRange)
If StormDownTime(J100) = 1 Then GoTo NewStormElementNeeded
If KNowNumber(J100) > InsertionScore Then GoTo NewStormElementNeeded
 If UseIDP = 1 And J100 = IDP1 Then GoTo NewStormElementNeeded
StormElement = J100
InsertionScore = KNowNumber(StormElement)
NewStormElementNeeded:
 Next J100
 StormDownTime(StormElement) = 1
```

```
 Downtime(StormElement) = DowntimeMax(StormElement)
 Next I100

End Sub



Public Sub PunishMutateNowSub()
   'Esp = "PunishMutateNowSub"                 ' Maurice 24-05-2008 - Espionnage
   'Espion                               ' Maurice 24-05-2008 - Espionnage

If ActivatePunish = 0 Then GoTo MissOnPunishNows

For ActRegLine = StartLoop To EndLoop

If ActRegLine = 0 Then GoTo MissActRegLineZero
PositionAR1 = 0
PositionNow1 = 0

For PositionAR1 = 1 To Anumber

         FirstElement = Activity(ActRegLine, PositionAR1)
         If FirstElement = 0 Then GoTo AnotherAnumberIncrease2

         Select Case TimeToSwitch
         Case Is = 0
         If FirstElement = IDP1 Then GoTo DoneIDPPunishMutateNow
         Case Is = 1
         If FirstElement = IDP1 Then GoTo DoneIDPPunishMutateNow
         If FirstElement = IDP2 Then GoTo DoneIDPPunishMutateNow
         Case Else
         End Select

     If KNowNumber(FirstElement) < KNowMin Then Element(FirstElement, KNowNumber(FirstElement)) = 0

     If KNowNumber(FirstElement) < KNowMin Then KNowNumber(FirstElement) = KNowNumber(FirstElement)
+ 1 'shorten KNow field

      For PositionNow1 = (KNowNumber(FirstElement)) To NowNextNumberMidpoint
Randomize
MutationNow = Int((100 * Rnd) + 1)
If MutationNow < MutationThreshold Then GoTo NoPunishMutateNow
Sevencycle:
            Randomize
            R2 = Int((Enumber * Rnd) + 1)


'Avoid creating inputs at random
If R2 <= InputRange Then GoTo Sevencycle

If R2 = FirstElement Then GoTo Sevencycle

Element(FirstElement, PositionNow1) = R2
NoPunishMutateNow:
         Next PositionNow1
```

```
DoneIDPPunishMutateNow:
   Next PositionAR1
AnotherAnumberIncrease2:
MissActRegLineZero:
 Next ActRegLine

MissOnPunishNows:

End Sub

Public Sub PunishMutateNextSub()
   'Esp = "PunishMutateNextSub"               ' Maurice 24-05-2008 - Espionnage
   'Espion                              ' Maurice 24-05-2008 - Espionnage

If ActivatePunish = 0 Then GoTo MissOnPunishNexts

If StartLoop = 0 Then StartLoop = 1
For ActRegLine = StartLoop To (EndLoop - 1)
PositionAR2 = 0
PositionNext1 = 0
For PositionAR2 = 1 To Anumber

       FirstElement = Activity(ActRegLine, PositionAR2)
       If FirstElement = 0 Then GoTo AnotherAnumberIncrease1
       Select Case TimeToSwitch
       Case Is = 0
       If FirstElement = IDP1 Then GoTo DoneIDPPunishMutateNext
       Case Is = 1
       If FirstElement = IDP1 Then GoTo DoneIDPPunishMutateNext
       If FirstElement = IDP2 Then GoTo DoneIDPPunishMutateNext
       Case Else
       End Select

       If KNextNumber(FirstElement) > KNextMin Then Element(FirstElement, KNextNumber(FirstElement)) = 0
       If KNextNumber(FirstElement) > KNextMin Then KNextNumber(FirstElement) =
KNextNumber(FirstElement) - 1

              For PositionNext1 = (NowNextNumberMidpoint + 1) To KNextNumber(FirstElement)


                 Select Case TimeToSwitch 'only for Coco826
                 Case Is = 0
                 If Element(IDP1, PositionNext1) = IDP1 Then GoTo NoPunishMutateNext
                 Case Is = 1
                 If Element(IDP1, PositionNext1) = IDP1 Then GoTo NoPunishMutateNext
                 Case Else
                 End Select

          MutationNext = Int((100 * Rnd) + 1)
          If MutationNext < MutationThreshold Then GoTo NoPunishMutateNext

Eightcycle:
          Randomize
          R4 = Int((Enumber * Rnd) + 1)
```

```
            'no selfing
            If R4 = FirstElement Then GoTo Eightcycle
            'no spurious input
            If R4 <= InputRange Then GoTo Eightcycle

'Avoid creating outputs at random


Element(FirstElement, PositionNext1) = R4

NoPunishMutateNext:
    Next PositionNext1
DoneIDPPunishMutateNext:
  Next PositionAR2
AnotherAnumberIncrease1:

 Next ActRegLine

MissOnPunishNexts:

End Sub



Public Sub RemoveSelfingSub() 'IMPORTANT - THIS SUBROUTINE IS NOT USED AND Knumber needs changing
  Esp = "RemoveSelfingSub"                  ' Maurice 24-05-2008 - Espionnage
  Espion                            ' Maurice 24-05-2008 - Espionnage

'eliminates self-referencing
320                                                    ' Repère 320
  I19 = 0
  J19 = 0
  For I19 = 1 To Enumber
    For J19 = 1 To NowNextRange
ReplaceCycle:
      Randomize
      R1 = Int((Enumber * Rnd) + 1)
      If I19 = Element(I19, J19) Then Element(I19, J19) = R1
      If I19 = R1 Then GoTo ReplaceCycle
    Next J19
  Next I19

End Sub

Public Sub RemoveInputGenerationByCocoSub()
  Esp = "RemoveInputGenerationByCocoSub"      ' Maurice 24-05-2008 - Espionnage
  Espion                            ' Maurice 24-05-2008 - Espionnage
'Eliminates a second input coming from Coco
'If there is a real input, it is in the first position in the AR
'so check the New line and the following lines for a second input and replace it at random
'this sub MUST be disabled if we want to run in an anticipatory mode in which inputs are predicted

I45 = 0
J45 = 0
InputTally = 0
```

```
RandomizeAgain = 0

Select Case NewLineNumber

Case Is = InputLine

   For I45 = 1 To Anumber
If Activity(NewLineNumber, I45) > 3 Then GoTo DontDoAnything1

   InputTally = InputTally + 1
   If InputTally < 2 Then GoTo DontDoAnything1
ReplaceInput1:
   Randomize
   RandomizeAgain = 0
   R16 = Int((Enumber * Rnd) + 1)
   If R16 < 4 Then RandomizeAgain = 1
   If R16 > Enumber - OutputRange Then RandomizeAgain = 1
   If RandomizeAgain = 1 Then GoTo ReplaceInput1
      For J45 = 1 To Anumber
      If R16 = Activity(NewLineNumber, J45) Then RandomizeAgain = 1
      Next J45
   If RandomizeAgain = 1 Then GoTo ReplaceInput1
   Activity(NewLineNumber, I45) = R16
   InputTally = InputTally - 1

DontDoAnything1:
   Next I45

Case Else

   For I45 = 1 To Anumber
If Activity(NewLineNumber, I45) > 3 Then GoTo DontDoAnything2
If Activity(NewLineNumber, I45) = Activity(InputLine, 1) Then GoTo DontDoAnything2 'allows the same input to
be repeated
   InputTally = InputTally + 1
   If InputTally < 1 Then GoTo DontDoAnything2
ReplaceInput2:
   Randomize
   RandomizeAgain = 0
   R16 = Int((Enumber * Rnd) + 1)
   If R16 < 4 Then RandomizeAgain = 1
   If R16 > Enumber - OutputRange Then RandomizeAgain = 1
   If RandomizeAgain = 1 Then GoTo ReplaceInput2
      For J45 = 1 To Anumber
      If R16 = Activity(NewLineNumber, J45) Then RandomizeAgain = 1
      Next J45
   If RandomizeAgain = 1 Then GoTo ReplaceInput2
   Activity(NewLineNumber, I45) = R16
   InputTally = InputTally - 1
DontDoAnything2:
   Next I45

   End Select
```

```
End Sub
Public Sub RemoveSpuriousInputSub()
   Esp = "RemoveSpuriousInputSub"             ' Maurice 24-05-2008 - Espionnage
   Espion                              ' Maurice 24-05-2008 - Espionnage
'Eliminates inputs in all fields so the system cannot learn!
330                                                       ' Repère 330
   I20 = 0
   J20 = 0
   For I20 = 1 To Enumber
      For J20 = KNowMin To KNextMin
         If Element(I20, J20) > 3 Then GoTo ThisIsNotAnInput
ReplaceBiCycle:
         Randomize
         R3 = Int((Enumber * Rnd) + 1)
         If R3 < 4 Then GoTo ReplaceBiCycle
         Element(I20, J20) = R3
ThisIsNotAnInput:
      Next J20
   Next I20
'
End Sub


'
' %%%%%%%%%%%%%%%%%%%%%%%%%%%%%%%% MAURICE 05-06-2008 - PAQUET DES SÉQUENCES
MAURICE %%%%%%%%%%%%%%%%%%%%%%%%%%%%%%%%%%%%%%%%%%%%
'Sub Patch()        ' REMPLACER TOUT LE PAQUET MAURICE PAR CELUI CI-DESSOUS ET SUPPRIMER CETTE LIGNE
Sub
'
' %%%%%%%%%%%%%%%%%%%%%%%%%%%%%%%% MAURICE 05-06-2008 - PAQUET DES SÉQUENCES
MAURICE %%%%%%%%%%%%%%%%%%%%%%%%%%%%%%%%%%%%%%%%%%%%

' %%%%%%%%%%%%%%%%%%%%%%%%  Maurice - 05-06-2008 - Événement bouton de commande Spy
%%%%%%%%%%%%%%%%%%%%%%%%%%%%%%%%%%%%%%%%%%%%%%%%%%
'                                                                   '%
Private Sub Espionnage_Click()                                      '%
   Spy = 1 - Spy                                                    '%
   If Spy = 1 Then                                                  '%
      Annexe.Visible = True                                         '%
   Else                                                             '%
      Annexe.Visible = False                                        '%
   End If                                                           '%
End Sub                                                             '%
'
%%%%%%%%%%%%%%%%%%%%%%%%%%%%%%%%%%%%%%%%%%%%%%%%%%%%%%%%%%%%%%%%%%%%%
%%%%%%%%%%%%%%%%%%%%%%%%%%%%%%%%%%%%%%%%%%%%%%%%%%%%%%%%%%%%%%%%%%%%%
%%

'
'%%%%%%%%%%%%%%%%%%%%%%%%%%%  Maurice - 05-06-2008 - Fin des affichages en fin de
programme  %%%%%%%%%%%%%%%%%%%%%%%%%%%%%%%%%%%%%%%%
'

 Private Sub Espion()
'
```

```
' %%%%%%%%%%%%%%%%%%%%%%  Maurice - 05-06-2008 - Enregistrement Espions
%%%%%%%%%%%%%%%%%%%%%%%%%%%%%%%%%%%%%%%%%%%%%%%%%%%%%%%%
Espion:                                                        '%
  If Esp1 <> 1 Then                                            '%
    xe = 200: ye = -300        ' Passage 1ère fois             '%
    Esp1 = 1                                                   '%
    GoTo 2                                                     '%
  End If                                                       '%
2                                'Repère 2                     '%
                                                               '%
  If ye > 13500 Then          ' Bas de page, on efface et on reprend en haut de page    '%
    Annexe.Cls                                                 '%
    ye = 400                                                   '%
  End If                                                       '%
'   xe = xe + 4000            ' Mettre en service si une donnée accompagne le nom de Sub   '%
  ye = ye + 300                                                '%
  Annexe.CurrentX = xe: Annexe.CurrentY = ye                   '%
  Annexe.Print Esp                                             '%
Attente:                                                       '%
  DoEvents                                                     '%
  If Spy = 1 Then GoTo Attente                                 '%
'                                                              '%
 End Sub                                                       '%
'%%%%%%%%%%%%%%%%%%%%%%%%%%%  Maurice - 05-06-2008 - Fin enregistrement Espion
%%%%%%%%%%%%%%%%%%%%%%%%%%%%%%%%%%%%%%%%%%%%%%%%%%

'

'
' %%%%%%%%%%%%%%%%%%%%%%%%%%%%%%%% MAURICE 05-06-2008 - PAQUET DES SÉQUENCES
MAURICE %%%%%%%%%%%%%%%%%%%%%%%%%%%%%%%%%%%%%%%%%%%%%%%
'Sub Patch()       ' REMPLACER TOUT LE PAQUET MAURICE PAR CELUI CI-DESSOUS ET SUPPRIMER CETTE LIGNE
Sub
'
' %%%%%%%%%%%%%%%%%%%%%%%%%%%%%%%% MAURICE 05-06-2008 - PAQUET DES SÉQUENCES
MAURICE %%%%%%%%%%%%%%%%%%%%%%%%%%%%%%%%%%%%%%%%%%%%%%%
'
'
'
'%%%%%%%%%%%%%%%%%%%%%%%%%%%  Maurice 05-06-2008 - Début affichage tableau Elément sur
Pause  %%%%%%%%%%%%%%%%%%%%%%%%%%%%%%%%%%%%%%%%
Public Sub Maurice_AffTbÉlémPause()                            '%
'%
  Esp = "Maurice_AffTbÉlémPause"       ' Maurice 03-06-2008 - Espionnage      '%
  Espion                      ' Maurice 03-06-2008 - Espionnage               '%
600                         ' Repère 600 sur lequel on revient si l'option Pause n'est pas active     '%
  DoEvents                                                     '%
  Dim PremierStop As String           ' Maurice 04-05-2008 - Prise en compte de la commande Stop
'%
  If Stopper = 0 Then                                          '%
    If PremierStop = "Oui" Then End                            '%
  End If                                                       '%
  If Stopper = 1 Then                                          '%
    PremierStop = "Oui"                                        '%
    GoTo 600                                                   '%
```

```
   End If                                                                          '%
'
%%%%%%%%%%%%%%%%%%%%%%%%%%%%%%%%%%%%%%%%%%%%%%%%%%%%%%%%%%%%%%%
%%%%%%%%%%%%%%%%%%%%%%%%%%%%%%%%%%%%%%%%%%%%%%%%%%%%%%%%%%%%%%%
%%
601
'  %%%%%%%%%%%%%%%%%%%%%%%%%%%%%%  Maurice 05-06-2008 - Affichages commande Pause
%%%%%%%%%%%%%%%%%%%%%%%%%%%%%%%%%%%%%%%%%%%%%%%%%%%%%%%%
   Dim Fin_affiche As String                '08-06-2008                                        '%
   Dim xt, yt, i, j, MaxLgn, MaxCol As Integer              ' 08-06-2008                       '%
'%%%%%%%%%%%%%%%%%%%%%%%%%                                                                      '%
'Susp = 1 - Susp '<---¤¤¤¤ POUR SIMULATION COMMANDE PAUSE - LAISSER CETTE INSTRUCTION EN
COMMENTAIRE ¤¤¤¤                  '%
'%%%%%%%%%%%%%%%%%%%%%%%%%                                                                      '%
   If Susp = 0 Then                                                                     '%
      Fin_affiche = "Non"                        ' Bouton "Pause" est Off, on saute l'affichage          '%
      GoTo 650                                                                         '%
   End If                                                                              '%
   If Fin_affiche = "Oui" Then GoTo 600                  ' Attente du bouton "Pause" en position On                 '%
'            %%%%  Test de l'option choisie %%%%           ' %%%% Maurice 03-06-2008 %%%%
'%
'                                                                                      '%
   If Enumber > 32 Then MaxLgn = 32 Else MaxLgn = Enumber      ' Pour éviter dépassement de capacité - 08-06-
2008            '%
   If NowNextRange > 10 Then MaxCol = 10 Else MaxCol = NowNextRange      ' Pour éviter dépassement de
capacité - 08-06-2008            '%
'                                                                                      '%
   If Option1.Value = True Then GoTo Affiche_Éléments        '                                  '%
   If Option2.Value = True Then GoTo Affiche_LongTermMemorySub    '                             '%
'                                                                                      '%
Affiche_Éléments:                                                                        '%
 Cls                                                                                   '%
 Form1.Font.Size = 8                                                                     '%
 CurrentX = 3100: CurrentY = 6410                                                           '%
 Print " Now                                                   "                  '%
 CurrentX = 3100: CurrentY = 6410                                                           '%
 ForeColor = RGB(255, 0, 0)                                                                 '%
 Print "                                                    Next"             '%
 ForeColor = RGB(0, 0, 0)                                                                  '%
 xt = 1000: yt = 6660                                                                     '%
'                                                                                      '%
602                                                                                    '%
 For i = 1 To MaxLgn                            ' Pour éviter dépassement de capacité - 08-06-2008           '%
'%
    CurrentX = 900: CurrentY = yt                                                          '%
    ForeColor = RGB(0, 0, 255)                                                             '%
    Print i                                                                            '%
    ForeColor = RGB(0, 0, 0)                                                               '%
    For j = 1 To 2 * MaxCol                        ' Pour éviter dépassement de capacité - 08-06-2008           '%
       If j = 11 Then xt = xt + 800 Else xt = xt + 400                                          '%
       If j > 10 Then ForeColor = RGB(255, 0, 0)                                               '%
       CurrentX = xt: CurrentY = yt                                                        '%
       Print Element(i, j)                                                              '%
    Next j                                                                             '%
   ForeColor = RGB(0, 0, 0)                                                                '%
```

```
   xt = 1000: yt = yt + 245                                           '%
 Next i                                                          '%
   GoTo Fin_Affichage                    ' %%%% Maurice 03-06-2008 %%%%                    '%
'                                    '                           '%
Affiche_LongTermMemorySub:                      '                              '%
'                                   ' %%%% Maurice 04-06-2008 pour toute la séquence %%%%        '%
'%
   Dim Tltm, Hltm, Vltm, Xltm, Yltm As Integer                                   '%
   Cls                                                           '%
   CurrentX = 150: Xltm = 150                                           '%
   CurrentY = 6450: Yltm = 6450                                          '%
   ForeColor = RGB(0, 0, 255)                                          '%
   For Tltm = 1 To MaxLgn                 ' Pour éviter dépassement de capacité - 08-06-2008          '%
      Xltm = Xltm + 580: CurrentX = Xltm: CurrentY = Yltm                              '%
      Print Tltm                                                    '%
   Next Tltm                                                        '%
'                                                                '%
   For Hltm = 1 To MaxLgn                 ' Pour éviter dépassement de capacité - 08-06-2008          '%
      Yltm = Yltm + 240                                               '%
      CurrentX = 150: Xltm = 150                                          '%
      CurrentY = Yltm                                                 '%
      ForeColor = RGB(0, 0, 255)                                        '%
      Print Hltm                                                    '%
      ForeColor = RGB(0, 0, 0)                                         '%
      For Vltm = 1 To MaxLgn              ' Pour éviter dépassement de capacité - 08-06-2008          '%
         Xltm = Xltm + 580: CurrentX = Xltm                                  '%
         CurrentY = Yltm                                              '%
         Print LTMemory(Hltm, Vltm)                                       '%
      Next Vltm                                                     '%
    Next Hltm                                                       '%
'                                                                '%
Fin_Affichage:                          '                           '%
   Fin_affiche = "Oui"                     '                          '%
'                                                                '%
' %%%%%%%%%%%%%%%%%%%%%%%%%%%%%%%%%%%%%%%% Maurice 05-06-2008 - Écrire du tableau sur classeur
Excel si nb de captures > 0 %%%%%%%%%%%%%%%%
'                     Ce classeur est nommé Coco_Capture.xls et se trouve directement sur C:              '%
%
620                                  ' Repère 620                            '%
'                                                                '%
                                                           '%
'                                                                '%
Séq_Excel:                                                          '%
 Dim TbExcel(30, 20)                        ' Table miroir du tableau des Elément pour écriture sur Excel       '%
 Dim LgTitreNow, ClTitreNow, LgTitreNext, ClTitreNext, Valij, incr, Couleur As Integer                    '%
 Dim ValTitreNow, ValTitreNext, Path As String                                   '%
 LgTitreNow = 3: ClTitreNow = 7: ValTitreNow = "Now"       ' Titrage des tableaux capturés
'%
 LgTitreNext = 3: ClTitreNext = 19: ValTitreNext = "Next"   '                              '%
 Path = "C:\Coco_Capture.xls"                 '                                    '%
 If Not Dir(Path) = "" Then Kill Path              ' Suppression du précédent classeur Coco_Capture.xls s'il existe
'%
'                                                                '%
'    'Capture_ctr.Text = Capture_ctr.Text + 1            ' + une capture effectuée                       '%
   Ctr = Ctr + 1                          ' Compteur numérique, meilleur pour servir d'indice - Repère Paquet '%
```

```
   For i = 1 To MaxLgn                          ' Installation dans les cellules du tableau capturé - 08-06-2008    '%
      For j = 1 To 2 * MaxCol                          '        - id -                        - 08-06-2008    '%
         If j > 10 Then                          '            - id -                              '%
            Couleur = 3                          '            - id -                              '%
            incr = 1                          '            - id -                              '%
         Else                          '            - id -                              '%
            Couleur = 1                          '            - id -                              '%
            incr = 0                          '            - id -                              '%
         End If                          '            - id -                              '%
         'xlSheet.Cells(i + 5, j + incr).Value = Element(i, j)  '        - id -                              '%
%
      Next j                          '            - id -                              '%
   Next i                          '            - id -                              '%
'                                                              '%
   GoTo 600                                                              '%
Sauvegarde:                          '                                   '%
                          '%
   Save_faite = 1                                                              '%
                    '      - id -                              '%
'                                                              '%
%
   GoTo 600                                                              '%
650                          ' Repère 650                              '%
'                                                              '%
' %%%%%%%%%%%%%%%%%%%%%%%%%%%%%%%%%%%%% Maurice - 05-06-2008 - Fin écriture du
tableau sur classeur Excel  %%%%%%%%%%%%%%%%%%%%%
'
'  %%%%%%%%%%%%%%%%%%%%%%%%%%%%%  Maurice 05-06-2008 - Fin affichage sur commande Pause
%%%%%%%%%%%%%%%%%%%%%%%%%%%%%%%%%%%%%%%%%%
'                                                              '%
End Sub                                                              '%

'
'%%%%%%%%%%%%%%%%%%%%%%%%%%%  Maurice - 05-06-2008 - Début des affichages en fin de
programme  %%%%%%%%%%%%%%%%%%%%%%%%%%%%%%%%%%%
'                                                              '%
Public Sub Maurice_AffTbÉlémFinProg()       '                              '%
'%
   Esp = "Maurice_AffTbÉlémFinProg"        ' Maurice 03-06-2008 - Espionnage                              '%
   Espion                          ' Maurice 03-06-2008 - Espionnage                              '%
   If Option1.Value = True Then GoTo Affiche_Élémentsf        '          - id -                              '%
   'If Option2.Value = True Then GoTo Affiche_LongTermMemorySubf   '          - id -                              '%
'                                                              '%
'          1) - Tableau des éléments                                                              '%
Affiche_Élémentsf:                                                              '%
   Dim xt, yt, i, j As Integer                                                              '%
   Cls                                                              '%
   CurrentX = 3100: CurrentY = 6410                                                              '%
   'Print " Now                                                  "                '%
   CurrentX = 3100: CurrentY = 6410                                                              '%
   ForeColor = RGB(255, 0, 0)                                                              '%
   'Print "                                                  Next"            '%
   ForeColor = RGB(0, 0, 0)                                                              '%
   xt = 1000: yt = 6660                                                              '%
   For i = 1 To Enumber                          ' Attention si Enumber > 32, dépassement capacité          '%
```

```
        CurrentX = 900: CurrentY = yt                                              '%
        ForeColor = RGB(0, 0, 255)                                                 '%
        Print i                                                            '%
        ForeColor = RGB(0, 0, 0)                                                   '%
        For j = 1 To IDPNowNextRange                        ' Attention si NOWNEXTRANGE > 10, dépassement
capacité        '%
            If j = IDPNowNextRange + 1 Then xt = xt + 800 Else xt = xt + 400  '            - id -                '%
            If j > IDPNowNextRange Then ForeColor = RGB(255, 0, 0)        '            - id -                '%
            CurrentX = xt: CurrentY = yt                                           '%
            Print Element(i, j)                                                '%
        Next j                                                             '%
    ForeColor = RGB(0, 0, 0)                                                   '%
    xt = 1000: yt = yt + 245                                                   '%
    Next i                                                                 '%
'%
    GoTo Fin_Affichage_Fin_Prog                                                '%
'                                                                      '%
'                                                                          '%
Fin_Affichage_Fin_Prog:                         '                              '%
End Sub                                                                    '%
'                                                                          '%
'%%%%%%%%%%%%%%%%%%%%%%%%%  Maurice - 05-06-2008 - Fin des affichages en fin de
programme  %%%%%%%%%%%%%%%%%%%%%%%%%%%%%%%%%%%%%%
'

 '
' %%%%%%%%%%%%%%%%%%%%%%%%%%%%%%%%%%%%%%%% Maurice - 05-06-2008 - Début Calcul Table
Activity %%%%%%%%%%%%%%%%%%%%%%%%%%%%%%%%%%%%%%%
Private Sub Maurice_AffTbActivity()           ' %%%% Maurice - 03-06-2008 - affichage du tableau Activity
'%
    Esp = "Maurice_AffTbActivity"            ' Maurice 03-06-2008 - Espionnage                      '%
    Espion                          ' Maurice 03-06-2008 - Espionnage                      '%
'                                                                          '%
' En entrée Table Activity indexée par la ligne la + récente NewLineNumber et le n° de colonne k = 1 à Anumber
'%
'       Le nb de lignes de la table Activity est paramétré par EndOfActivityRegister (150 à ce jour)
'%
' En sortie Tableau 'Activity Register Line' sur feuille principale                        '%
'       La ligne la + récente de l'entrée sera mise en fin du tableau 'Activity Register Line' (ligne 10)           '%
'       On calcule les n° de ligne de la table Activity à positionner en ligne (Ln) du tableau 'Activity Register Line'
:  '%
'             Si NewLineNumber > 9      alors Ltn = NewLineNumber - 9                              '%
'             Si NewLineNumber < ou = 9 alors Ltn = EndOfActivityRegister + NewLineNumber - 9
'%
'                                                                          '%
' Positionnement des n° de ligne de la table Activity                                      '%
    If NewLineNumber > 16 Then                                                 '%
        Ln(0) = NewLineNumber - 16                                             '%
    Else                                                                   '%
        Ln(0) = EndOfActivityRegister + NewLineNumber - 16                             '%
    End If                                                                 '%
    For I13 = 1 To 15                                                          '%
        If Ln(I13 - 1) = EndOfActivityRegister Then                                    '%
            Ln(I13) = 1                                                        '%
        Else                                                               '%
```

```
        Ln(I13) = Ln(I13 - 1) + 1                                              '%
      End If                                                         '%
    Next I13                                                           '%
                                                           '%
' Positionnement des valeurs de la table Activity dans le champ        '%
' 'Activity Register Line' affiché à l'écran                      '%
'                                                        '%
Dim ColMax As Integer        '%
Dim LabelStart As Integer
LabelStart = 0
   If Anumber <= 12 Then ColMax = Anumber Else ColMax = 12      '%
   For I13 = 1 To 16                                        '%
    For I14 = 1 To 12                              '%
    LActivity((I13 - 1) * 12 + (I14 - 1)) = 0        '%
    Next I14                                        '%
    Next I13


        For I13 = (LineNumber - 15) To LineNumber                                   '%
           If I13 < 1 Then GoTo NoLine
           For I14 = 1 To ColMax  '%
              LActivity(LabelStart + (I14 - 1)) = Activity((I13), I14)         '%
           Next I14                                                '%
        LabelStart = LabelStart + 12
        If LabelStart >= (12 * 16) Then LabelStart = 0
NoLine:
        Next I13

'%
' %%%%%%%%%%%%%%%%%%%%%%%%%%%%%%%%%%% Maurice - 05-06-2008 - Fin Calcul Table
Activity %%%%%%%%%%%%%%%%%%%%%%%%%%%%%%%%%%%%%%
'
'
' %%%%%%%%%%%%%%%%%%%%%%%%%%%%%%%%%%%%%%%%%%%%%%%%%%%%%%% FIN DES PAQUETS
MAURICE %%%%%%%%%%%%%%%%%%%%%%%%%%%%%%%%%%%%%%%%%%%%%%%%%%%%%
'
End Sub

Public Sub ConnectivityExtractSub()
'This gives the number of times each element is cited in the Now fields and in the Next fields of all the Enumber
elements
'Signing is ignored!

For I98 = 1 To Enumber
ConnectivityNow(I98) = 0
ConnectivityNext(I98) = 0
Next I98

'this scoring does not take account of signs
For I98 = 1 To Enumber

   Select Case I98
   Case Is = IDP1
   If UseIDP = 0 Then GoTo IDPIsZero
   For J98 = 1 To IDPNowNextRange / 2
```

```
    If Element(I98, J98) = 0 Then GoTo ZeroProblemNow
    ConnectivityNow(Element(I98, J98)) = ConnectivityNow(Element(I98, J98)) + 1
ZeroProblemNow:
    Next J98
    Case Else
    For J98 = KNowNumber(I98) To KNowMin
    If Element(I98, J98) = 0 Then GoTo ConnectivityZeroProblem
    ConnectivityNow(Element(I98, J98)) = ConnectivityNow(Element(I98, J98)) + 1
ConnectivityZeroProblem:
    Next J98
    End Select

    Select Case I98
    Case Is = IDP1
    For J98 = (IDPNowNextRange / 2) + 1 To (IDPNowNextRange)
    If Element(I98, J98) = 0 Then GoTo ZeroProblemNext
    ConnectivityNext(Element(I98, J98)) = ConnectivityNext(Element(I98, J98)) + 1
ZeroProblemNext:
    Next J98
    Case Else
    For J98 = KNextMin To KNextNumber(I98)
    ConnectivityNext(Element(I98, J98)) = ConnectivityNext(Element(I98, J98)) + 1
    Next J98
    End Select

IDPIsZero:
Next I98

'Print

End Sub

Public Sub ConnectivityBinSub()
'There are connectivity scores for each element.
'This finds how many times a particular score occurs
'and can bin them if the denominator is set to more than 1

ZeroNow = 0
ZeroNext = 0

I98 = 0
J98 = 0

For I98 = 1 To (NowNextRange * Anumber) 'scores are unlikely to exceed this
BinNow(I98) = 0
BinNext(I98) = 0
Next I98

    For I98 = 1 To Enumber
    BinnedScore = Int((ConnectivityNow(I98)) / 1)
        Select Case BinnedScore
        Case Is <= 0
        ZeroNow = ZeroNow + 1
        Case Else
        BinNow(BinnedScore) = BinNow(BinnedScore) + 1
```

```
        End Select

        BinnedScore = Int((ConnectivityNext(I98)) / 1)
        Select Case BinnedScore
        Case Is <= 0
        ZeroNext = ZeroNext + 1
        Case Else
        BinNext(BinnedScore) = BinNext(BinnedScore) + 1
        End Select


    Next I98

End Sub

Public Sub ConnectivityDisplaySub()

    Picture3.Scale (-5, (Enumber / 10))-((100 + 20), -20) '(left x, top y) - (right x, bottom y)
    Picture3.Cls
    Picture3.FillStyle = 0

    Picture4.Scale (-5, (Enumber / 10))-((100 + 20), -20) '(left x, top y) - (right x, bottom y)
    Picture4.Cls
    Picture4.FillStyle = 0

    Picture5.Scale (-2, (30))-((100 + 20), -2) '(left x, top y) - (right x, bottom y)
    Picture5.Cls
    Picture5.FillStyle = 0

    Picture6.Scale (-2, (30))-((100 + 20), -2) '(left x, top y) - (right x, bottom y)
    Picture6.Cls
    Picture6.FillStyle = 0

    Picture3.Line (0, 1)-(100, 1) 'x-axis normal
    Picture3.Line (0, Enumber / 10)-(0, 0) 'y-axis
    Picture4.Line (0, 1)-(100, 1) 'x-axis
    Picture4.Line (0, Enumber / 10)-(0, 0) 'y-axis
    Picture5.Line (-1, 0)-(90, 0) 'x-axis 'log
    Picture5.Line (-1, 200)-(-1, 0) 'y-axis
    Picture6.Line (-1, 0)-(90, 0) 'x-axis 'log
    Picture6.Line (-1, 200)-(-1, 0) 'y-axis

 For I1 = 1 To (Enumber / 2) Step Enumber / 100
    Picture3.Line (-2, I1)-(0, I1) 'y-axis marks
    Picture4.Line (-2, I1)-(0, I1) 'y-axis marks
    Next I1

    For I1 = 1 To 100 Step 10
    Picture3.Line (I1, 0)-(I1, -3) 'x-axis marks
    Picture4.Line (I1, 0)-(I1, -3) 'x-axis marks
    Next I1

    For I1 = 1 To 100

        Select Case BinNow(I1) 'The radius of the circle is small if the bin is empty
```

```
        Case Is = 0
        Picture3.Circle (I1, BinNow(I1)), 1 / 100 'displays bins e.g., 4 lots of sizes (as determined by
ActualMassBinSize)
        'Picture5.Circle (I1, BinNow(I1)), 1 / 100
        Case Is > 0
        Picture3.Circle (I1, BinNow(I1)), 1 / 2 'displays bins e.g., 4 lots of sizes (as determined by
ActualMassBinSize)
        Picture5.Circle (I1, Log(BinNow(I1))), 1 / 2
        End Select

    Next I1

    For I1 = 1 To 100

        Select Case BinNext(I1) 'The radius of the circle is small if the bin is empty
        Case Is = 0
        Picture4.Circle (I1, BinNext(I1)), 1 / 100 'displays bins e.g., 4 lots of sizes (as determined by
ActualMassBinSize)

        Case Is > 0
        Picture4.Circle (I1, BinNext(I1)), 1 / 2 'displays bins e.g., 4 lots of sizes (as determined by
ActualMassBinSize)
        Picture6.Circle (I1, Log(BinNext(I1))), 1 / 2
        End Select

    Next I1
    'DoEvents
End Sub
```
